# Supplementary material for: Functional genomics reveal gene regulatory mechanisms underlying schizophrenia risk
Source: Nat Commun. 2019 Feb 8;10:670. doi: 10.1038/s41467-019-08666-4 (PMC6368563; doi:10.1038/s41467-019-08666-4)
Supplement: Supplementary file 1 — Supplementary Information [file 41467_2019_8666_MOESM1_ESM.pdf]

Functional genomics reveal gene regulatory mechanisms underlying schizophrenia risk

Yongxia Huo[1,4], Shiwu Li[1,2,4], Jiewei Liu[1], Xiaoyan Li[1,2] & Xiong-Jian Luo[1,2,3,*]

[1]Key Laboratory of Animal Models and Human Disease Mechanisms of the Chinese Academy of Sciences & Yunnan Province, Kunming Institute of Zoology, Chinese Academy of Sciences, Kunming, Yunnan 650223, China

[2]Kunming College of Life Science, University of Chinese Academy of Sciences, Kunming, Yunnan 650204, China

[3]Center for Excellence in Animal Evolution and Genetics, Chinese Academy of Sciences, Kunming 650223, China

[4]These authors contributed equally to this work

*To whom correspondence should be addressed; Key Laboratory of Animal Models and Human Disease Mechanisms, Kunming Institute of Zoology, Chinese Academy of Sciences, Kunming, Yunnan 650223, China; Tel: +86-871-68125413, Fax: +86-871-68125413, E-mail: luoxiongjian@mail.kiz.ac.cn.

Supplementary Discussion

We noticed that the overlapping functional SNPs identified by functional annotation and functional genomics were limited. Following reasons may account for the limited overlapping: First, functional annotation and functional genomics use different strategies and principles to prioritize the potential functional SNPs. For example, sequence conservation plays a pivotal role in functional annotation approaches (including LINSIHGT, Eigen and CADD). However, functional genomics only use Chip-Seq data from human brain tissues or neuronal cells to derive the DNA binding motifs of TFs. Second, though ChIP-Seq data were included in some functional annotation methods (including LINSIGHT, Eigen, GWAVA and RegulomeDB), the majority of ChIP-Seq data were not from brain tissues (or neuronal cells). Considering the strong tissue specificity of regulatory elements, ChIP-Seq and eQTL data from brain tissues were more proper for identifying the potential functional SNPs and target genes. Third, it should be noted that the results of functional annotation approaches were largely based on prediction (that is, these annotation methods predict the possible functional SNPs based on several features, including sequence conservation, location in ChIP-Seq peak, association with gene expression, and etc). Finally, even if the potential functional SNP prioritized by the functional annotation methods have functional consequences, the exact gene regulatory mechanisms (e.g., binding of which transcription factor was disrupted by the potential functional SNPs) of these potential functional SNPs remain unknown. Thus, we mainly focused on functional genomics in this study.

The significant enrichment of nervous system development-related genes among the target genes of the TF binding-disrupting SNPs provides further support for the neurodevelopmental hypothesis of schizophrenia[1-3]. Despite genetic study has achieved significant progress in recent

years, the pathophysiology of schizophrenia remains largely unknown. Accumulating evidence suggests that schizophrenia may be originated from abnormal brain development. Consistently, several studies have shown that some schizophrenia risk genes have a pivotal role in neurogenesis and brain development[4,5]. In fact, previous studies have showed that schizophrenia risk genes were enriched in neurodevelopmental and cell adhesion pathways[6-10]. For example, through integrating chromatin contacts with SNP associations from schizophrenia GWAS, Won *et al.* showed that schizophrenia candidate genes were enriched in neurodevelopment pathway (including transcription factors involved in neurogenesis, neuronal differentiation and chromatin remodelers)[9]. In addition to the neurodevelopment and cell adhesion pathways, ion channel pathways were also reported to be enriched in schizophrenia[11,12].

We found that the expression level of the target genes was higher in prenatal stage compared with postnatal stage, which was consistent with the findings of previous studies[10,13,14]. However, Pers *et al.* showed that schizophrenia risk genes prioritized by DEPICT approach have higher expression level in postnatal period[12]. This inconsistency may be due to the different prioritization methods used (i.e., Pers *et al.* used DEPICT to prioritize the schizophrenia risk genes, while we used functional genomics). More work is needed to validate if schizophrenia risk genes have higher expression level during prenatal stage.

We noticed that CTCF binding was frequently disrupted by the schizophrenia risk SNPs, suggesting that disruption of CTCF binding may represent a common mechanism that schizophrenia risk SNPs exert their effect on schizophrenia. CTCF is a transcriptional repressor encoded by *CTCF* gene, which has pivotal roles in transcription regulation[15]. Recent studies also showed that CTCF plays an important role in regulating the 3D structure of chromatin[16,17]. To

further explore the potential role of *CTCF* in schizophrenia, we performed eQTL annotation and identified 213 SNPs that showed significant association with *CTCF* expression in GTEx data set (nucleus accumbens (basal ganglia)) (**Supplementary Data 7**). Among the 213 SNPs, rs75760574 showed the most significant association with *CTCF* expression. We thus examined the association between rs75760574 and schizophrenia in PGC2+CLOZUK data set (40,675 cases and 64,643 controls). We found that rs75760574 showed marginal significant association with schizophrenia (*P*=0.002). Of note, a recent study showed that *CTCF* is a novel risk gene for schizophrenia[18]. Interestingly, Baruch *et al*. also found that SNPs in CTCF binding regions were associated with schizophrenia in the Jewish Ashkenazi population[19]. These results provide convergent evidence that support the potential role of *CTCF* gene in schizophrenia. However, more work is need to elucidate the role of CTCF in schizophrenia.

Binding of CTCF and POLR2A were frequently disrupted by schizophrenia risk SNPs. This may be due to the possibility that CTCF and POLR2A have more binding sites (on the genome) than other TFs. To test if disruption of CTCF and POLR2A were random, we conducted additional analyses. We first identified 8,447 SNPs that disrupted binding of 30 TFs (these 30 TFs were used in this study) through mapping a total of 968,903 SNPs (from the Illumina HumanOmni1-Quad) to the identified ChIP-Seq peaks (the identification of TF binding-disrupting SNPs was described in more detail above). We then randomly sampled 132 SNPs (the number of SNPs equals to the identified TF binding-disrupting SNPs in this study) from the 8,447 TF binding-disrupting SNPs and counted the number of the SNPs that disrupted the binding of each TF. We found that the number of POLR2A binding-disrupting SNPs observed in this study were significantly higher than random SNPs (*P*=0.013, 1,000 simulations, **Supplementary Figure 10a**), suggesting that the

frequent disruption of POLR2A binding by schizophrenia risk SNPs may not due to random effect. However, the number of CTCF binding-disrupting SNPs (observed in this study) were not significantly different from random SNPs (*P*>0.05, 1,000 simulations, **Supplementary Figure 10b**), implying that the frequent disruption of CTCF by schizophrenia risk SNPs may due to random effect. We also compared the number of motifs (appear on the genome) of the 30 TFs through counting the identified ChIP-Seq peaks of each TF. We found that RAD21, EP300 and POLR2A have the largest number of ChIP-Seq peaks on the genome (**Supplementary Figure 10c**). In addition, the number of ChIP-Seq peaks of CTCF were also larger than many other TFs (including GATA3, REST, TAF1 and YY1). These results suggest that schizophrenia risk SNPs tend to disrupt POLR2A binding (but not CTCF). However, more work is need to further verify this.

Supplementary Table 1. The most possible functional SNPs identified by different annotation approaches (credible causal SNPs were from the study of Pardinas *et al*[20].)

Index SNP (or	CADD[a]	Eigen[b]	GWAVA[c]	LINSIGHT[d]	RegulomeDB[e]
rs10148671	**rs10148671(7.743)**	**rs10148671(0.082)**	**rs10148671(0.3)**	**rs10148671(0.04)**	**rs10148671(6)**
rs1042992	rs13266943(8.405)	rs17310286(0.621)	rs7840859(0.49)	rs3808566(0.62)	rs71517887(4)
rs10783624	**rs10783619(5.567)**	rs10783624(-0.125)	rs2060614(0.52)	**rs10783619(0.05)**	**rs10783619(3a)**
rs1080500	**rs1080500(14.34)**	rs2246556(0.619)	**rs1080500(0.46)**	**rs1080500(0.28)**	rs2257216(2b)
rs10985817	**rs2304390(15.60)**	**rs2304390(0.683)**	rs7867465(0.5)	rs3780446(0.44)	rs7867834(2b)
rs11993663	**rs73191547(7.284)**	**rs73191547(0.763)**	rs12548428(0.43)	**rs73191547(0.16)**	rs11787370(4)
rs12129719	**rs4077431(17.89)**	**rs4077431(1.234)**	*rs4133072(0.4)*	**rs4077431(0.12)**	*rs4133072(3a)*
rs12447542	**rs12447542(4.935)**	**rs12447542(0.413)**	**rs12447542(0.19)**	**rs12447542(0.17)**	**rs12447542(6)**
rs12712510	rs11894059(13.32)	rs13031296(1.121)	rs11886927(0.44)	rs1509392(0.94)	rs12996247(3a)
rs13121251	rs10011128(15.94)	rs17016967(0.827)	**rs1444950(0.56)**	**rs1444950(0.25)**	rs13145393(3a)
rs1353545	**rs1353545(17.89)**	**rs1353545(1.55)**	**rs1353545(0.53)**	**rs1353545(0.97)**	rs9867642(2a)
rs150437760	rs112952088(22.4)	rs80087397(1.617)	**rs113062316(0.52)**	**rs113062316(0.96)**	rs1253107(1f)
rs17465671	*rs6498914(8.890)*	*rs6498914(0.502)*	rs7193419(0.48)	**rs11648018(0.05)**	**rs11648018(6)**
rs1765142	**rs571174(16.50)**	rs11031024(0.656)	rs61626579(0.49)	**rs571174(0.20)**	rs4531442(2c)
rs198160	**rs28669242(6.798)**	**rs28669242(0.588)**	rs57846780(0.37)	**rs28669242(0.05)**	rs198160(6)
rs2077586	**rs112085582(16.29**	**rs112085582(1.71)**	rs2077586(0.55)	**rs112085582(0.76)**	**rs112085582(2b)**
rs2161711	rs4788793(5.429)	rs918754(0.149)	rs75092065(0.36)	**rs889704(0.05)**	**rs889704(1f)**
rs2410572	**rs876983(16.44)**	**rs876983(0.483)**	rs13259407(0.33)	**rs876983(0.27)**	**rs876983(2b)**
rs254782	**rs34565637(13.42)**	**rs34565637(1.33)**	**rs34565637(0.74)**	rs34087837(0.87)	rs114843810(4)
rs281299	NA	NA	NA	**rs281299(0.04)**	**rs281299(7)**
rs28374258	rs9427501(14.30)	**rs2025512(0.528)**	**rs2025512(0.5)**	rs1454344(0.11)	**rs2025512(4)**
rs2917569	rs2917570(10.05)	NA	rs1939498(0.37)	rs2509230(0.16)	rs3018405(3a)
rs312477	**rs3774417(18.41)**	**rs3774417(1.518)**	rs312476(0.41)	**rs3774417(0.37)**	rs312475(4)
rs34179565	rs12433255(17.70)	rs7140259(1.290)	rs7142530(0.53)	rs2300861(0.44)	rs34179565(5)
rs35736453	rs3903663(8.721)	rs12055782(1.010)	rs28360516(0.5)	rs1342646(0.83)	rs7742212(4)
rs36104021	**rs4764719(18.88)**	**rs4764719(0.967)**	**rs4764719(0.46)**	**rs4764719(0.91)**	**rs4764719(2b)**
rs4470825	**rs147360061(4.174**	**rs147360061(0.049**	**rs147360061(0.5)**	**rs147360061(0.05)**	rs628629(5)
rs489939	**rs308692(18.27)**	**rs308692(1.141)**	rs523441(0.46)	rs518205(0.33)	rs514683(3a)
rs55669358	**rs55916192(14.63)**	**rs55916192(0.621)**	rs74427054(0.56)	rs55669358(0.85)	rs72634609(6)
rs56145559	rs6546839(17.18)	**rs7598396(1.049)**	**rs7598396(0.54)**	**rs7598396(0.31)**	rs10496191(1f)
rs56775891	**rs7238071(5.169)**	rs11664298(-0.082)	rs56775891(0.48)	rs6650631(0.06)	**rs7238071(5)**
rs58950470	rs1193851(23.0)	**rs7944860(1.60)**	rs58950470(0.51)	**rs7944860(0.41)**	rs1787034(2a)
rs6035706	**rs6113023(8.008)**	rs6137162(0.267)	rs963487(0.42)	**rs6113023(0.35)**	**rs6113023(5)**
rs634940	rs10944593(16.94)	**rs11970730(0.716)**	rs1346296(0.39)	**rs11970730(0.12)**	rs582993(3a)
rs6678676	**rs4915424(3.493)**	**rs4915424(0.0057)**	rs6678676(0.34)	**rs4915424(0.10)**	rs7517753(4)
rs6680011	**rs7547222(9.705)**	**rs7547222(0.434)**	*rs4140419(0.45)*	rs6680011(0.05)	*rs4140419(4)*
rs66885728	**rs28591622(12.62)**	rs57433322(0.70)	rs28582509(0.49)	**rs28591622(0.28)**	rs1533034(2b)
rs6701877	**rs767053(19.17)**	**rs767053(1.718)**	**rs767053(0.58)**	**rs767053(0.91)**	rs10494491(1f)
rs6800435	**rs71316429(21.4)**	**rs71316429(1.557)**	**rs71316429(0.74)**	**rs71316429(0.85)**	rs57922516(3a)
rs7225476	**rs7221014(19.19)**	**rs7221014(1.041)**	rs7218776(0.43)	**rs7221014(0.37)**	rs8079792(1f)
rs72342102	rs2161972(15.37)	**rs9373388(1.043)**	rs9376742(0.5)	**rs9373388(0.14)**	**rs9373388(2b)**
rs72769124	rs6429121(5.770)	rs10802742(-0.088)	**rs28670059(0.35)**	**rs28670059(0.05)**	**rs28670059(4)**
rs72986630	**rs72986630(10.86)**	**rs72986630(0.882)**	**rs72986630(0.4)**	**rs72986630(0.06)**	**rs72986630(2a)**
rs760608	**rs9384900(16.23)**	**rs9384900(0.620)**	rs1337332(0.55)	*rs7763992(0.16)*	*rs7763992(5)*
rs7632921	**rs62244860(14.54)**	**rs62244860(1.626)**	**rs62244860(0.63)**	**rs62244860(0.87)**	rs6764416(3a)
rs77853293	rs8188099(10.90)	rs1962832(0.502)	rs7708897(0.51)	rs7705285(0.08)	rs11739291(2b)
rs783540	rs2567636(13.02)	**rs4779050(0.918)**	rs7178350(0.48)	**rs4779050(0.29)**	rs10906984(1f)
rs893949	**rs4937884(2.373)**	NA	**rs4937884(0.29)**	**rs4937884(0.06)**	rs893949(2b)
rs9545047	**rs9545067(15.55)**	**rs9545067(1.446)**	rs9601242(0.58)	rs56911077(0.86)	rs943709(2b)
rs9881798	**rs9842181(14.42)**	**rs9842181(0.561)**	**rs9842181(0.37)**	rs748832(0.38)	rs9882532(3a)

Scores or ratings of each annotation method are shown in the parentheses. [a-e]For CADD, Eigen, GWAVA and LINSIGHT, the larger the score, the higher probability that the SNP is functional. For RegulomeDB, smaller rating suggests higher probability that the SNP is functional. The top functional SNP prioritized by at least two independent annotation approaches were shown in bold.

Supplementary Table 2. The most possible functional SNPs identified by different annotation approaches (credible causal SNPs were from the study of Li *et al*[21].)

Index_SNP (or variant)	CADD[a]	Eigen[b]	GWAVA[c]	LINSIGHT[d]	RegulomeDB[e]
rs10148671	**rs2333478(17.36)**	**rs2333478(1.526)**	rs75131751(0.65)	rs76033949(0.90)	rs932607(3a)
rs10510653	**rs62244394(15.95)**	**rs62244394(1.996)**	**rs62244394(0.62)**	rs76150980(0.55)	**rs62244394(2b)**
rs10940346	rs555704(14.92)	**rs28528780(1.351)**	**rs28528780(0.57)**	rs7341010(0.88)	rs3849669(1f)
rs111364339	rs59113396(12.71)	rs3816652(0.818)	rs77788000(0.57)	rs16918239(0.31)	rs113960550(2a)
rs11534004	**rs7778798(20.5)**	rs17682018(1.352)	**rs7778798(0.55)**	**rs7778798(0.94)**	rs2905232(3a)
rs11722779	rs4699046(16.49)	**rs223389(2.891)**	**rs223389(0.64)**	rs4699033(0.58)	rs227361(1b)
rs160593	**rs314263(22.5)**	**rs314263(1.366)**	**rs314263(0.66)**	**rs314263(0.52)**	rs12213967(2b)
rs17687067	**rs11555738(12.58)**	**rs11555738(1.297)**	rs117220747(0.46)	*rs3808645(0.21)*	*rs3808645(2b)*
rs2073499	**rs2236950(20.1)**	rs2073499(1.824)	**rs2236950(0.77)**	**rs2236950(0.98)**	rs2624842(1d)
rs2247870	rs11738573(13.54)	**rs2438345(1.445)**	**rs2438345(0.57)**	**rs2438345(0.39)**	**rs2438345(2a)**
rs2383377	**rs11156763(7.248)**	*rs61981393(0.384)*	rs60535335(0.41)	*rs61981393(0.07)*	**rs11156763(6)**
rs2764766	rs151882(13.66)	rs13173755(0.434)	rs245179(0.47)	rs245183(0.34)	rs4836348(1d)
rs28607014	rs2393207(10.24)	**rs9658255(0.297)**	**rs9658255(0.51)**	rs2293051(0.19)	rs561712(3a)
rs28735056	rs34977373(12.79)	**rs2012687(0.429)**	**rs2012687(0.48)**	rs3744881(0.16)	rs4799092(2b)
rs323167	**rs4727751(19.40)**	**rs4727751(1.591)**	rs323176(0.53)	**rs4727751(0.93)**	rs322004(2b)
rs35065479	**rs4793885(19.61)**	**rs4793885(1.228)**	rs4793560(0.48)	rs16942143(0.66)	rs12936628(3a)
rs4479915	rs12192875(12.11)	rs11753244(0.915)	rs903791(0.48)	rs71569466(0.48)	rs9347899(4)
rs56007784	rs35640767(10.79)	rs28365859(1.415)	rs16945571(0.46)	rs4790360(0.13)	rs12601838(2b)
rs56775891	rs7238071(5.169)	**rs11081564(0.556)**	rs56775891(0.48)	**rs11081564(0.08)**	**rs11081564(2b)**
rs62152284	**rs4851036(20.6)**	**rs4851036(0.987)**	**rs4851036(0.58)**	**rs4851036(0.43)**	rs73946422(4)
rs6430491	*rs10928478(15.01)*	**rs1812665(1.490)**	rs764686(0.45)	*rs10928478(0.17)*	**rs1812665(2b)**
rs6500596	**rs3747579(26.7)**	*rs709632(2.634)*	**rs3747579(0.67)**	*rs709632(0.94)*	rs4238849(1b)
rs6903570	rs9353806(22.1)	**rs34848022(1.478)**	rs9342117(0.52)	**rs34848022(0.95)**	rs9344660(2b)
rs72843506	rs9908032(23.7)	rs9898948(0.763)	**rs9894159(0.59)**	**rs9894159(0.61)**	rs16960717(1f)
rs73219805	rs73217710(9.487)	rs17310286(0.621)	rs1048527(0.62)	rs3808566(0.62)	rs17055142(4)
rs758129	rs17803620(23.4)	rs150299(1.482)	rs2283432(0.57)	rs2246900(0.59)	rs961288(2a)
rs7757969	**rs9384804(19.30)**	**rs9384804(1.154)**	rs6919306(0.59)	rs12055398(0.29)	rs58691769(2a)
rs783540	**rs11630197(26.8)**	*rs4779050(0.917)*	rs11631813(0.56)	*rs4779050(0.29)*	**rs11630197(1c)**
rs8012642	**rs4144367(20.8)**	**rs4144367(0.829)**	rs8021077(0.53)	**rs4144367(0.25)**	rs28637508(3a)
rs8058130	**rs12920359(17.36)**	**rs12920359(1.194)**	rs318202(0.47)	**rs12920359(0.34)**	rs650856(3a)
rs999494	**rs61152410(19.52)**	**rs61152410(1.20)**	rs2077586(0.55)	**rs61152410(0.44)**	rs34419497(4)

Scores or ratings of each annotation method are shown in the parentheses. [a-e]For CADD, Eigen, GWAVA and LINSIGHT, the larger the score, the higher probability that the SNP is functional. For RegulomeDB, smaller rating suggests higher probability that the SNP is functional. The top functional SNP prioritized by at least two independent annotation approaches were shown in bold.

Supplementary Table 3. Brain tissues and number of subjects used in GTEx.

Tissue	Number of samples with genotypes and expression
Brain - Cerebellum	154
Brain - Caudate (basal ganglia)	144
Brain - Cortex	136
Brain - Nucleus accumbens (basal ganglia)	130
Brain - Cerebellar Hemisphere	125
Brain - Frontal Cortex (BA9)	118
Brain - Putamen (basal ganglia)	111
Brain - Hippocampus	111
Brain - Anterior cingulate cortex (BA24)	109
Brain - Hypothalamus	108
Brain - Amygdala	88
Brain - Spinal cord (cervical c-1)	83
Brain - Substantia nigra	80

Supplementary Table 4. ChIP-Seq experiments and TFs used in this study.

Brain tissues and neuronal cell lines	Lab	Data source	TFs	The name of biosamples in this paper
Astrocyte of the cerebellum	John Stamatoyannopoulos, UW	ENCODE	CTCF	Astrocyte of the cerebellum
BE2C	John Stamatoyannopoulos, UW	ENCODE	CTCF	BE2C
Brain microvascular endothelial cell	John Stamatoyannopoulos, UW	ENCODE	CTCF	Brain microvascular endothelial cell
Choroid plexus epithelial cell	John Stamatoyannopoulos, UW	ENCODE	CTCF	Choroid plexus epithelial cell
H54	Vishwanath Iyer, UTA	ENCODE	CTCF, POLR2A	H54
Medulloblastoma	Vishwanath Iyer, UTA	ENCODE	CTCF	Medulloblastoma
Neural cell derived from H1-hESC	Michael Snyder, Stanford	ENCODE	CTCF, MXI1, RAD21, SMC3	Neural-M
Neural cell[a]	Bradley Bernstein, Broad	ENCODE	EZH2	Neural-B
Neural cell[a]	Richard Myers, HAIB	ENCODE	POLR2A, REST, TAF1	Neural-R
PFSK-1[b]	Richard Myers, HAIB	ENCODE	FOXP2, REST	PFSK-1
PFSK-1[b]	Richard Myers, HAIB	ENCODE	POLR2A, REST, SIN3A, TAF1	PFSK-1-1
SH-SY5Y	Peggy Farnham, USC	ENCODE	GATA2, GATA3	SH-SY5Y
SK-N-MC	Bradley Bernstein, Broad	ENCODE	EZH2	SK-N-MC-B
SK-N-MC	Richard Myers, HAIB	ENCODE	FOXP2, POLR2A	SK-N-MC-R
SK-N-SH	Bradley Bernstein, Broad	ENCODE	CTCF, EZH2	SK-N-SH-B
SK-N-SH	John Stamatoyannopoulos, UW	ENCODE	CTCF	SK-N-SH-J
SK-N-SH	Michael Snyder, Stanford	ENCODE	CTCF, EP300, JUND, MXI1, NRF1, RAD21,	SK-N-SH-M
SK-N-SH	Michael Snyder, Stanford	ENCODE	CHD2, IRF3, RCOR1, USF2	SK-N-SH-M-1
SK-N-SH	Richard Myers, HAIB	ENCODE	ELF1, EP300, FOSL2, FOXM1, GABPA, GATA3,	SK-N-SH-R
SK-N-SH	Richard Myers, HAIB	ENCODE	POLR2A, REST, SIN3A, TAF1	SK-N-SH-R-1
SK-N-SH	Richard Myers, HAIB	ENCODE	CTCF, EP300, RAD21, USF1, YY1	SK-N-SH-R-2

[a]Nerual cell: In vitro differentiated neural cells from male embryo. [b]PFSK-1 is a neuroectodermal cell line derived from a human cerebral brain tumor.

Supplementary Table 5. Association significance between the TF binding-disrupting SNPs and gene expression in human brain tissues

SNP	Gene	P (CMC)	FDR (CMC)	P (LIBD)	FDR (LIBD)	P (GTEx)
rs10038801	*SLCO4C1*	6.32E-27	3.89E-24	7.70E-14	2.34E-11	NA
rs10795	*DNM1P51*	NA	NA	3.65E-06	3.28E-04	1.40E-06 (Front Cor)
	GOLGA2P7	1.63E-17	6.37E-15	1.07E-09	1.88E-07	4.50E-13(Cau)
	NMB	6.34E-04	2.33E-02	NA	NA	8.10E-06 (Cau)
	WDR73	1.05E-03	3.48E-02	1.03E-05	8.29E-04	NA
rs10852932	*SRR*	5.79E-20	5.57E-18	2.87E-10	5.50E-08	NA
rs11861362	*TSNAXIP1*	4.77E-04	2.73E-02	NA	NA	1.00E-07 (ACC)
rs12114661	*TNKS*	7.94E-06	5.34E-04	1.40E-08	2.06E-06	NA
rs12136320	*TMEM81*	2.97E-05	2.00E-03	9.91E-06	7.98E-04	1.10E-06 (Putamen)
	TMCC2	4.30E-07	4.34E-05	1.68E-04	9.27E-03	NA
	RBBP5	5.87E-09	8.26E-07	NA	NA	8.80E-08 (NAc)
rs12416331	*CNNM2*	1.37E-04	6.09E-03	1.31E-06	1.31E-04	NA
rs12912934	*CSPG4P11*	3.11E-44	1.21E-40	NA	NA	3.20E-07 (Cere)
	EFTUD1P1	4.07E-13	9.63E-11	8.48E-07	8.84E-05	1.10E-05 (Hyp)
	CSPG4P12	3.36E-33	4.92E-30	NA	NA	3.40E-07 (Front Cor)
	DNM1P51	9.84E-05	4.96E-03	5.54E-10	1.02E-07	2.50E-11 (Front Cor)
	GOLGA6L5	NA	NA	4.94E-07	5.41E-05	1.30E-07 (Cortex)
	GOLGA2P7	2.75E-28	2.91E-25	9.30E-19	4.52E-16	1.20E-24(Cau)
rs13113099	*KRT8P46*	1.12E-11	1.56E-09	NA	NA	1.30E-05 (Cere Hemi)
	LRRC37A15P	3.76E-12	5.59E-10	NA	NA	3.40E-07 (Cere Hemi)
	BDH2	8.59E-07	5.83E-05	1.16E-04	6.78E-03	NA
rs1321	*ALG12*	9.34E-07	8.95E-05	NA	NA	5.50E-07 (Cau)
rs138833	*ALG12*	1.80E-06	1.63E-04	NA	NA	1.10E-05 (Cau)
rs159961	*RERE*	6.39E-04	2.74E-02	5.93E-10	1.08E-07	NA
rs16937	*TMEM81*	1.06E-05	8.06E-04	3.64E-10	6.88E-08	9.70E-07 (Putamen)
	RBBP5	1.16E-09	1.84E-07	1.53E-04	8.56E-03	2.70E-09 (NAc)
rs1801311	*LINC00634*	2.40E-08	3.08E-06	1.91E-08	2.73E-06	NA
	CYP2D7P	NA	NA	4.17E-12	1.03E-09	1.10E-05 (NAc)
	NAGA	4.06E-17	1.43E-14	4.46E-29	4.42E-26	5.70E-10 (Cere)
	FAM109B	1.46E-06	1.34E-04	6.23E-17	2.58E-14	6.30E-06 (Cortex)
rs223387	*LRRC37A15P*	2.84E-13	4.96E-11	NA	NA	1.80E-09 (Cere Hemi)
rs223390	*LRRC37A15P*	2.58E-13	4.55E-11	1.43E-04	8.10E-03	2.30E-09 (Cere Hemi)
	BDH2	5.34E-07	3.75E-05	1.37E-04	7.80E-03	NA
rs2269524	*LINC00634*	2.33E-08	3.01E-06	1.01E-09	1.78E-07	NA
	CYP2D7P	NA	NA	8.48E-09	1.29E-06	1.10E-05 (NAc)
	NAGA	5.33E-18	2.03E-15	1.04E-26	8.81E-24	2.90E-09 (Cere)
	SMDT1	NA	NA	3.91E-06	3.49E-04	4.90E-30 (Cere)
	FAM109B	1.02E-06	9.73E-05	7.35E-16	2.77E-13	5.80E-06 (Cortex)
rs2270363	*CORO7*	2.06E-10	5.06E-08	1.72E-17	7.46E-15	8.90E-13 (ACC)
	NMRAL1	1.62E-10	4.05E-08	4.88E-16	1.87E-13	9.00E-41 (Cere Hemi)
	DNAJA3	8.22E-31	1.17E-27	2.21E-06	2.09E-04	NA
	CDIP1	3.05E-07	4.40E-05	1.43E-05	1.11E-03	5.10E-34 (Cere)
rs2304204	*CPT1C*	8.51E-14	2.55E-11	4.56E-05	3.05E-03	2.00E-05 (Cere)
	IRF3	1.50E-05	1.30E-03	NA	NA	1.90E-07 (Cere Hemi)
rs2304206	*CPT1C*	2.57E-13	7.33E-11	1.38E-06	1.38E-04	NA
	IRF3	1.27E-05	1.13E-03	NA	NA	5.10E-08 (Cere Hemi)
rs2385395	*CNPPD1*	4.21E-06	3.11E-04	1.10E-06	1.12E-04	4.10E-11 (Spinal cord)
rs2535629	*MUSTN1*	1.93E-05	1.36E-03	6.38E-10	1.16E-07	NA
	GNL3	NA	NA	5.81E-07	6.27E-05	4.80E-08 (Cere)
	NEK4	2.81E-10	5.30E-08	4.59E-06	4.03E-04	NA
rs2551945	*METTL21A*	4.54E-16	1.52E-13	1.12E-08	1.67E-06	NA
	CREB1	1.24E-08	1.49E-06	7.48E-05	4.66E-03	NA
rs2711116	*DFNA5*	5.07E-04	1.88E-02	6.63E-08	8.62E-06	2.00E-07 (Cortex)
rs281759	*FTCDNL1*	2.98E-13	6.79E-11	3.92E-11	8.51E-09	2.00E-06 (Hyp)
	TYW5	2.25E-09	3.07E-07	1.80E-06	1.74E-04	5.60E-09 (Cere Hemi)
rs2856268	*RLBP1*	2.58E-12	5.56E-10	1.28E-08	1.89E-06	6.50E-06 (Cortex)

	POLG	5.20E-05	2.87E-03	4.47E-06	3.93E-04	7.20E-09 (Cere)
rs28633410	*LINC00634*	3.33E-07	3.53E-05	2.30E-06	2.17E-04	NA
	NAGA	4.05E-17	1.43E-14	2.92E-22	1.86E-19	NA
	FAM109B	4.52E-07	4.66E-05	3.40E-06	3.08E-04	NA
rs2974999	*TOM1L2*	6.61E-14	4.75E-12	1.20E-04	6.97E-03	2.90E-06 (Cere)
rs301792	*RERE*	5.90E-04	2.57E-02	3.65E-07	4.10E-05	NA
rs3131340	*ZNF192P1*	2.19E-05	1.00E-03	NA	NA	7.40E-08 (Cere)
	ZNF323	1.75E-04	6.31E-03	NA	NA	2.20E-05 (Spinal cord)
	ZNF193	1.63E-04	5.93E-03	NA	NA	1.70E-05 (Cere)
rs340836	*PROX1-AS1*	9.71E-06	7.46E-04	3.16E-07	3.60E-05	3.30E-07 (Cere)
rs3743078	*CHRNA5*	NA	NA	1.12E-10	2.28E-08	2.20E-06 (Putamen)
rs3773744	*TKT*	1.91E-05	1.35E-03	NA	NA	1.90E-07 (Spinal cord)
rs3773745	*TKT*	1.92E-05	1.35E-03	NA	NA	1.90E-07 (Spinal cord)
rs3794993	*YJEFN3*	3.87E-04	2.26E-02	9.66E-10	1.71E-07	NA
	GATAD2A	2.83E-35	4.68E-32	4.76E-12	1.17E-09	NA
rs3814880	*INO80E*	2.28E-13	7.83E-11	7.60E-29	7.44E-26	3.30E-10 (Cere)
	MAPK3	8.56E-07	1.10E-04	1.01E-06	1.03E-04	NA
	YPEL3	NA	NA	5.52E-06	4.74E-04	4.80E-08 (Cere)
rs3822346	*PCDHA10*	1.96E-14	4.50E-12	1.30E-13	3.86E-11	1.40E-11 (Front Cor)
	PCDHA13	3.90E-19	1.49E-16	3.21E-14	1.02E-11	4.90E-10 (Cere Hemi)
	PCDHA1	NA	NA	2.62E-07	3.03E-05	1.00E-09 (Cere)
	PCDHA2	3.69E-08	3.96E-06	3.96E-06	3.53E-04	6.80E-08 (Cere)
	PCDHA7	9.78E-20	4.04E-17	1.30E-14	4.29E-12	5.80E-09 (Spinal cord)
	PCDHA8	2.43E-18	8.29E-16	5.03E-32	5.90E-29	3.20E-07 (Cere Hemi)
	PCDHA9	NA	NA	6.19E-18	2.80E-15	6.90E-07 (Cere Hemi)
	ZMAT2	1.30E-10	2.00E-08	1.10E-08	1.65E-06	NA
	WDR55	9.48E-04	3.35E-02	NA	NA	5.00E-06 (Cere)
	IK	8.96E-04	3.20E-02	3.49E-05	2.41E-03	NA
	TMCO6	NA	NA	8.82E-05	5.37E-03	1.20E-07 (Cere)
rs393223	*KRT8P46*	1.06E-16	2.61E-14	NA	NA	2.60E-06 (ACC)
	LRRC37A15P	2.62E-18	7.13E-16	NA	NA	6.10E-08 (Cere Hemi)
rs4785581	*C16orf55*	5.16E-15	2.09E-12	NA	NA	1.70E-06 (Cortex)
rs4786494	*CORO7*	2.78E-10	6.71E-08	2.57E-20	1.42E-17	3.10E-13 (ACC)
	NMRAL1	4.49E-11	1.20E-08	NA	NA	4.30E-39 (Cere Hemi)
	CDIP1	1.17E-07	1.84E-05	NA	NA	8.80E-29 (Cere)
rs486781	*FAM86B3P*	4.91E-28	4.67E-25	NA	NA	3.60E-14 (Cere)
rs4924832	*TOM1L2*	5.31E-17	4.43E-15	NA	NA	1.30E-06 (Cere)
rs5751195	*OLA1P1*	NA	NA	7.24E-05	4.53E-03	6.10E-06 (Sub nig)
	LINC00634	5.05E-09	7.29E-07	3.41E-14	1.08E-11	NA
	NAGA	6.64E-19	2.71E-16	1.49E-23	1.03E-20	1.80E-21 (Cere)
	WBP2NL	2.05E-41	4.08E-38	7.53E-12	1.81E-09	2.70E-09 (ACC)
	FAM109B	7.52E-08	8.87E-06	2.24E-06	2.12E-04	NA
	CYP2D6	NA	NA	8.60E-11	1.78E-08	2.00E-15 (Hyp)
rs6002621	*LINC00634*	2.45E-08	3.14E-06	2.70E-07	3.13E-05	NA
	NAGA	2.19E-17	7.87E-15	1.87E-23	1.29E-20	2.60E-09 (Cere)
	SMDT1	NA	NA	2.04E-05	1.51E-03	6.60E-29 (Cere)
	FAM109B	3.09E-06	2.67E-04	3.53E-15	1.24E-12	3.40E-06 (Cortex)
rs61660810	*NOS2P3*	NA	NA	1.06E-06	1.09E-04	8.60E-08 (NAc)
	USP32P3	5.82E-10	3.21E-08	1.40E-04	7.96E-03	2.00E-06 (Cortex)
	CCDC144C	4.37E-20	4.22E-18	NA	NA	1.30E-05 (Putamen)
rs6795127	*TKT*	1.37E-04	7.36E-03	NA	NA	2.40E-09 (Spinal cord)
rs6992091	*FAM85B*	NA	NA	1.75E-08	2.53E-06	2.40E-09 (Cortex)
	FAM86B3P	5.79E-29	5.69E-26	7.93E-29	7.76E-26	7.30E-10 (Front Cor)
rs7012106	*FAM83H*	3.07E-15	1.10E-12	NA	NA	1.50E-10 (Cere)
rs7014953	*FAM85B*	NA	NA	3.69E-08	5.02E-06	1.40E-09 (Cortex)
	FAM86B3P	2.19E-29	2.19E-26	1.01E-26	8.58E-24	3.10E-09 (Front Cor)
rs72748702	*CSPG4P11*	4.48E-42	1.36E-38	NA	NA	1.20E-07 (Cere)
	EFTUD1P1	7.36E-14	1.95E-11	2.07E-06	1.97E-04	1.40E-05 (Hyp)
	CSPG4P12	2.54E-31	3.34E-28	NA	NA	3.80E-07 (Front Cor)
	DNM1P51	1.33E-04	6.41E-03	5.39E-09	8.46E-07	2.20E-11 (Front Cor)

	GOLGA6L5	NA	NA	1.01E-06	1.04E-04	2.00E-07 (Cortex)
	GOLGA2P7	1.40E-27	1.40E-24	2.36E-18	1.11E-15	1.90E-24(Cau)
rs7579996	*SEPT10*	2.67E-20	1.36E-17	3.37E-08	4.63E-06	NA
rs778593	*PCDHA10*	5.19E-10	7.48E-08	1.50E-12	3.93E-10	2.40E-08 (Front Cor)
	PCDHA13	1.06E-12	2.08E-10	2.60E-10	5.03E-08	4.00E-06 (Cere)
	SRA1	2.73E-08	3.00E-06	1.41E-04	8.02E-03	NA
	PCDHA1	NA	NA	2.02E-08	2.89E-06	3.60E-10 (Cere)
	PCDHA2	2.45E-08	2.71E-06	NA	NA	1.10E-07 (Cere)
	PCDHA7	1.55E-13	3.29E-11	3.34E-12	8.39E-10	NA
	PCDHA8	4.71E-14	1.04E-11	6.25E-21	3.61E-18	9.10E-06 (Cere Hemi)
	ZMAT2	6.87E-10	9.75E-08	1.28E-08	1.90E-06	2.40E-06 (ACC)
	NDUFA2	7.05E-07	6.08E-05	2.01E-06	1.92E-04	NA
	WDR55	1.27E-04	6.37E-03	3.83E-05	2.62E-03	1.90E-06 (Cere)
	IK	8.00E-04	2.92E-02	1.52E-04	8.52E-03	NA
	TMCO6	NA	NA	1.76E-06	1.71E-04	3.80E-15 (Cere)
rs78532287	*USP32P3*	4.48E-10	2.49E-08	9.14E-13	2.46E-10	5.60E-06 (NAc)
	CCDC144C	6.20E-17	5.16E-15	2.90E-11	6.42E-09	1.70E-06 (Putamen)
rs796364	*FTCDNL1*	1.01E-13	2.46E-11	9.50E-09	1.44E-06	5.80E-07 (Hyp)
	TYW5	1.28E-08	1.54E-06	1.99E-06	1.91E-04	2.50E-08 (Cere Hemi)
rs8135801	*LINC00634*	1.39E-08	1.85E-06	3.62E-08	4.94E-06	NA
	CYP2D7P	NA	NA	4.45E-12	1.10E-09	6.70E-06 (NAc)
	NAGA	1.14E-17	4.22E-15	6.67E-29	6.55E-26	5.10E-09 (Cere)
	FAM109B	6.60E-07	6.55E-05	3.84E-17	1.62E-14	6.30E-06 (Cortex)
rs910800	*ALG12*	5.31E-07	5.40E-05	NA	NA	5.60E-07 (Cau)
rs9306356	*CENPM*	NA	NA	1.03E-07	1.29E-05	1.20E-05 (Cere Hemi)
rs9362397	*C6orf162*	3.16E-21	1.01E-18	4.47E-25	3.42E-22	7.70E-17 (Cere)

Brain tissues from the dorsolateral prefrontal cortex (DLPFC) were used in CMC (N=467) and LIBD brain eQTL browser (N=412). Tissues from 8 brain regions were used in GTEx. Front Cor: Frontal Cortex; Cau: Caudate; ACC:Anterior cingulate cortex; NAc: Nucleus accumbens; Cere:Cerebellum; Hyp: Hypothalamus; Cere Hemi:Cerebellar Hemisphere; Sub nig: Substantia nigra. NA, not available. SNPs that showed significant association with the expression of the same gene in at least two independent eQTL datasets were shown.

Supplementary Table 6. The linkage disequilibrium values between the TF binding-disrupting SNPs

ORSID[a]	RSID[b]	CHR[c]	POS1[d]	POS2[e]	DIST[f]	R2[g]
rs10083370	rs10083367	14	104314182	104314183	1	1
rs11986122	rs11993089	8	10009949	10009952	3	0.995827
rs12114661	rs11986122	8	9573154	10009949	436795	0.463898
rs12114661	rs11993089	8	9573154	10009952	436798	0.466285
rs1321	rs9616378	22	50297435	50311973	14538	0.888207
rs1321	rs9616382	22	50297435	50312979	15544	0.888207
rs1321	rs7410601	22	50297435	50317187	19752	0.940927
rs138833	rs910800	22	50178917	50278642	99725	0.852402
rs138833	rs1321	22	50178917	50297435	118518	0.863565
rs138833	rs9616378	22	50178917	50311973	133056	0.760177
rs138833	rs9616382	22	50178917	50312979	134062	0.760177
rs138833	rs7410601	22	50178917	50317187	138270	0.80946
rs16937	rs12136320	1	205035455	205110946	75491	0.729726
rs1801311	rs6002621	22	42486723	42511002	24279	0.991128
rs1801311	rs28633410	22	42486723	42527471	40748	0.880251
rs2027349	rs72694957	1	150039678	150135291	95613	0.932557
rs223387	rs13113099	4	103749516	103974122	224606	0.341826
rs223390	rs223387	4	103748797	103749516	719	0.996031
rs223390	rs13113099	4	103748797	103974122	225325	0.344707
rs2269524	rs1801311	22	42475703	42486723	11020	0.986732
rs2269524	rs6002621	22	42475703	42511002	35299	0.977899
rs2269524	rs28633410	22	42475703	42527471	51768	0.86787
rs2304206	rs2304204	19	50168871	50169020	149	1
rs2905232	rs7796648	7	113392567	113460326	67759	0.869758
rs301792	rs159961	1	8468278	8484228	15950	0.986171
rs321964	rs322004	7	78315591	78332416	16825	0.610739
rs34455584	rs1805579	3	180630191	180630565	374	0.931237
rs3773744	rs3773745	3	53270344	53270424	80	1
rs3773744	rs6795127	3	53270344	53376669	106325	0.559027
rs3773745	rs6795127	3	53270424	53376669	106245	0.559027
rs3773745	rs6795127	3	53270424	53376669	106245	0.559027
rs393223	rs223390	4	103605916	103748797	142881	0.374285
rs393223	rs223387	4	103605916	103749516	143600	0.376078
rs393223	rs13113099	4	103605916	103974122	368206	0.305892
rs4759413	rs76514049	12	123743436	123752637	9201	1
rs4759413	rs60754073	12	123743436	123849272	105836	1
rs4759413	rs28594416	12	123743436	123849921	106485	1
rs4786494	rs2270363	16	4506232	4526292	20060	0.961428
rs485315	rs308699	3	161473229	161488369	15140	0.69311
rs4924832	rs2974999	17	17897739	17997547	99808	0.86885
rs494791	rs11233566	11	82996986	83048096	51110	0.959737
rs5751195	rs8135801	22	42380001	42475568	95567	0.424978
rs5751195	rs2269524	22	42380001	42475703	95702	0.428474
rs5751195	rs1801311	22	42380001	42486723	106722	0.422499
rs5751195	rs6002621	22	42380001	42511002	131001	0.415547
rs5751195	rs28633410	22	42380001	42527471	147470	0.373157
rs6002621	rs28633410	22	42511002	42527471	16469	0.888576
rs60754073	rs28594416	12	123849272	123849921	649	1
rs6992091	rs7014953	8	8168222	8168413	191	0.995928
rs6992091	rs486781	8	8168222	8639740	471518	0.339854
rs7014953	rs486781	8	8168413	8639740	471327	0.341782
rs716881	rs1352318	8	89516441	89566903	50462	0.923851
rs72748702	rs12912934	15	84915246	85114447	199201	0.990317
rs76514049	rs60754073	12	123752637	123849272	96635	1
rs76514049	rs28594416	12	123752637	123849921	97284	1
rs778593	rs3822346	5	140027216	140187322	160106	0.740637
rs78532287	rs61660810	17	19912679	20170608	257929	0.596339
rs78866909	rs4759413	12	123722833	123743436	20603	1
rs78866909	rs76514049	12	123722833	123752637	29804	1

rs78866909	rs60754073	12	123722833	123849272	126439	1
rs78866909	rs28594416	12	123722833	123849921	127088	1
rs796364	rs281759	2	200716119	200787719	71600	0.966394
rs8135801	rs2269524	22	42475568	42475703	135	0.99557
rs8135801	rs1801311	22	42475568	42486723	11155	0.982361
rs8135801	rs6002621	22	42475568	42511002	35434	0.973541
rs8135801	rs28633410	22	42475568	42527471	51903	0.863791
rs910800	rs1321	22	50278642	50297435	18793	0.976108
rs910800	rs9616378	22	50278642	50311973	33331	0.875946
rs910800	rs9616382	22	50278642	50312979	34337	0.875946
rs910800	rs7410601	22	50278642	50317187	38545	0.92912
rs9616378	rs9616382	22	50311973	50312979	1006	1
rs9616378	rs7410601	22	50311973	50317187	5214	0.934931
rs9616382	rs7410601	22	50312979	50317187	4208	0.934931

[a]QRSID = Query SNP rsID, [b]RSID = Proxy SNP rsID, [c]CHR = Chromosome, [d]POS1 =Query SNP Position, [e]POS2 = Proxy SNP Position, [f]DIST = Distance between the query SNP and the proxy SNP, [g]R2 represent the degree of LD. R2=1 indicates complete LD. Only SNP pairs with R2>0.3 were shown.

Supplementary Table 7. Gene ontology analysis revealed that the target genes of regulatory SNPs are enriched in cell adhesion and nervous system development pathways

**GO category**	**Term**	**P value**	**Adjusted P value[a]**
GOTERM_BP_DIRECT	Homophilic cell adhesion via plasma membrane adhesion molecules	5.40E-09	3.30E-06
GOTERM_BP_DIRECT	Nervous system development	1.10E-04	3.30E-02

[a]P was adjusted by the Benjamini–Hochberg procedure.

Supplementary Table 8. Overlapping functional SNPs identified by functional annotation and functional genomics

**SNP**
rs11081564
rs117178087
rs2438345
rs2675960
rs7304243
rs9373388

Supplementary Table 9. Overlapping genes identified by Gusev et al. and this study

**Gene Name**
C2orf47
CORO7
CPNE7
DOC2A
DRG2
EMB
FAM109B
GATAD2A
GLT8D1
MAPK3
MAU2
NAGA
NDUFA2
NEK4
NT5C2
PCDHA2
PRMT7
SEPT10
SLC45A1
TMEM81
TSNAXIP1
ZMAT2
ZSCAN2
C10orf32
CILP2
GNL3
IK
ITIH4
KLC1
NMB
NMRAL1
PPM1M
RERE
WDR73
YPEL3
SDCCAG8
SRA1
ANAPC7
ARL3
CYP2D6
INO80E
PSMA4
CCDC90B
MPP6

Supplementary Table 10. Primer sequences used in this study.

Primer name	Primer sequence
rs12912934-F587[a]	CCAGAACATTTCTCTATCGATAGGTACCAAGCCACAGCTTAAAAGGAC
rs12912934-R587[a]	GATGCAGATCGCAGATCTCGAGTATGTAGGTGACTTTCAGTTTGG
rs12912934-F-M-587-C>T[b]	GCATAACCACCAACTGGTGTGT
rs12912934-R-M-587-C>T[b]	GTTATGCAGCCTGGAGCTTCCA
rs16937-F-560	CCAGAACATTTCTCTATCGATAGGTACCTCAGGACATTTTTCTTCCAAAG
rs16937-R-560	GATGCAGATCGCAGATCTCGAGCGAGCCTTATCAGCACTGAC
rs16937-F-M-560-A>G	TGCGTCCTCTGCTGCCCTGTCT
rs16937-R-M-560-A>G	GGACGCAGTGAACACACACCCA
rs1801311-F-638	CCAGAACATTTCTCTATCGATAGGTACCGACCCTCTTCCCCTGAAGCAC
rs1801311-R-638	GATGCAGATCGCAGATCTCGAGGATAGCAGGACGCTGAGGGA
rs1801311-F-M-638-G>A	GAAGTAACTTGGCGGACGCCGCTCCC
rs1801311-R-M-638-G>A	GCCAAGTTACTTCTACCGCCAGCACC
rs2270363-F-469	CCAGAACATTTCTCTATCGATAGGTACCATGCAACAGGGAGATCAGATACA
rs2270363-R-469	GATGCAGATCGCAGATCTCGAGCAGCCTCCAGTCAGCGTG
rs2270363-F-M-469-A>G	CATGCTCCGCTTCCCGCCCCCG
rs2270363-R-M-469-A>G	GAGCATGTGGCTTTCCCCAGGT
rs2535629-F-524	CCAGAACATTTCTCTATCGATAGGTACCCAGCTTAGACCAACAGCGTTC
rs2535629-R-524	GATGCAGATCGCAGATCTCGAGTGTGCCACTGGTGCTGATTA
rs2535629-F-M-524-G>A	CCTACAGGATGTGGGACAGCTG
rs2535629-R-M-524-G>A	CTGTAGGAAAACAGCCTTGGGC
rs2711116-F-520	CCAGAACATTTCTCTATCGATAGGTACCACAACATGGATGGATGTCAT
rs2711116-R-520	GATGCAGATCGCAGATCTCGAGGCCACTTAATACATGCATCTA
rs2711116-F-M-520-T>C	ACCCTGCTGGTGGTTACACAGG
rs2711116-R-M-520-T>C	GCAGGGTGCAGAAACAGAATAT
rs6992091-F-635	CCAGAACATTTCTCTATCGATAGGTACCAGTCGGAGGATCACTTGAGC
rs6992091-R-635	GATGCAGATCGCAGATCTCGAGGGACTCCACTCATTGAAATAGTTC
rs6992091-F-M-635-G>A	CCCAGCTGCAGGGCACAGTCGG
rs6992091-R-M-635-G>A	AGCTGGGGTTGGCAGACACCTC
rs7012106-F-383	CCAGAACATTTCTCTATCGATAGGTACCATCACTCGCCCTGCTCGCCA
rs7012106-R-383	GATGCAGATCGCAGATCTCGAGTTGGCGCGGTCCCACTTG
rs7012106-F-M-383-C>G	CCCCCTGGGCTCTTTTCGGGCCGGCG
rs7012106-R-M-383-C>G	AGAGCCCAGGGGGAAGGGGCCCGGGA
rs7014953-F-502	CCAGAACATTTCTCTATCGATAGGTACC AACGCTGTGATCAAATGTGT
rs7014953-R-502	GATGCAGATCGCAGATCTCGAGTGCCTATGGCCGGTTCTC
rs7014953-F-M-502-A>C	AACAGCCCATGACTCATGTTGTACTC
rs7014953-R-M-502-A>C	GTCATGGGCTGTTTCAAGTACCCAAC
rs7709645-F-646	CCAGAACATTTCTCTATCGATAGGTACCGGTAAACACAGACAAGTAACCAGAT
rs7709645-R-646	GATGCAGATCGCAGATCTCGAGTCCCGACTTCAGGTGATCC
rs7709645-F-M-646-G>C	GATGTGCTTTTGCTTTAAATCAGGAT
rs7709645-R-M-646-G>C	GCAAAAGCACATCTGTTTATGAGTAA
rs11655813-F-593	CCAGAACATTTCTCTATCGATAGGTACCTGAAGTCTATTAGGGTGAAGCC
rs11655813-R-593	GATGCAGATCGCAGATCTCGAGGCCATCTGAGTTTCCACCTT
rs11655813-F-593-C>T	AGATTAATTCTATGTCTGGCCTGGA
rs11655813-R-593-C>T	TCCAGGCCAGACATAGAATTAATCT
rs9908888-F-484	CCAGAACATTTCTCTATCGATAGGTACCAGTATAGCAAGAGAACGTCATCG

rs9908888-R-484	GATGCAGATCGCAGATCTCGAGGACATAATCACTCCACAATACTGC
rs9908888-F-484-G>C	CAGTTAAAAAATCTTCTTGCTTCTC
rs9908888-R-484-G>C	GAGAAGCAAGAAGATTTTTTAACTG
rs17821573-F-533	CCAGAACATTTCTCTATCGATAGGTACCTGCCTGTGGTCCCATCTAC
rs17821573-R-533	GATGCAGATCGCAGATCTCGAGGACCTCAAATGATCCACCCAC
rs17821573-F-533-T>C	ACTGAAATAAAACGTAAATGTTGGA
rs17821573-R-533-T>C	TCCAACATTTACGTTTTATTTCAGT
rs28676999-F-473	CCAGAACATTTCTCTATCGATAGGTACCGAGTGTTCAAAGCCATTGG
rs28676999-R-473	GATGCAGATCGCAGATCTCGAGCACACCATCTCCCTCAGTC
rs28676999-F-473-C>T	GAAGTGAAAGGCTCTTGGGAATGAA
rs28676999-R-473-C>T	TTCATTCCCAAGAGCCTTTCACTTC
rs301791-F-514	CCAGAACATTTCTCTATCGATAGGTACCGGTTGAAGCAGCCTGCATAA
rs301791-R-514	GATGCAGATCGCAGATCTCGAGAAGTAGGCATTTGTGCGTCC
rs301791-F-514-A>T	CATTTCTACTGTTTCACATCCCACA
rs301791-R-514-A>T	TGTGGGATGTGAAACAGTAGAAATG
rs37718-F-451	CCAGAACATTTCTCTATCGATAGGTACCCTAACCATGGCTTAGAAGGC
rs37718-R-451	GATGCAGATCGCAGATCTCGAGCACCAGACTAGTATGCTTCAGG
rs37718-F-451-C>T	CTGTCATGTTTCAAACTCCTTCGGTGTGAGATG
rs37718-R-451-C>T	CATCTCACACCGAAGGAGTTTGAAACATGACAG
rs7304782-F-511	CCAGAACATTTCTCTATCGATAGGTACCCCAGGACTTACTTCATTGAAGATG
rs7304782-R-511	GATGCAGATCGCAGATCTCGAGACCTGCGTTCAATGTCTCAAT
rs7304782-F-511-A>G	TTTCACAGACCAGAATTATGATCCC
rs7304782-R-511A>G	GGGATCATAATTCTGGTCTGTGAAA
rs4702-F-334	CCAGAACATTTCTCTATCGATAGGTACCAAAGCACTGACCTGTCATGC
rs4702-R-334	GATGCAGATCGCAGATCTCGAGGTTTGCATAGTCTGCACGTTTA
rs4702-F-334-A>G	GTTTTGTAAGATACTGGGTTGGTGC
rs4702-R-334-G>A	GCACCAACCCAGTATCTTACAAAAC
rs696520-F-669	CCAGAACATTTCTCTATCGATAGGTACCCGCAATTAGGATGAGCTGCT
rs696520-R-669	GATGCAGATCGCAGATCTCGAGCGAGAGAGAGCTGAGCTAAGG
rs696520-F-669-C>T	GGATAAGTCCAATAGTAGGACACAA
rs696520-R-669-C>T	TTGTGTCCTACTATTGGACTTATCC
rs7752421-F-552	CCAGAACATTTCTCTATCGATAGGTACCTTACCAACCTGACTGGGACA
rs7752421-R-552	GATGCAGATCGCAGATCTCGAGGTCATCTGAGAGGACCTCACAT
rs7752421-F-552-A>T	GAGAAGACTGAATGTGAAGATGCAC
rs7752421-R-552-A>T	GTGCATCTTCACATTCAGTCTTCTC

[a]This pair of primers were used to amplify the DNA fragments containing the tested SNP. [b]This pair of primers were used for PCR-mediated site mutation (i.e., to obtain the sequence containing the alternative allele of the tested SNP).

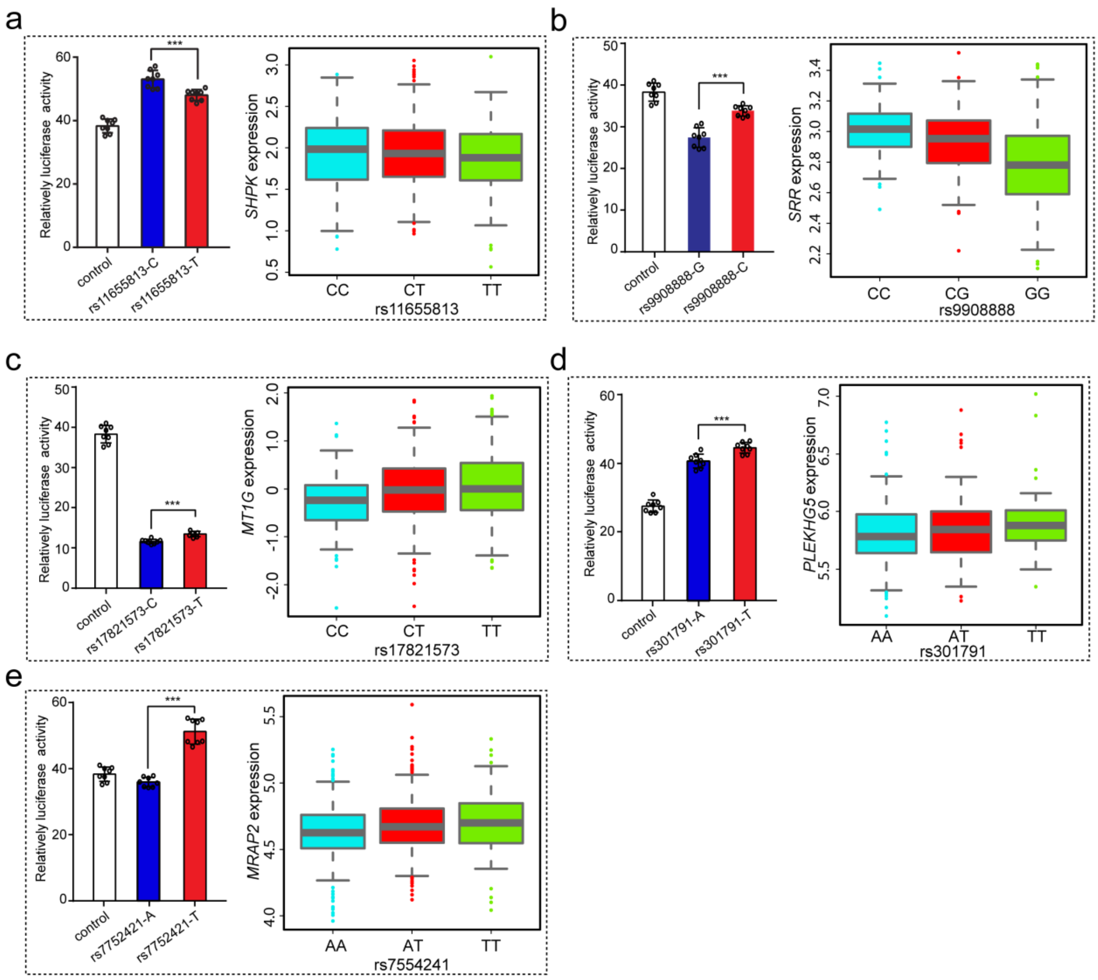

Supplementary Figure 1. Comparison of the allelic effects on luciferase activity observed in reporter gene assays with the estimated eQTL effects of the corresponding alleles in CMC dataset. Seven SNPs (rs11655813, rs9908888, rs17821573, rs301791, rs37718, rs7304782, rs7752421) in Fig.2b showed significant allelic effects in reporter gene assays. Among these 7 significant SNPs, 5 SNPs (rs11655813, rs9908888, rs17821573, rs301791 and rs7752421) were significantly associated (FDR<0.01) with gene expression in CMC dataset. For these 5 significant eQTL SNPs, we found that the allelic effects on gene expression observed in reporter gene assays are consistent with the estimated eQTL effects of the corresponding alleles in CMC dataset. Of note, in some cases, the same SNP is associated with the expression of several genes. Only the gene whose expression in different genotypic individuals was consistent with the allelic effects observed in reporter gene assays was showed here. The left part of each panel is reporter gene assay result and the right part is eQTL result.

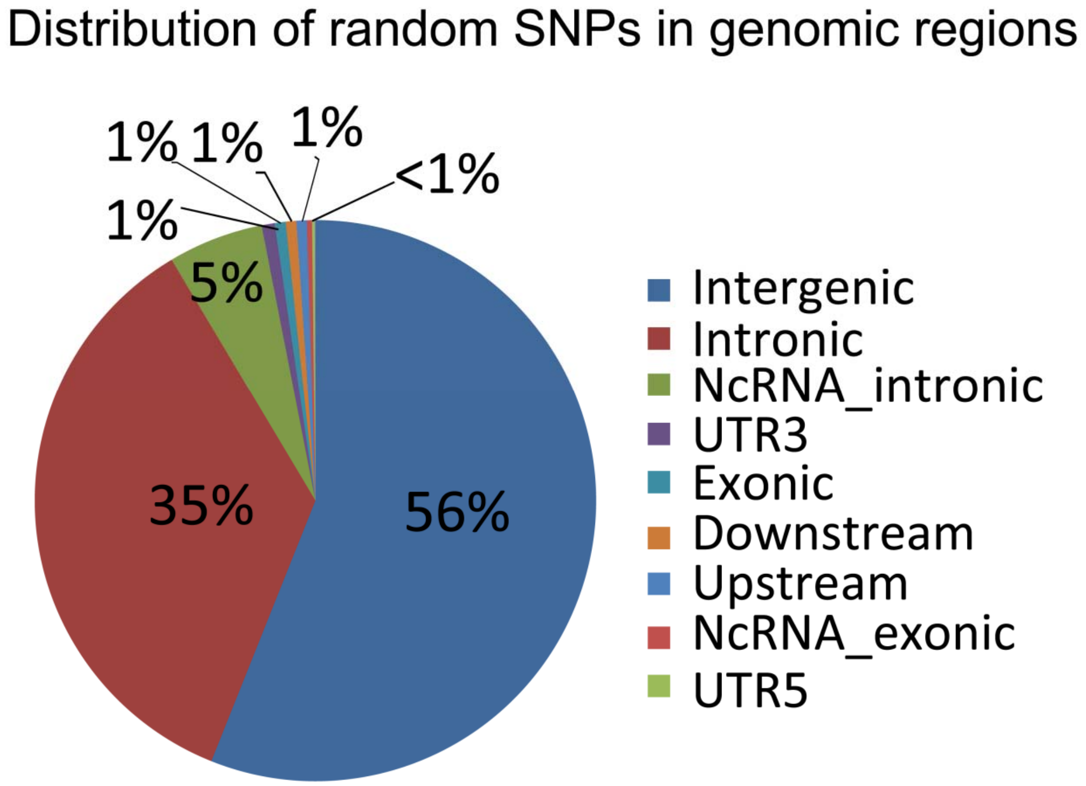

Supplementary Figure 2. Distribution of random SNPs in the human genome. Most of the random SNPs were located in intergenic and intronic regions (56% random SNP were located in intergenic regions and 35% random SNPs were located in intronic regions).

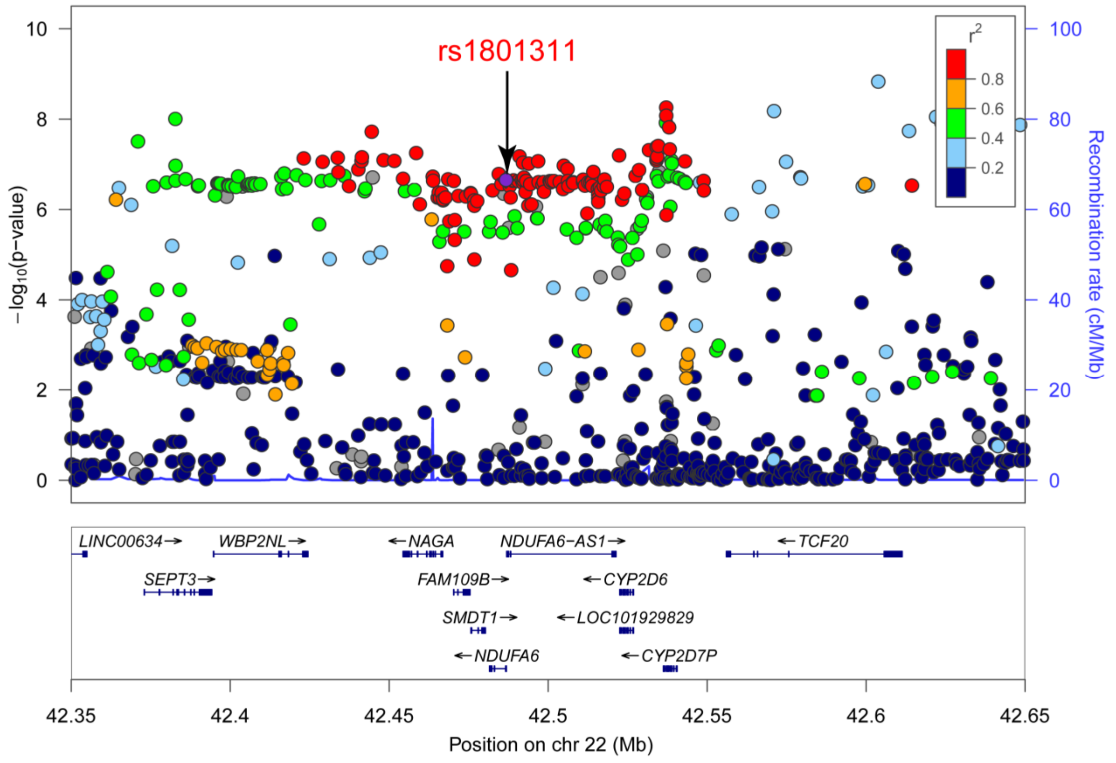

Supplementary Figure 3. SNP rs1801311 is located in 22q13.2, a schizophrenia risk locus identified by genome-wide association study. Due to linkage disequilibrium, this locus harbors multiple SNPs that show similar association significance with schizophrenia. The TF binding-disrupting SNP (i.e., rs1801311) is highlighted by black arrow.

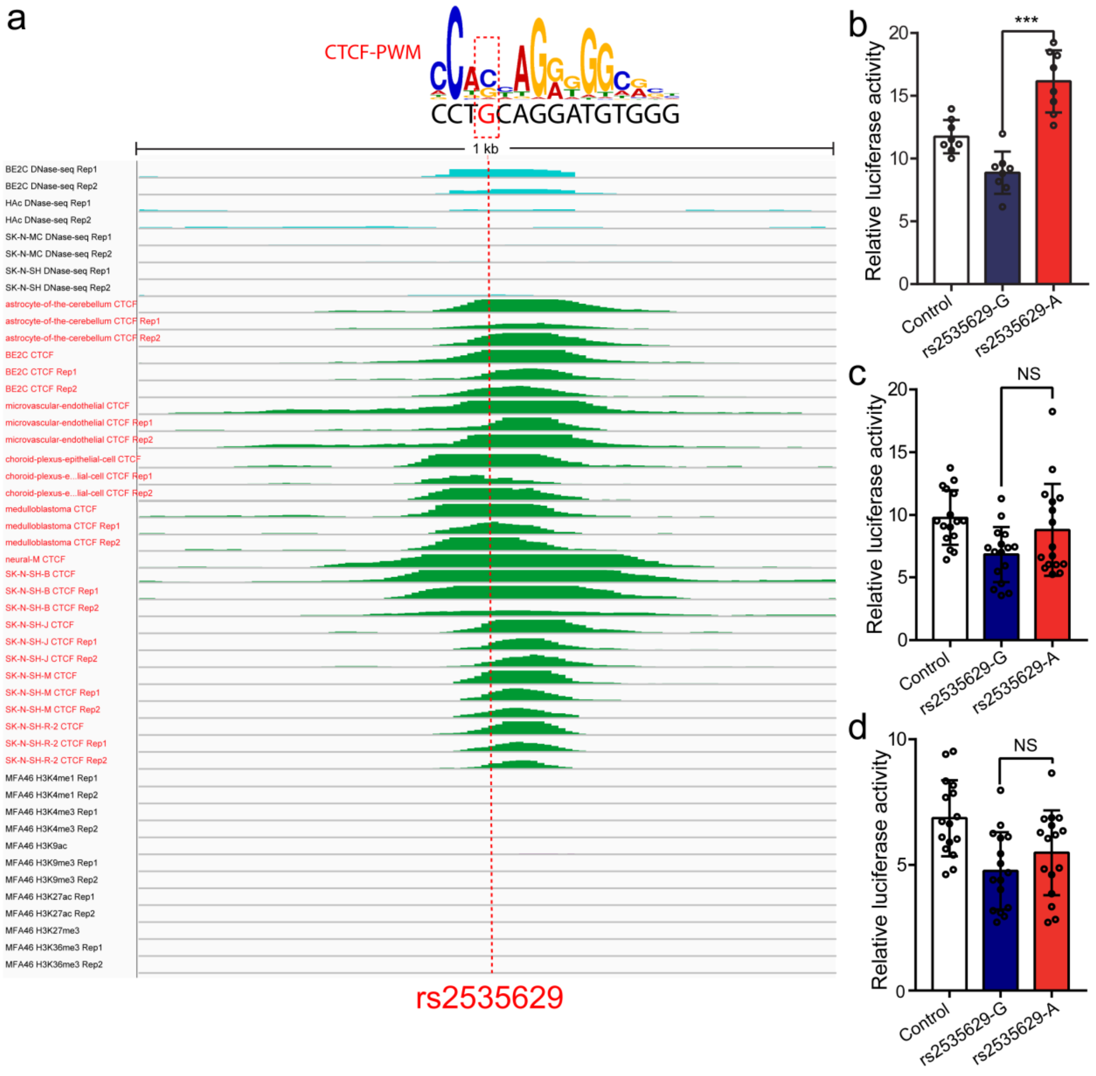

Supplementary Figure 4. SNP rs2535629 disputes an occupied CTCF binding site. (a) SNP rs2535629 is located in the CTCF binding motif and disrupts occupied CTFF binding site in human brain tissues or neuronal cell lines. PWMs of CTCF and genomic sequence containing SNP rs2535629 were shown (rs2535629 was marked with red dotted box). Genomic region (1 kb) surrounding SNP rs2535629 was shown with DNase-Seq signal (light blue) and ChIP-Seq signal (green). **(b-d)** Reporter gene assays showed that the allelic differences at the rs2535629 influenced the luciferase activity significantly in HEK293 cells **(b),** but not in SK-N-SH **(c)** and SH-SY5Y cells **(d)**. Data represent mean±SD. n=8 for each group in **(b)** (HEK293 cells). n=16 for each group in **(c)** (SK-N-SH cells) and **(d)** (SH-SY5Y cells). NS, not significant. ****P*<0.001, two-tailed Student's *t* test. Source data are provided as a Source Data file.

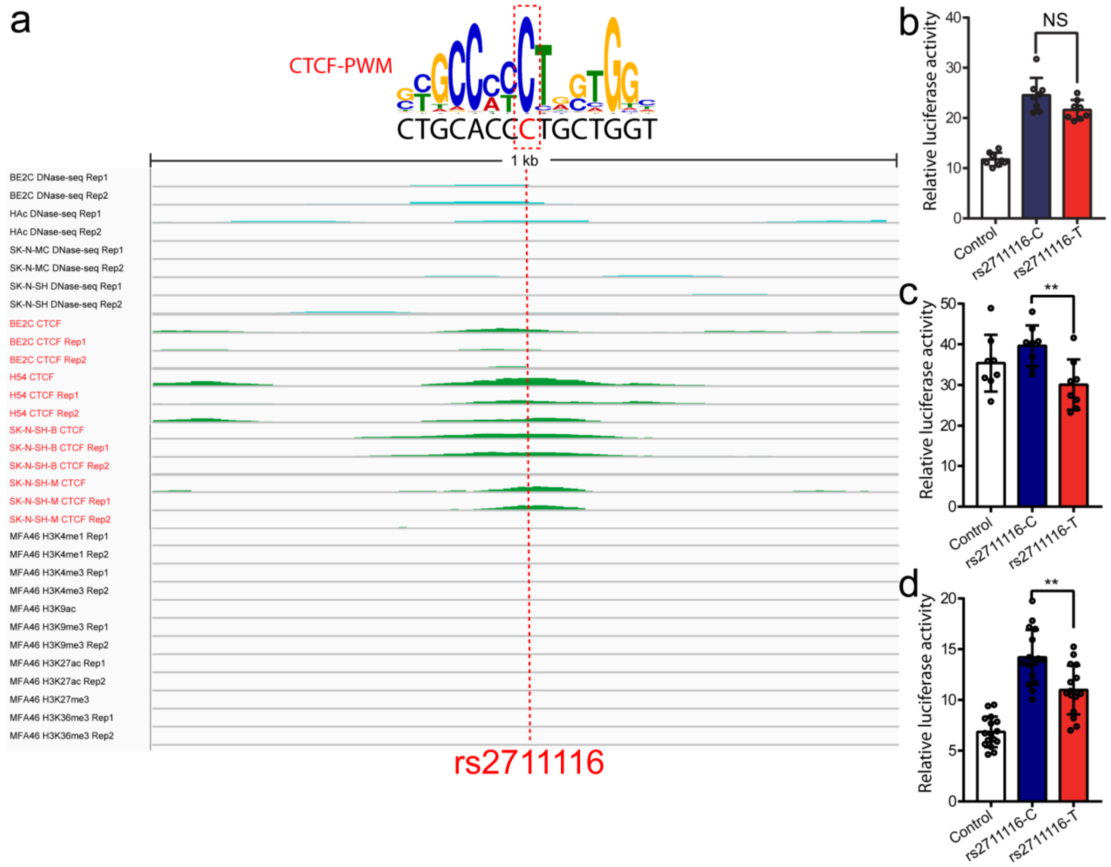

Supplementary Figure 5. SNP rs2711116 disputes an occupied CTCF binding site. (a) SNP rs2711116 is located in the CTCF binding motif and disrupts occupied CTFF binding site in human brain tissues or neuronal cell lines. PWMs of CTCF and genomic sequence containing SNP rs2711116 were shown (rs2711116 was marked with red dotted box). Genomic region (1 kb) surrounding SNP rs2711116 was shown with DNase-Seq signal (light blue) and ChIP-Seq signal (green). **(b-d)** Reporter gene assays showed that the allelic differences at the rs2711116 influenced the luciferase activity significantly in SK-N-SH cells **(c)** and SH-SY5Y cells **(d),** but not in HEK293 cells **(b)**. Data represent mean±SD. n=8 for each group in **(b)** (HEK293 cells) and **(c)** (SK-N-SH cells). n=16 for each group in **(d)** (SH-SY5Y cells). NS, not significant. ***P*<0.01, two-tailed Student's *t* test. Source data are provided as a Source Data file.

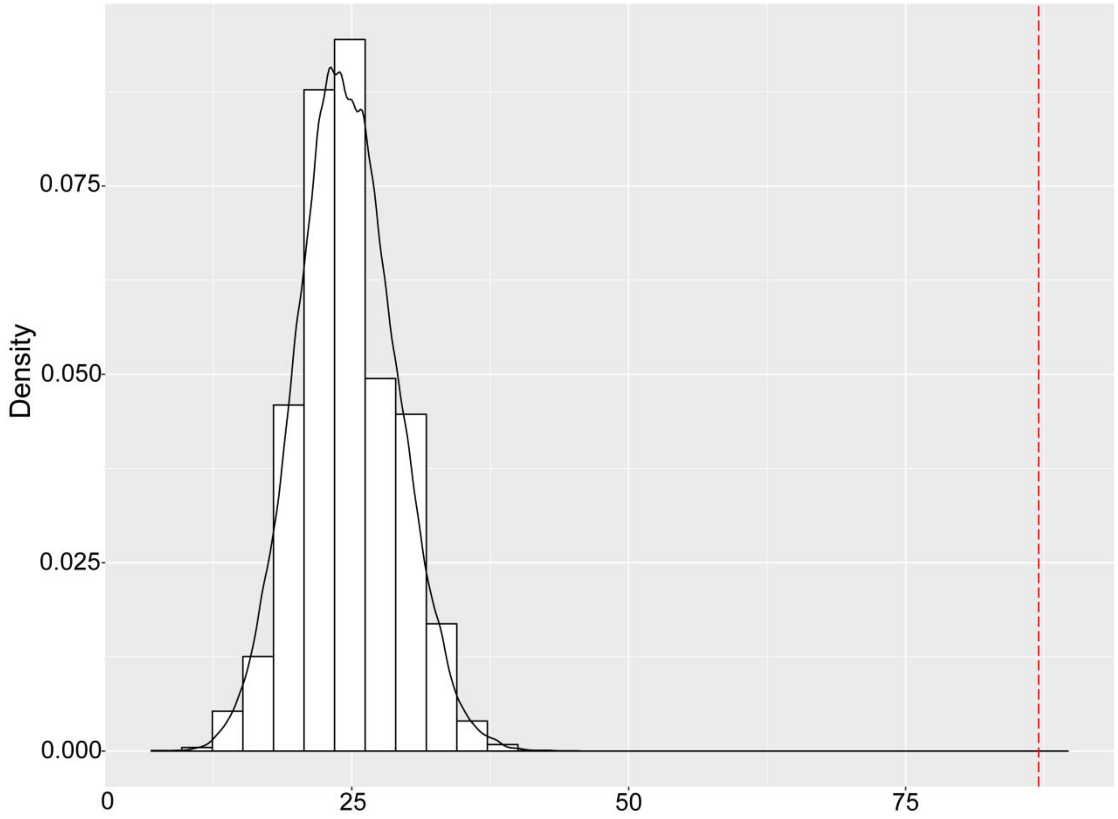

Supplementary Figure 6. The identified regulatory SNPs showed significant association with gene expression (Z=14.23, *P*=3.09×10^{-46}) compared with random SNPs. The histogram reflects the distribution of the number of random SNPs that showed significant association with gene expression in CMC dataset. The red dashed line indicated the number of observed significant SNPs (n=87) in CMC dataset.

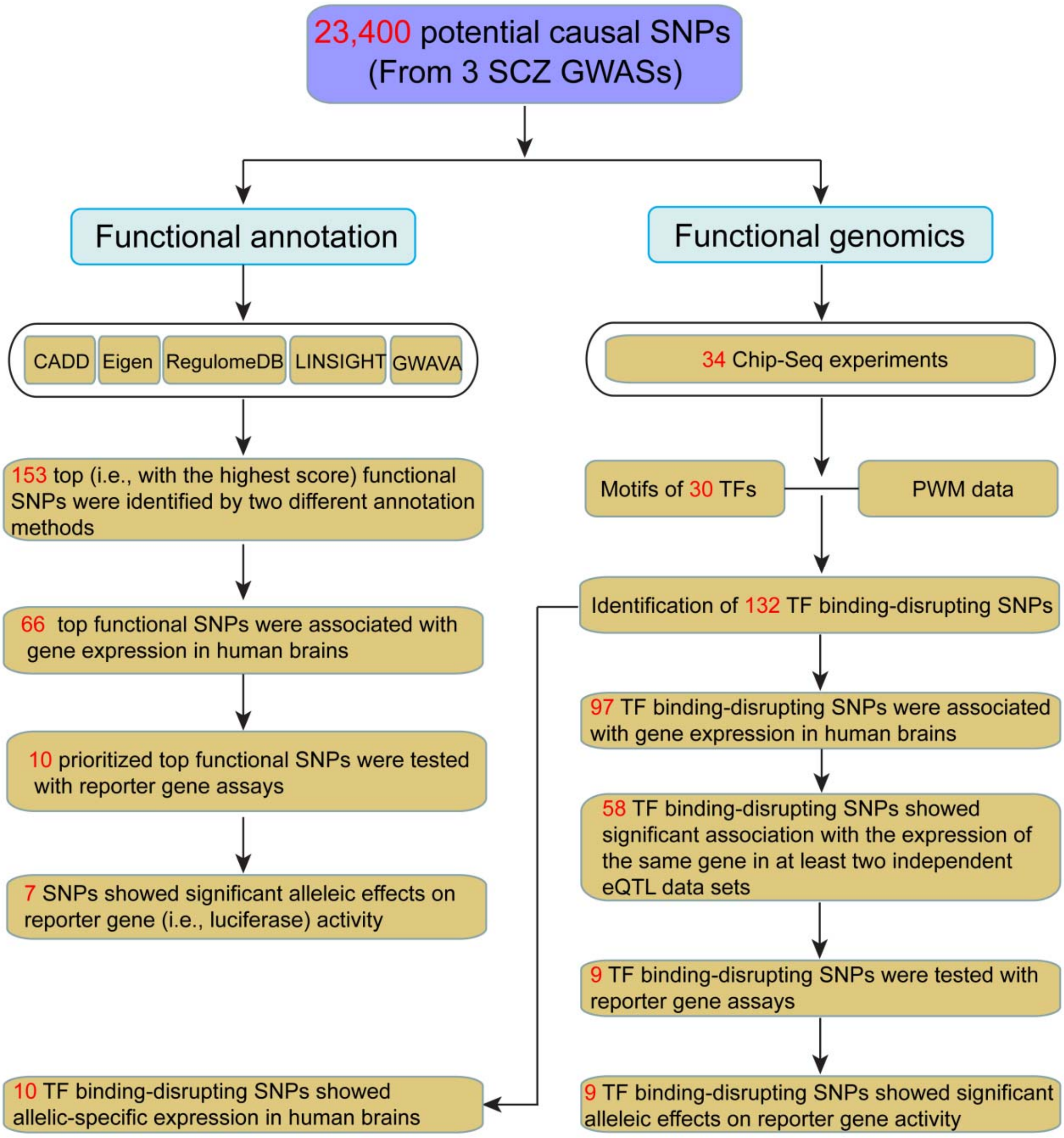

Supplementary Figure 7. Summary of the functional SNPs identified at each stage. Functional annotation approaches prioritized 153 top functional SNPs. Among the 153 top functional SNPs, 66 SNPs were associated with gene expression in human brain tissues and 7 SNPs exhibited allelic effects on reporter gene activity. Functional genomics identified 132 TF binding-disrupting SNPs. Among these 132 TF binding-disrupting SNPs, 97 were associated with gene expression in human brain tissues and 58 showed significant association with the same gene in at least two independent brain eQTL datasets. We tested 9 TF binding-disrupting SNPs with reporter gene assays. Of note, all of the tested SNPs showed significant allelic effects on reporter gene (i.e., luciferase) activity. In addition, 10 TF binding-disrupting SNPs showed allelic-specific expression in human brain tissues.

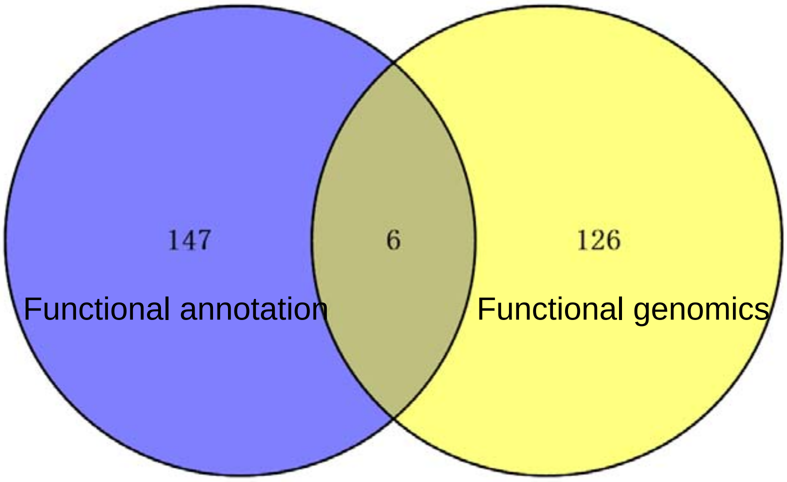

Supplementary Figure 8. Venn diagram shows the overlapping SNPs identified by functional annotation and functional genomics approaches. The 6 overlapping SNPs are rs11081564, rs117178087, rs2438345, rs2675960, rs7304243 and rs9373388.

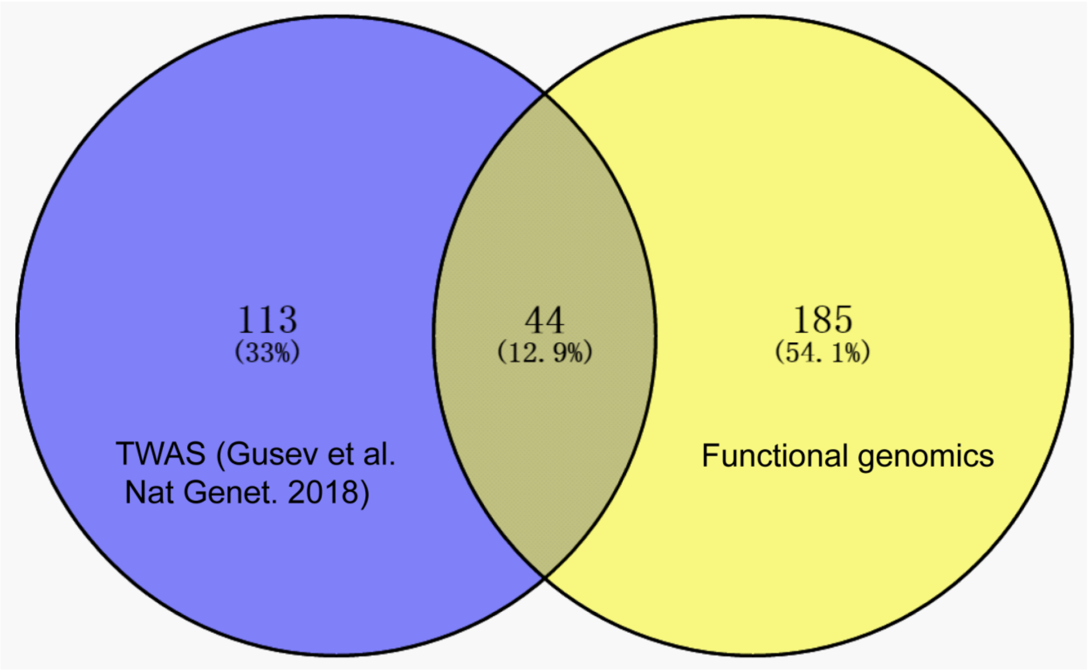

Supplementary Figure 9. Venn diagram shows the overlapping genes identified by Gusev *et al*. (Gusev et al., Nat Genet. 2018) and functional genomics. Of note, 44 overlapping genes were identified by Gusev *et al*. and this study.

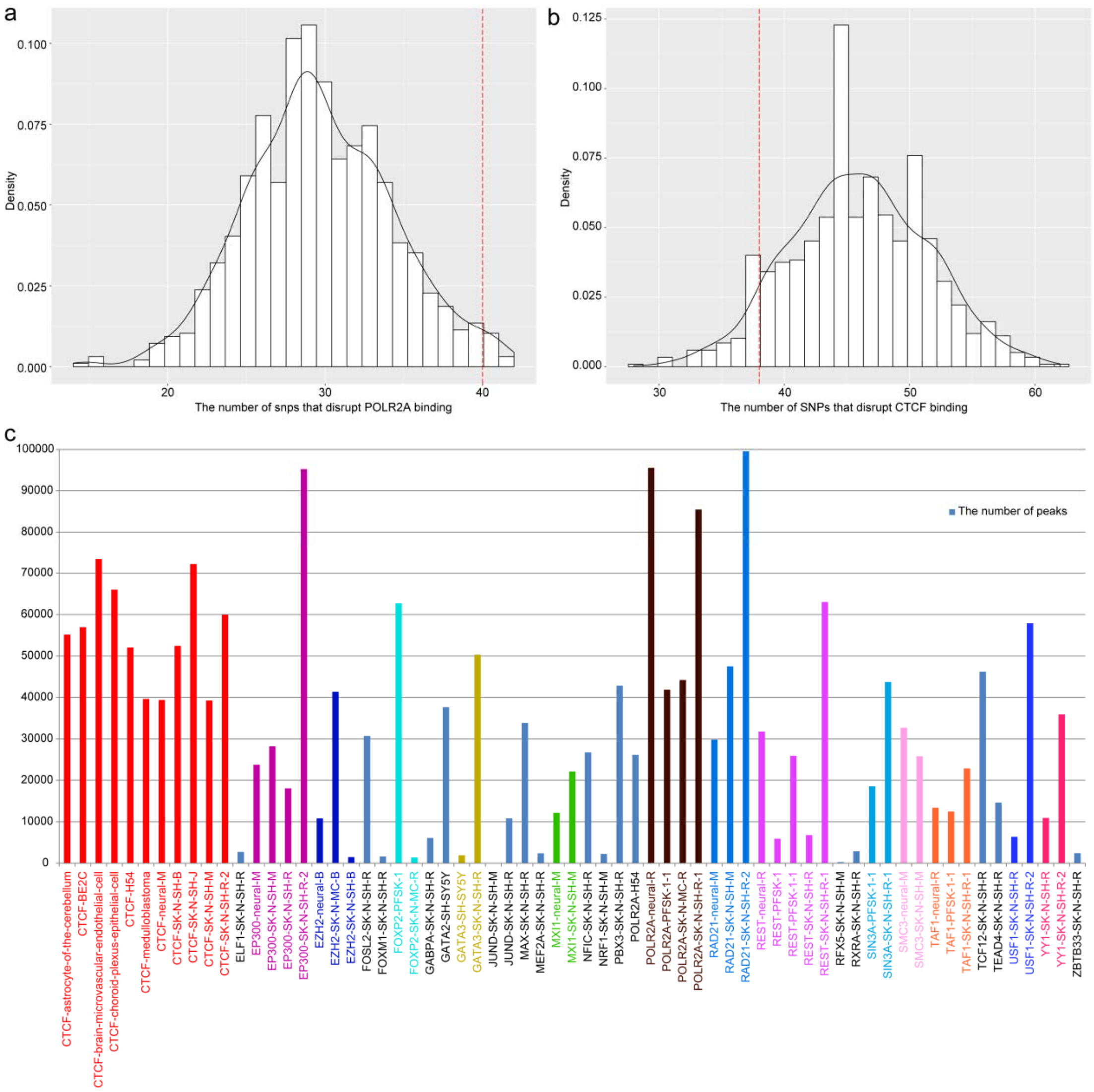

Supplementary Figure 10. The number of random SNPs that disrupt POLR2A and CTCF binding. (**a**) The distribution of random SNPs that disrupted POLR2A binding. The number of POLR2A binding-disrupting SNPs observed in this study were significantly higher than random SNPs (*P*=0.013, 1,000 simulations). (**b**) The distribution of random SNPs that disrupted CTCF binding. The number of CTCF binding-disrupting SNPs observed in this study were not significantly different from random SNPs (*P*>0.05, 1,000 simulations). (**c**) The number of motifs (ChIP-Seq peaks) of each TF included in this study. The detail information about the ChIP-Seq peaks from different neuronal cells or brain tissues can be found in Supplementary Table 6.

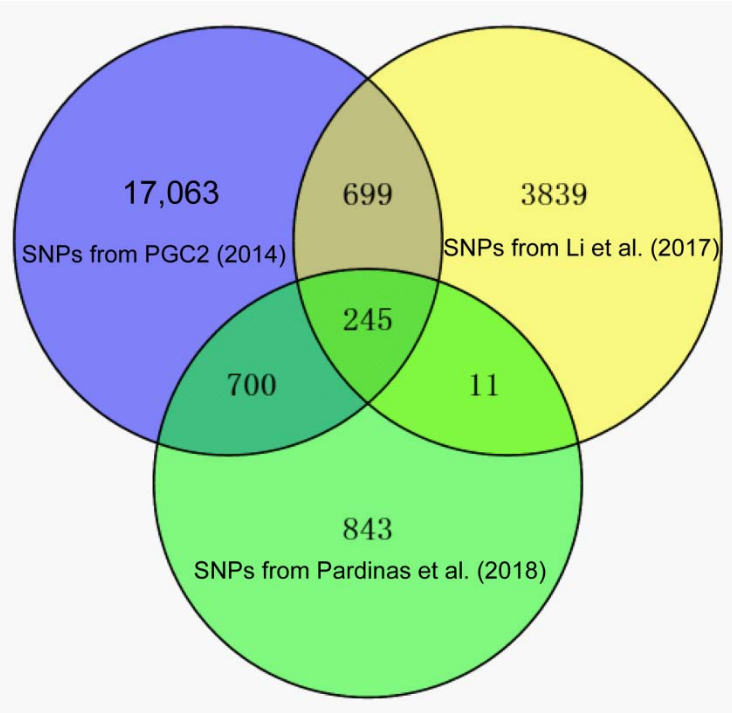

Supplementary Figure 11. Venn diagram shows the overlapping SNPs of the three schizophrenia GWAS.

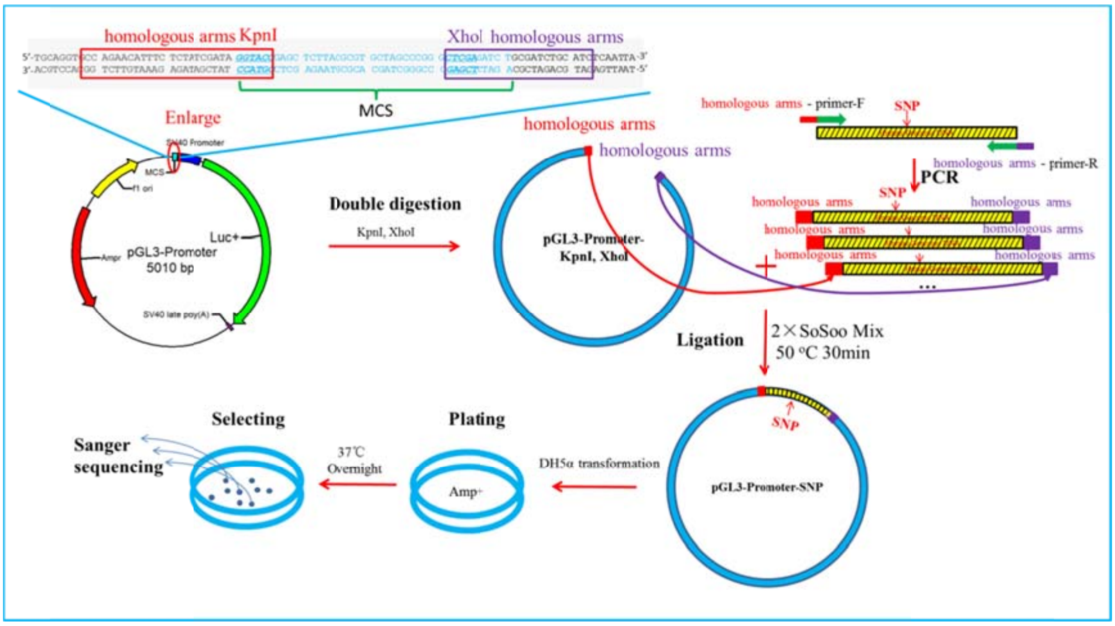

Supplementary Figure 12. Construction of reporter gene vector. PGL3-promoter vectors were digested by KpnI and XhoI, and the enzyme-digested products were purified with DNA Purification Kit. The DNA fragments (the test SNP was located in the fragments) were amplified using PCR primers tagged with homologous arms (the sequence of homologous arms are identical with the pGL3-promoter vector DNA sequence). The PCR procucts were purified and ligated to the PGL3-promoter vector using 2×SoSoo Mix. The recombinant plasmids were used to transforme DH5α cells, and single colony were picked for Sanger sequencing.

References

1. Owen, M.J., O'Donovan, M.C., Thapar, A. & Craddock, N. Neurodevelopmental hypothesis of schizophrenia. *Br J Psychiatry* **198**, 173-175 (2011).
2. Fatemi, S.H. & Folsom, T.D. The neurodevelopmental hypothesis of schizophrenia, revisited. *Schizophr Bull* **35**, 528-548 (2009).
3. Rapoport, J.L., Giedd, J.N. & Gogtay, N. Neurodevelopmental model of schizophrenia: update 2012. *Mol Psychiatry* **17**, 1228-1238 (2012).
4. Mao, Y. et al. Disrupted in schizophrenia 1 regulates neuronal progenitor proliferation via modulation of GSK3beta/beta-catenin signaling. *Cell* **136**, 1017-1031 (2009).
5. Yang, C.P. et al. Comprehensive integrative analyses identify GLT8D1 and CSNK2B as schizophrenia risk genes. *Nat Commun* **9**, 838 (2018).
6. Walsh, T. et al. Rare structural variants disrupt multiple genes in neurodevelopmental pathways in schizophrenia. *Science* **320**, 539-543 (2008).
7. O'Dushlaine, C. et al. Molecular pathways involved in neuronal cell adhesion and membrane scaffolding contribute to schizophrenia and bipolar disorder susceptibility. *Mol Psychiatry* **16**, 286-292 (2011).
8. Zhang, Z. et al. Evidence for Association of Cell Adhesion Molecules Pathway and NLGN1 Polymorphisms with Schizophrenia in Chinese Han Population. *PLoS One* **10**, e0144719 (2015).
9. Won, H. et al. Chromosome conformation elucidates regulatory relationships in developing human brain. *Nature* **538**, 523-527 (2016).
10. Gilman, S.R. et al. Diverse types of genetic variation converge on functional gene networks involved in schizophrenia. *Nat Neurosci* **15**, 1723-1728 (2012).
11. Schizophrenia Working Group of the Psychiatric Genomics Consortium*. Biological insights from 108 schizophrenia-associated genetic loci. *Nature* **511**, 421-427 (2014).
12. Pers, T.H. et al. Comprehensive analysis of schizophrenia-associated loci highlights ion channel pathways and biologically plausible candidate causal genes. *Hum Mol Genet* **25**, 1247-1254 (2016).
13. Xu, B. et al. De novo gene mutations highlight patterns of genetic and neural complexity in schizophrenia. *Nat Genet* **44**, 1365-1369 (2012).
14. Gulsuner, S. et al. Spatial and temporal mapping of de novo mutations in schizophrenia to a fetal prefrontal cortical network. *Cell* **154**, 518-29 (2013).
15. Phillips, J.E. & Corces, V.G. CTCF: master weaver of the genome. *Cell* **137**, 1194-1211 (2009).
16. Hou, C., Zhao, H., Tanimoto, K. & Dean, A. CTCF-dependent enhancer-blocking by alternative chromatin loop formation. *Proc Natl Acad Sci U S A* **105**, 20398-20403 (2008).
17. Lee, B.K. & Iyer, V.R. Genome-wide studies of CCCTC-binding factor (CTCF) and cohesin provide insight into chromatin structure and regulation. *J Biol Chem* **287**, 30906-30913 (2012).
18. Juraeva, D. et al. Integrated pathway-based approach identifies association between genomic regions at CTCF and CACNB2 and schizophrenia. *PLoS Genet* **10**, e1004345 (2014).
19. Baruch, K. et al. Association between golli-MBP and schizophrenia in the Jewish Ashkenazi population: are regulatory regions involved? *Int J Neuropsychopharmacol* **12**, 885-94 (2009).
20. Pardinas, A.F. et al. Common schizophrenia alleles are enriched in mutation-intolerant genes

and in regions under strong background selection. *Nat Genet* **50**, 381-389 (2018).
21. Li, Z. et al. Genome-wide association analysis identifies 30 new susceptibility loci for schizophrenia. *Nat Genet* **49**, 1576-1583 (2017).
