# Supplementary material for: Functional genomics reveal gene regulatory mechanisms underlying schizophrenia risk
Source: Nat Commun. 2019 Feb 8;10:670. doi: 10.1038/s41467-019-08666-4 (PMC6368563; doi:10.1038/s41467-019-08666-4)
Supplement: Supplementary file 3 — Description of Additional Supplementary Files [file 41467_2019_8666_MOESM3_ESM.pdf]

Description of Additional Supplementary Files

Supplementary Data 1: The most possible functional SNPs identified by different annotation approaches (credible causal SNPs were from PGC2).

Supplementary Data 2: The prioritized top functional SNPs are associated with gene expression in human brains.

Supplementary Data 3: TF binding-disrupting SCZ risk SNPs.

Supplementary Data 4: The association significance between the identified TF binding-disrupting SNPs and gene expression in human brain.

Supplementary Data 5: The associations of the regulatory SNPs with all genes in close physical proximity (e.g., +/- 2Mb) in CMC dataset.

Supplementary Data 6: Brain expression quantitative trait locus (eQTL) analysis results (box plots) (only TF binding-disrupting SNPs listed in Table 1 and Table 2 were showed).

Supplementary Data 7: SNPs associated with CTCF expression in human brain tissue (nucleus accumbens (basal ganglia)) (data were from GTEx).
