# Supplementary material for: Functional genomics reveal gene regulatory mechanisms underlying schizophrenia risk
Source: Nat Commun. 2019 Feb 8;10:670. doi: 10.1038/s41467-019-08666-4 (PMC6368563; doi:10.1038/s41467-019-08666-4)

**Supplementary Data 6: Brain expression quantitative trait locus (eQTL) analysis results (box plots) (only TF binding-disrupting SNPs listed in Table 1 and Table 2 were showed).**


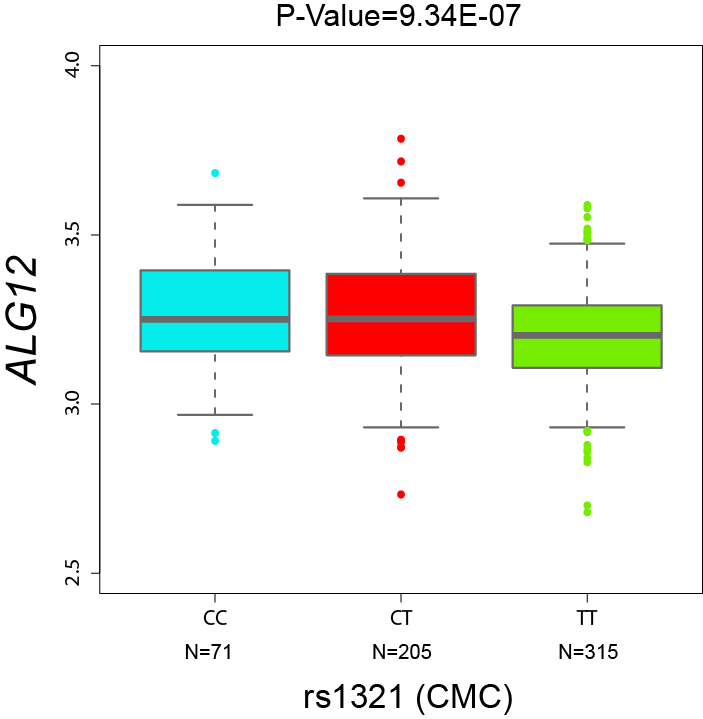


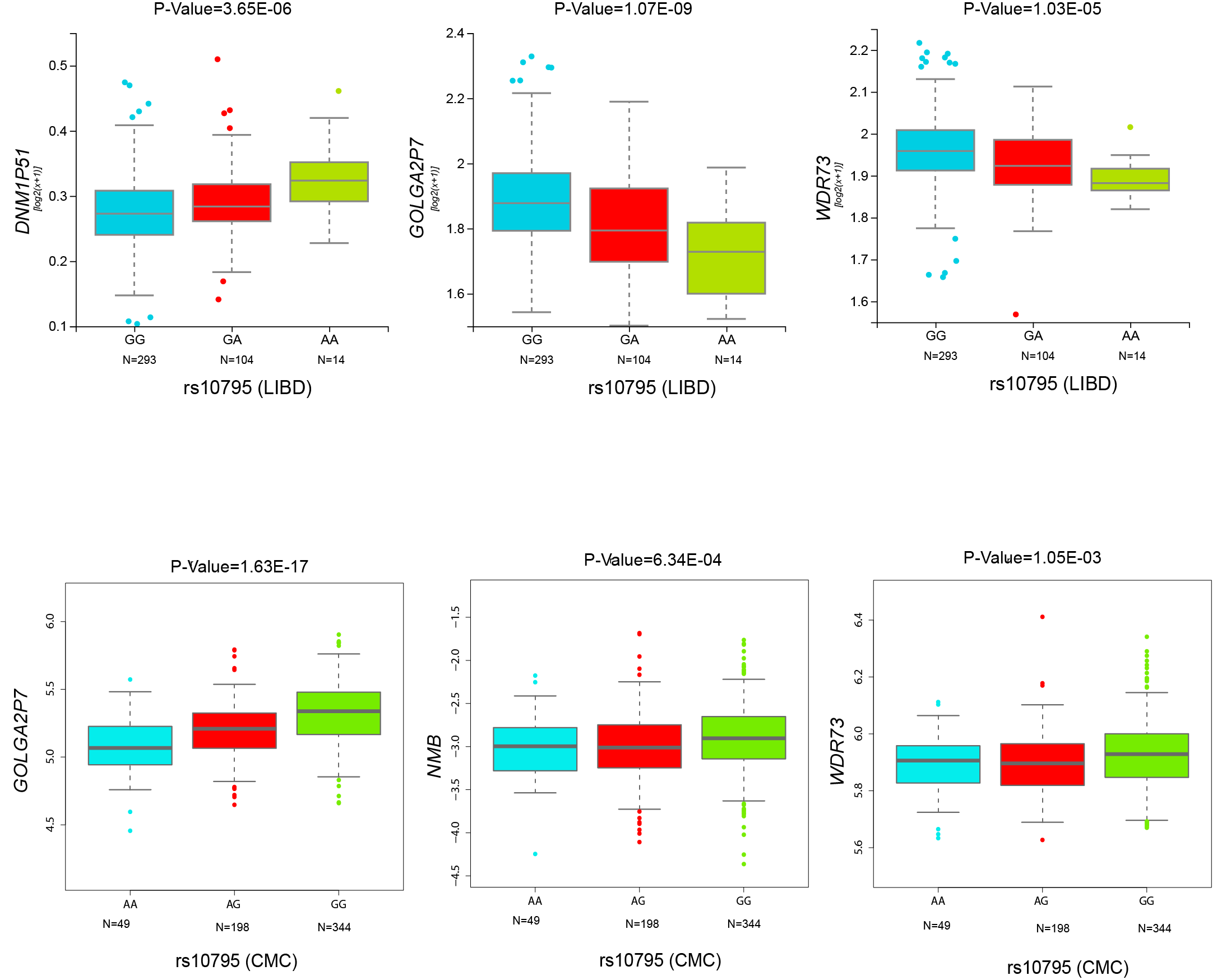

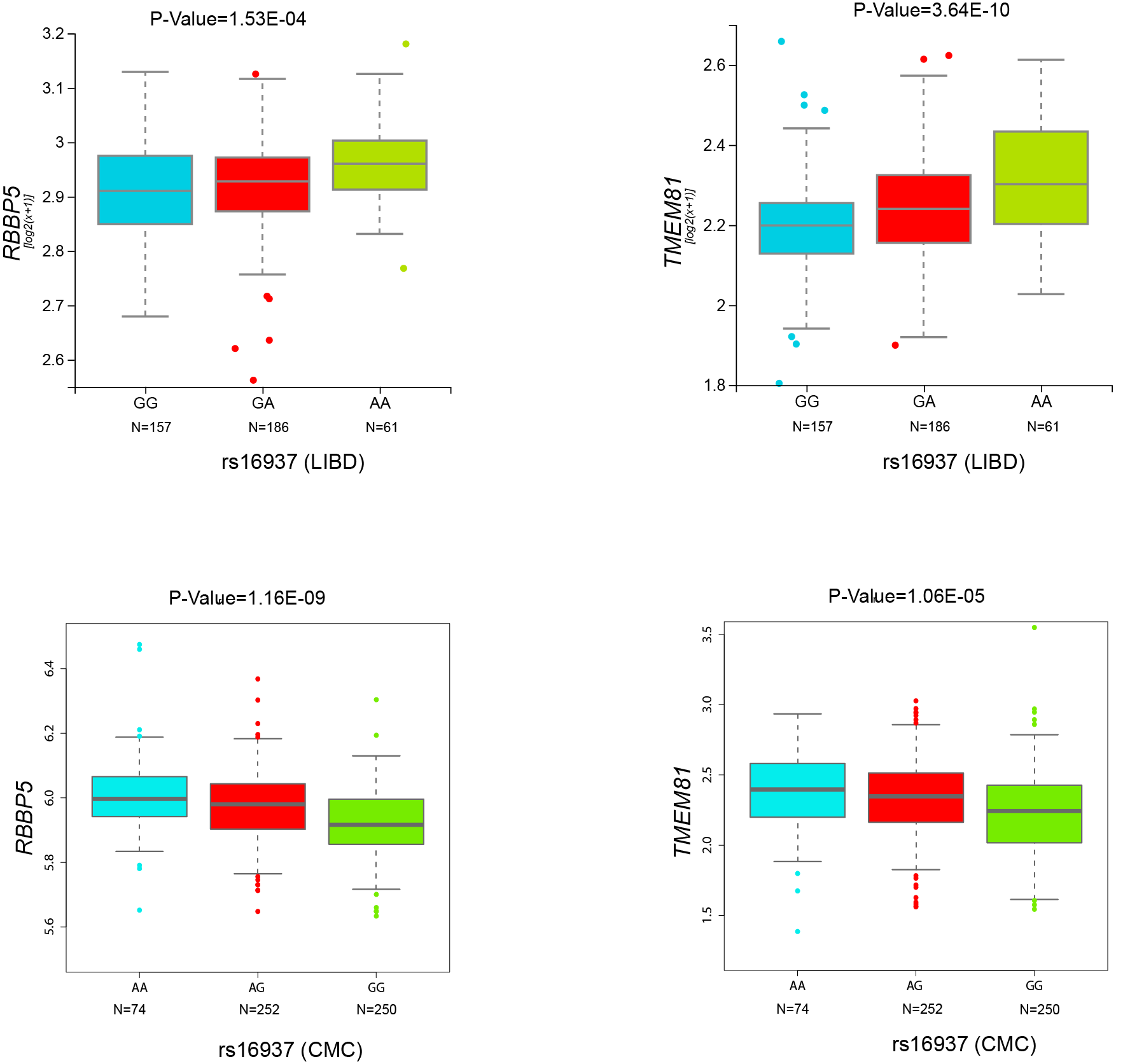

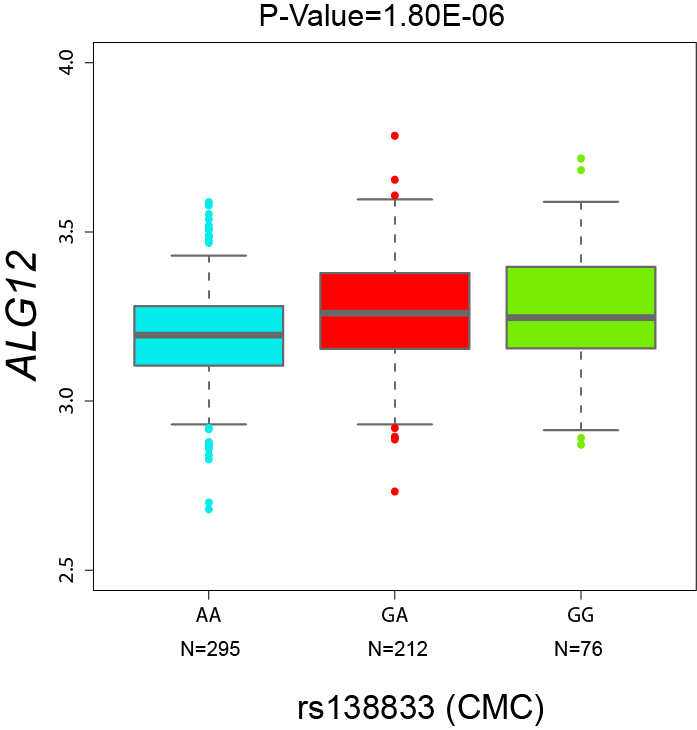

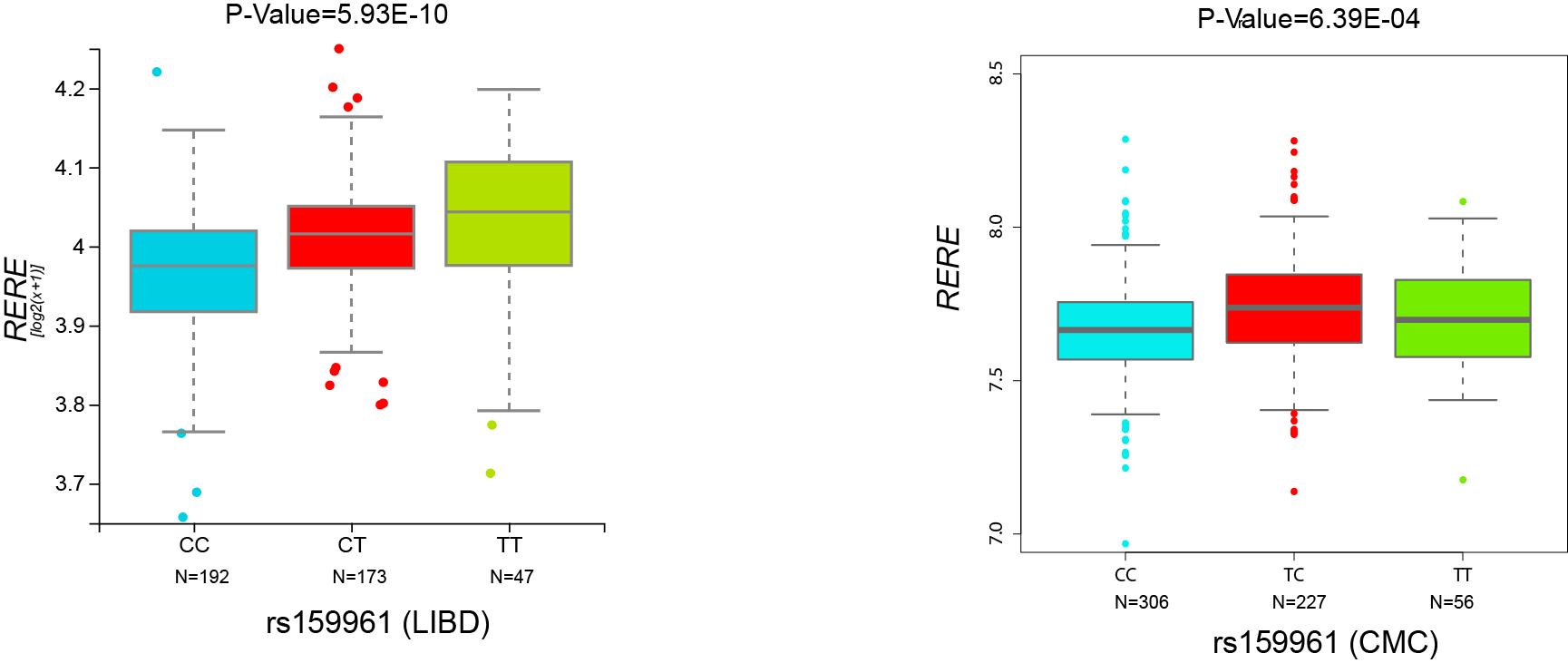

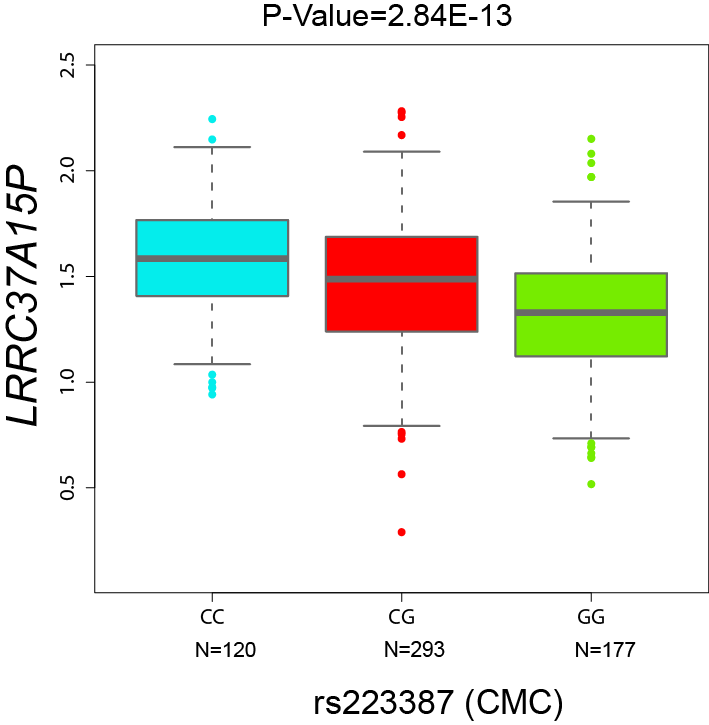

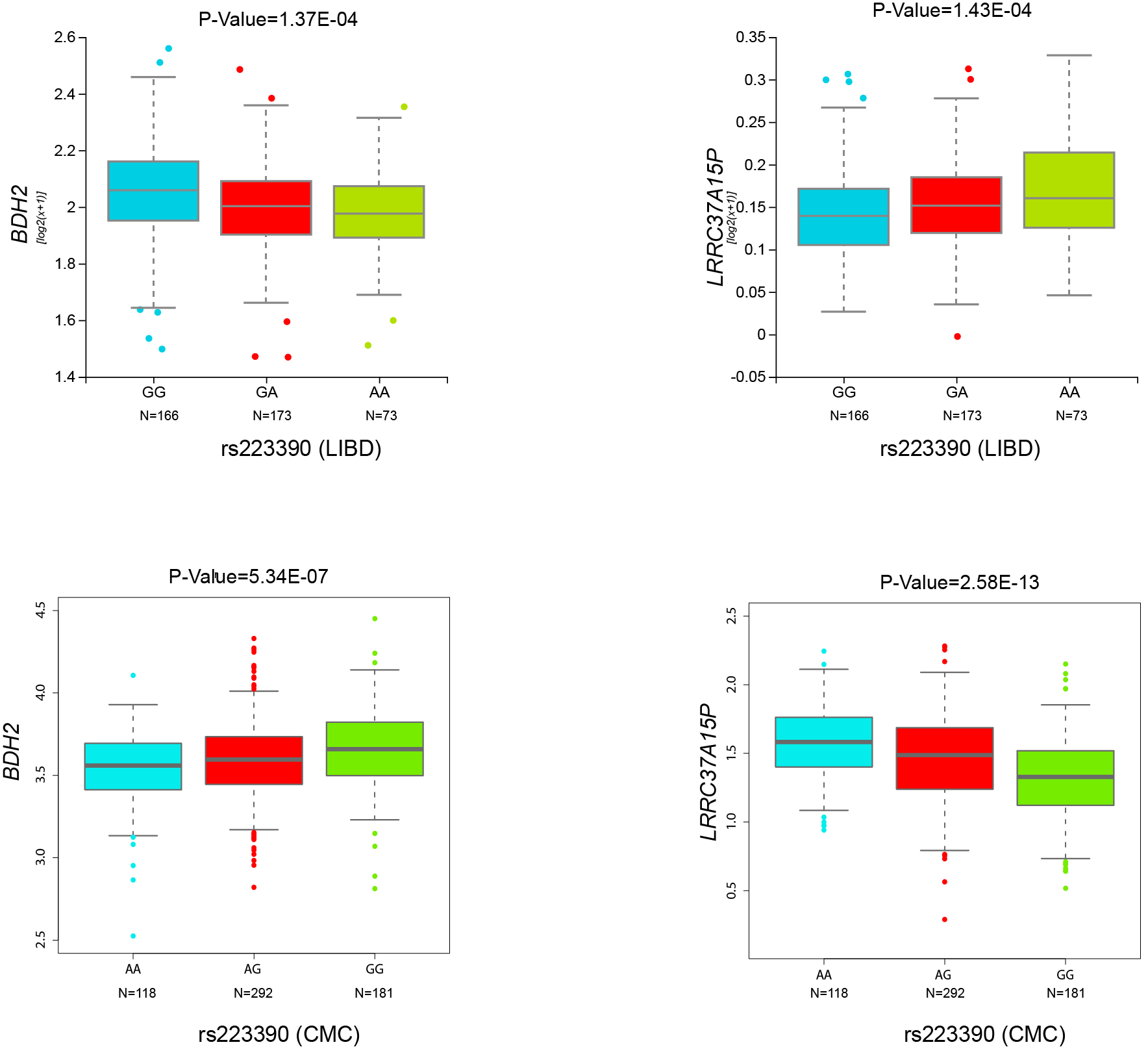

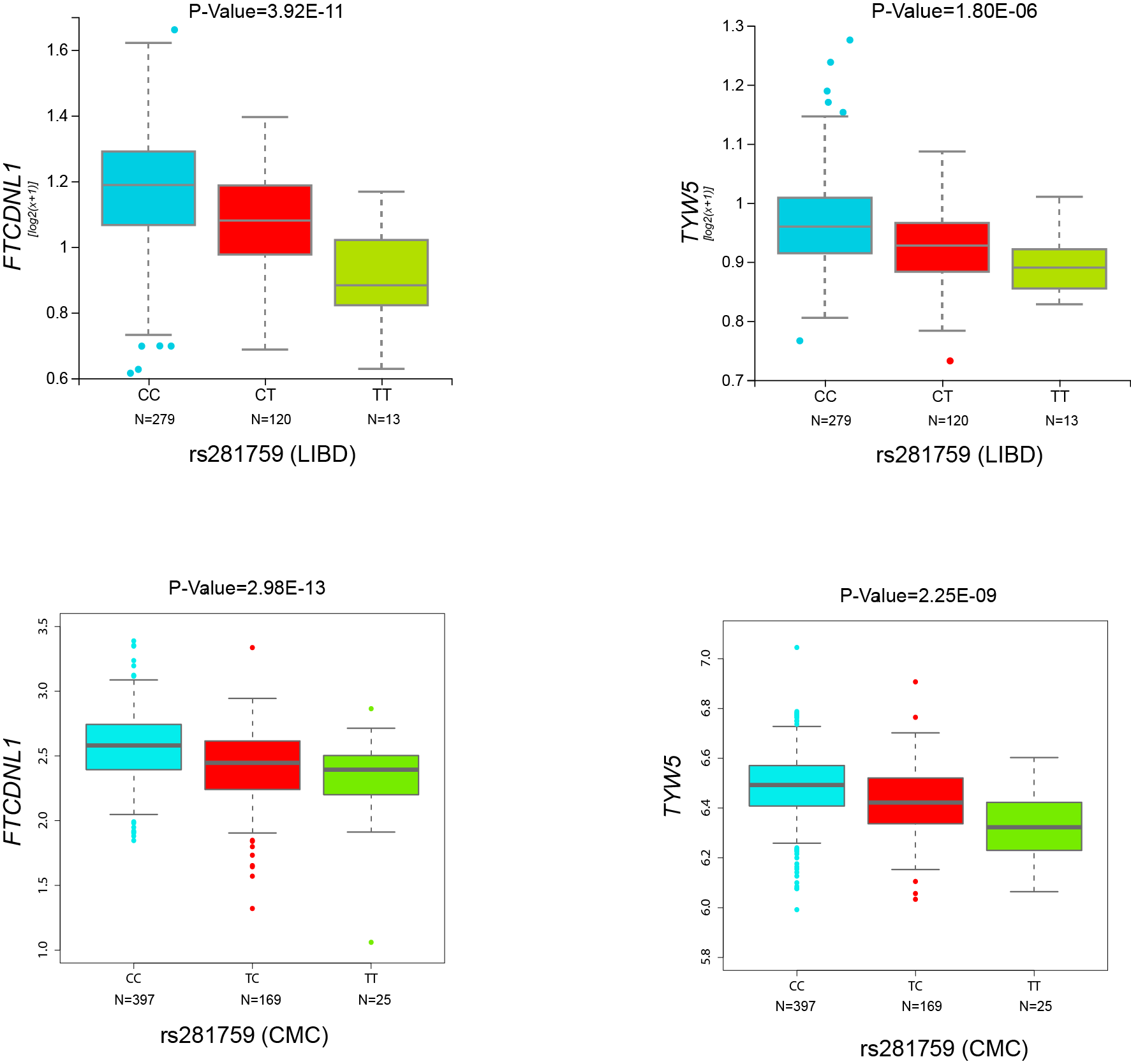

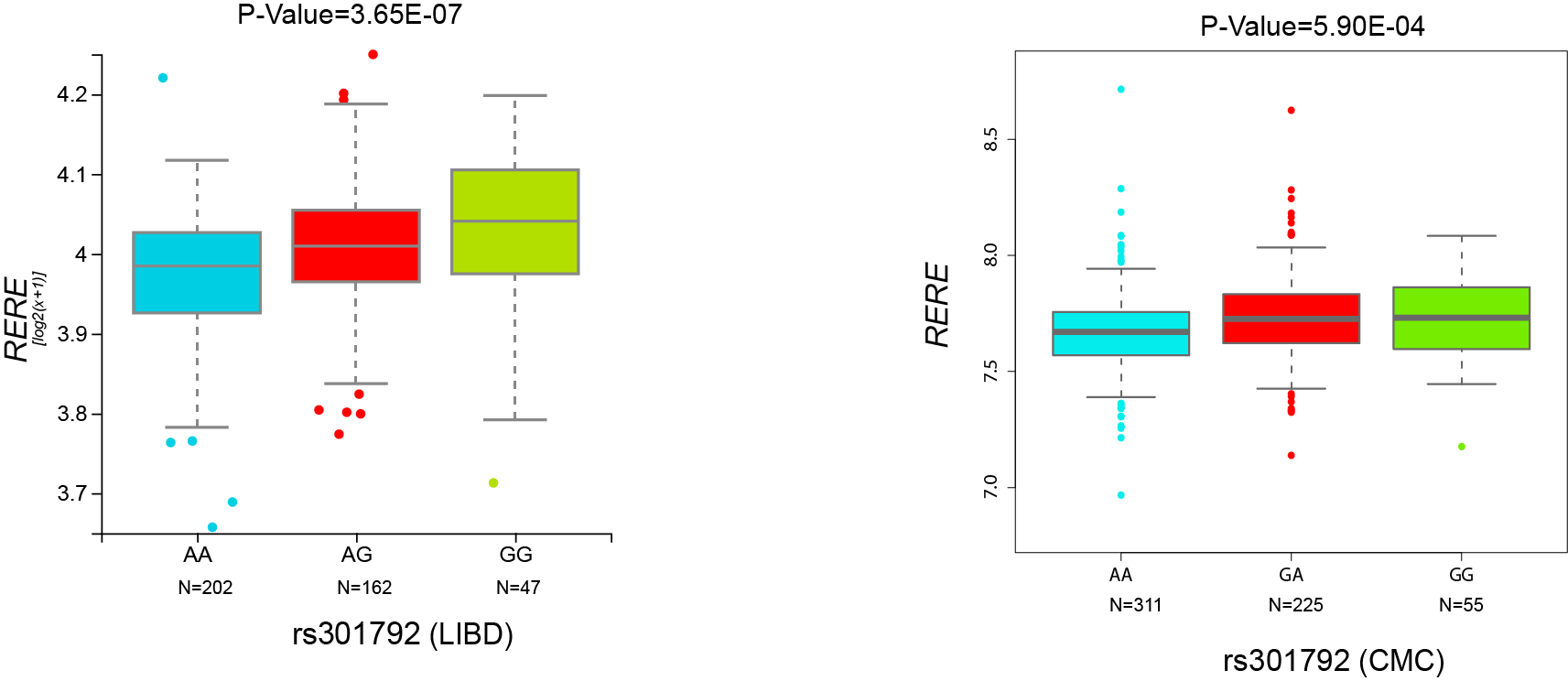

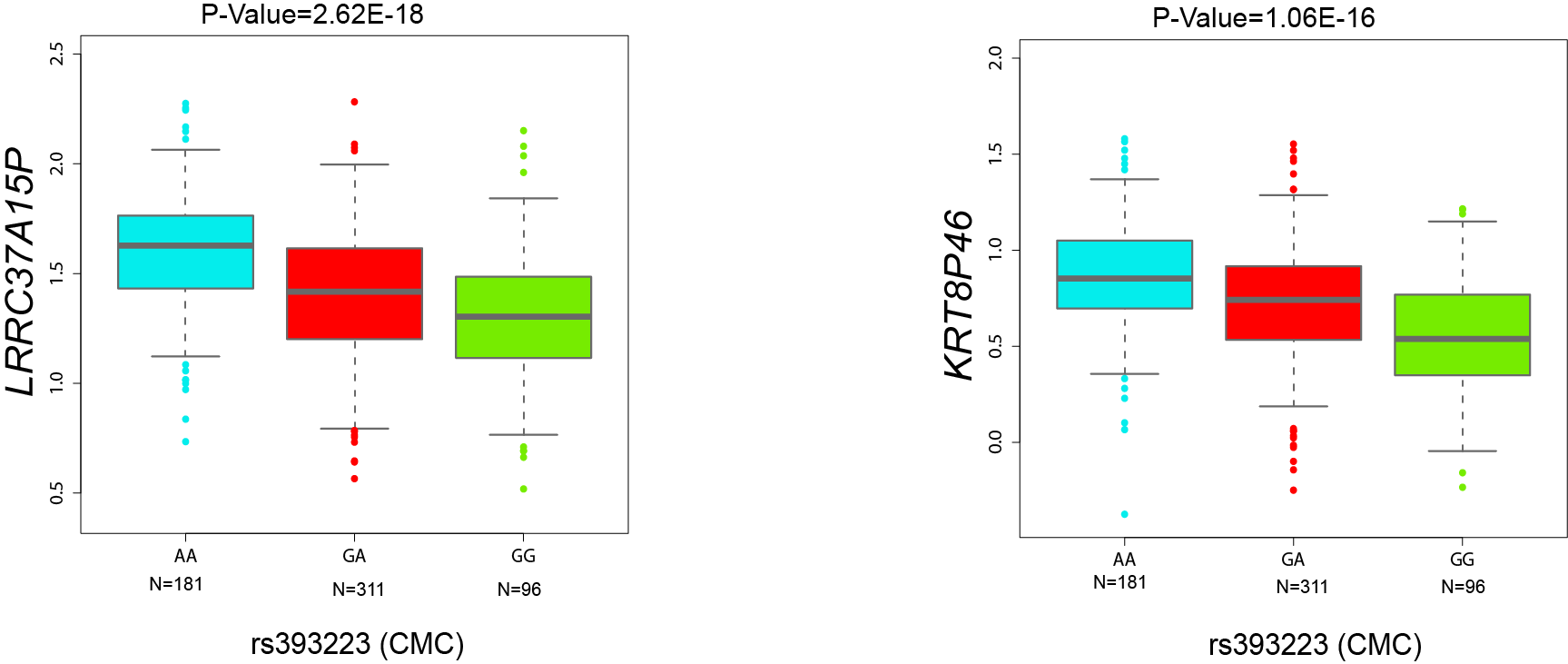

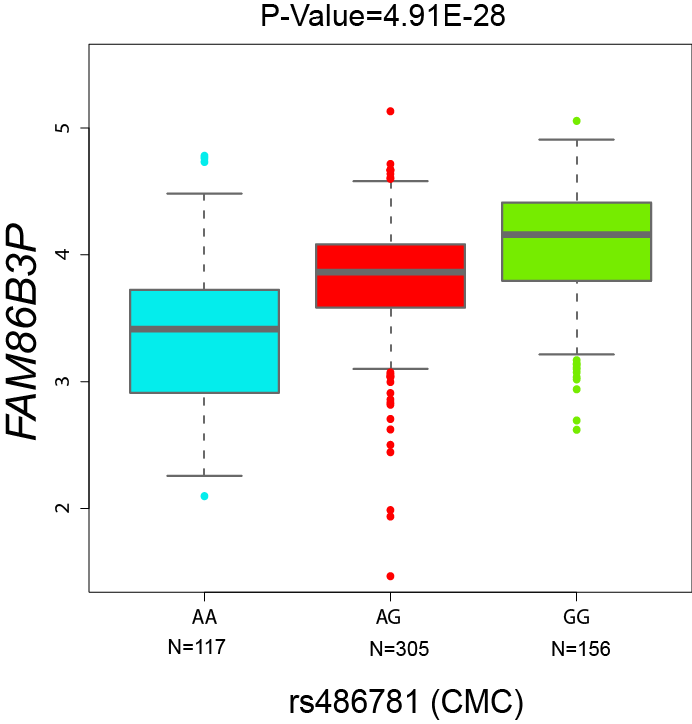

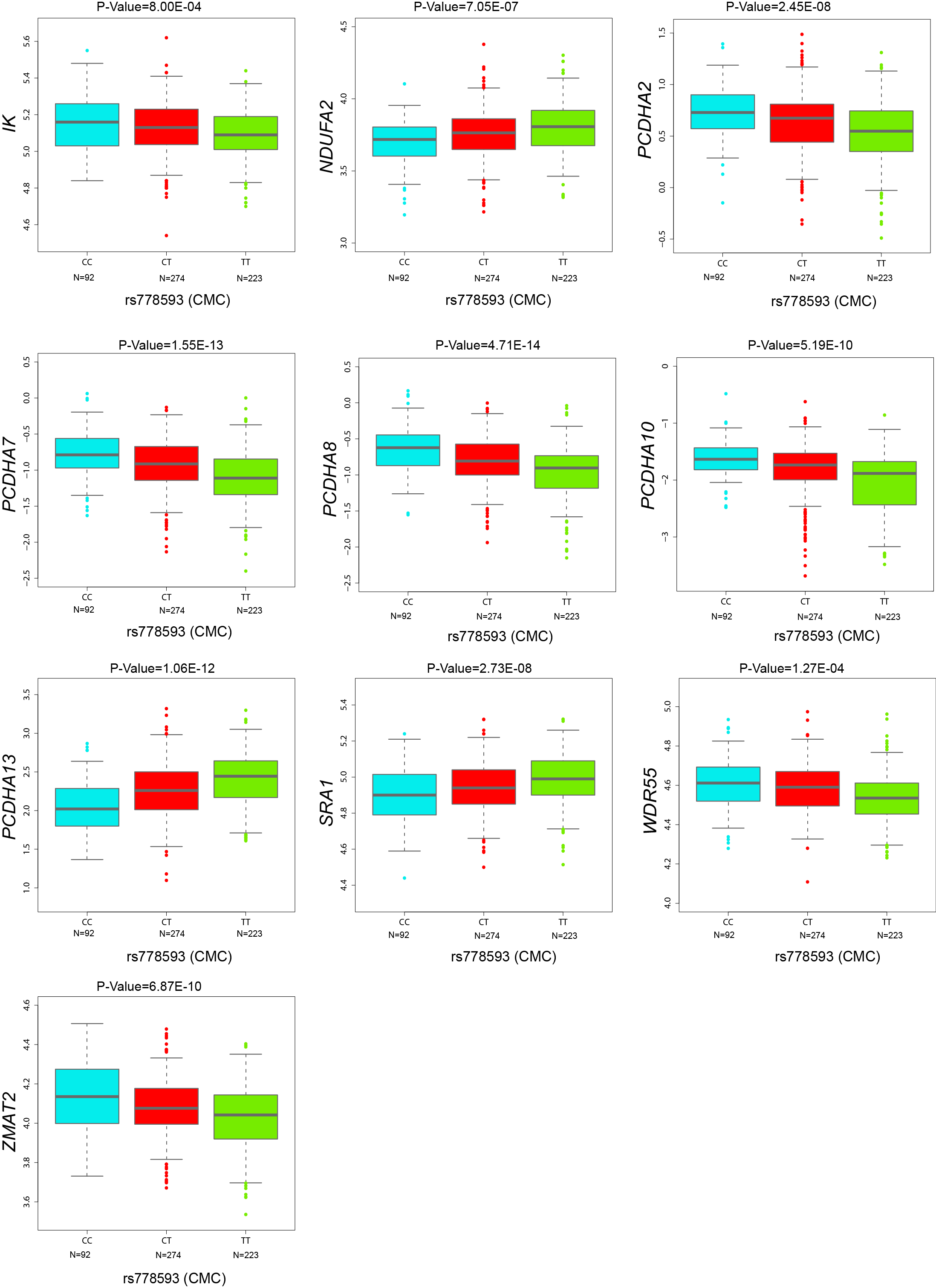

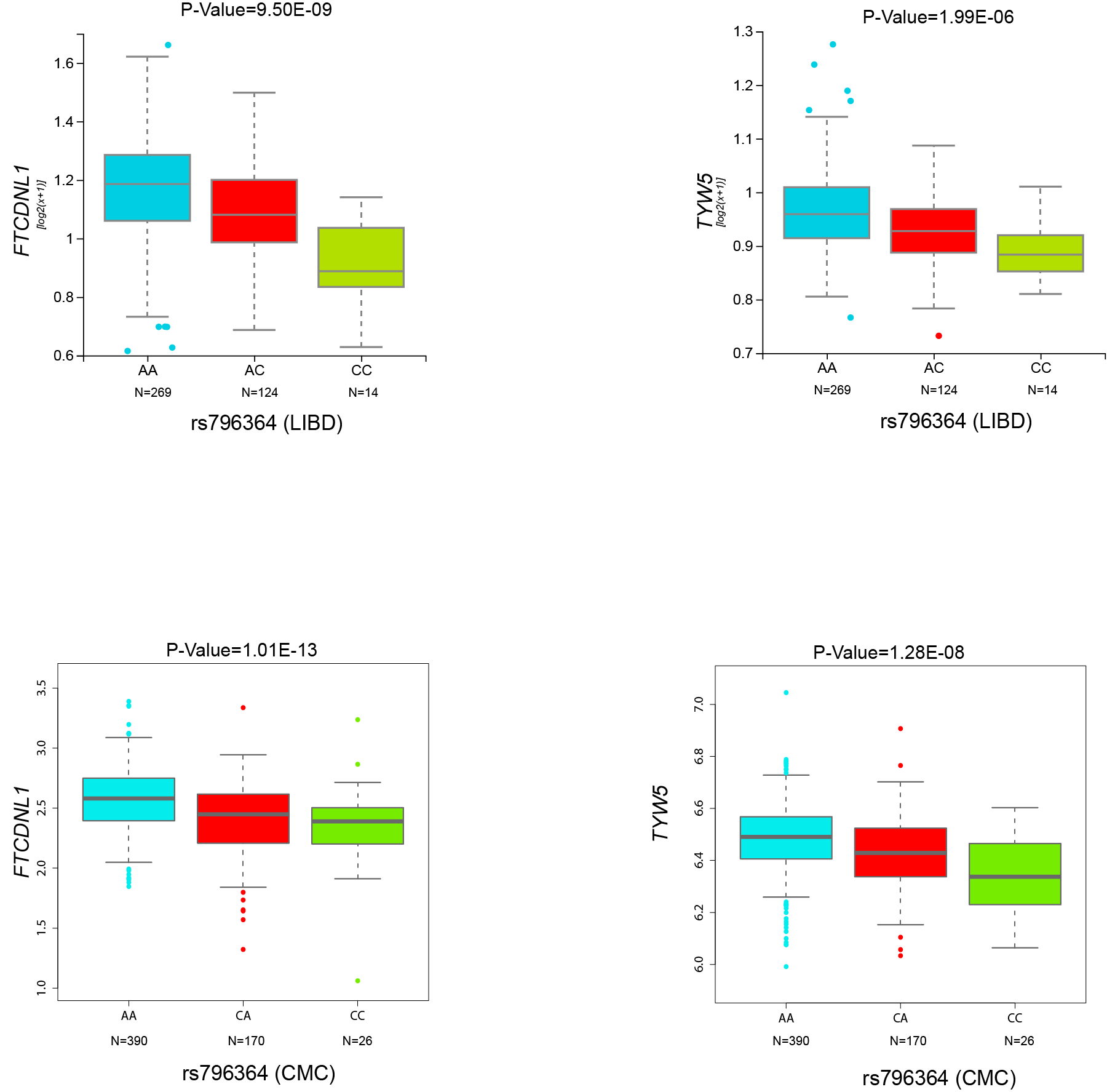

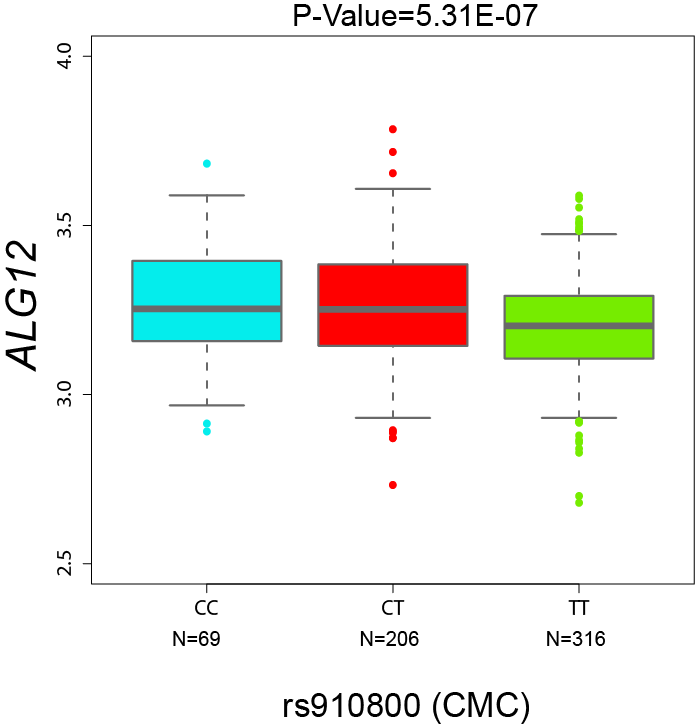

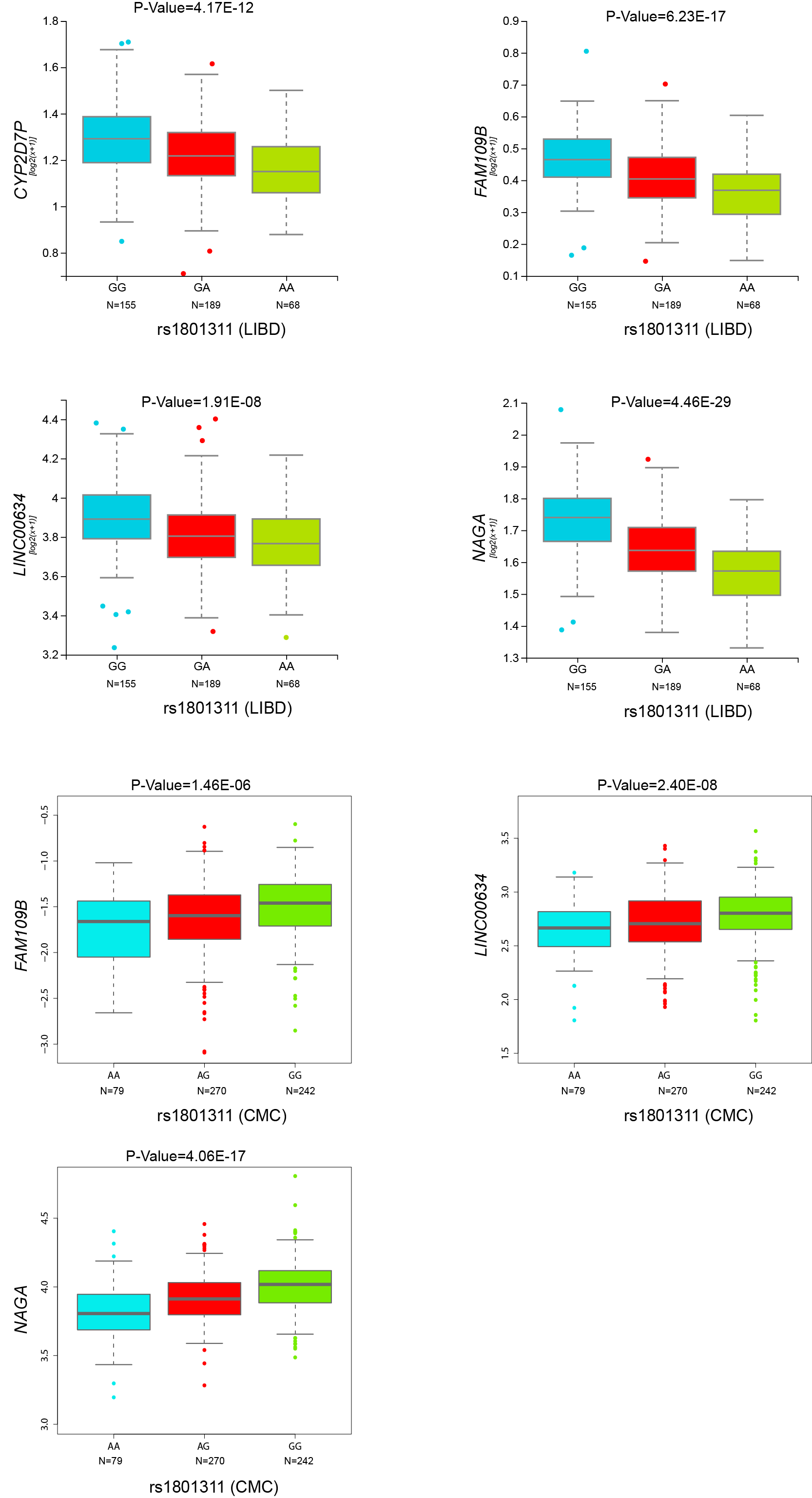

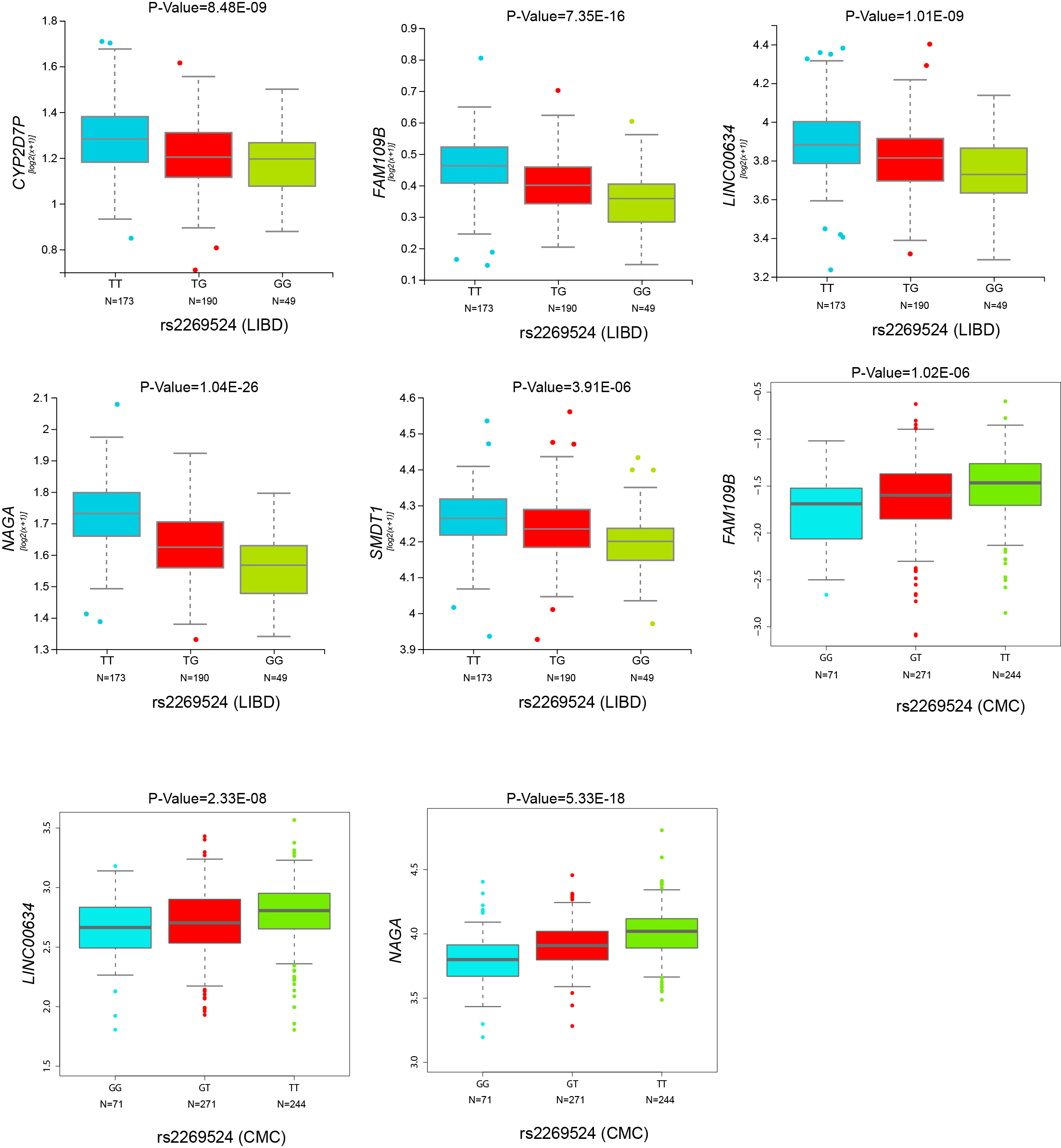

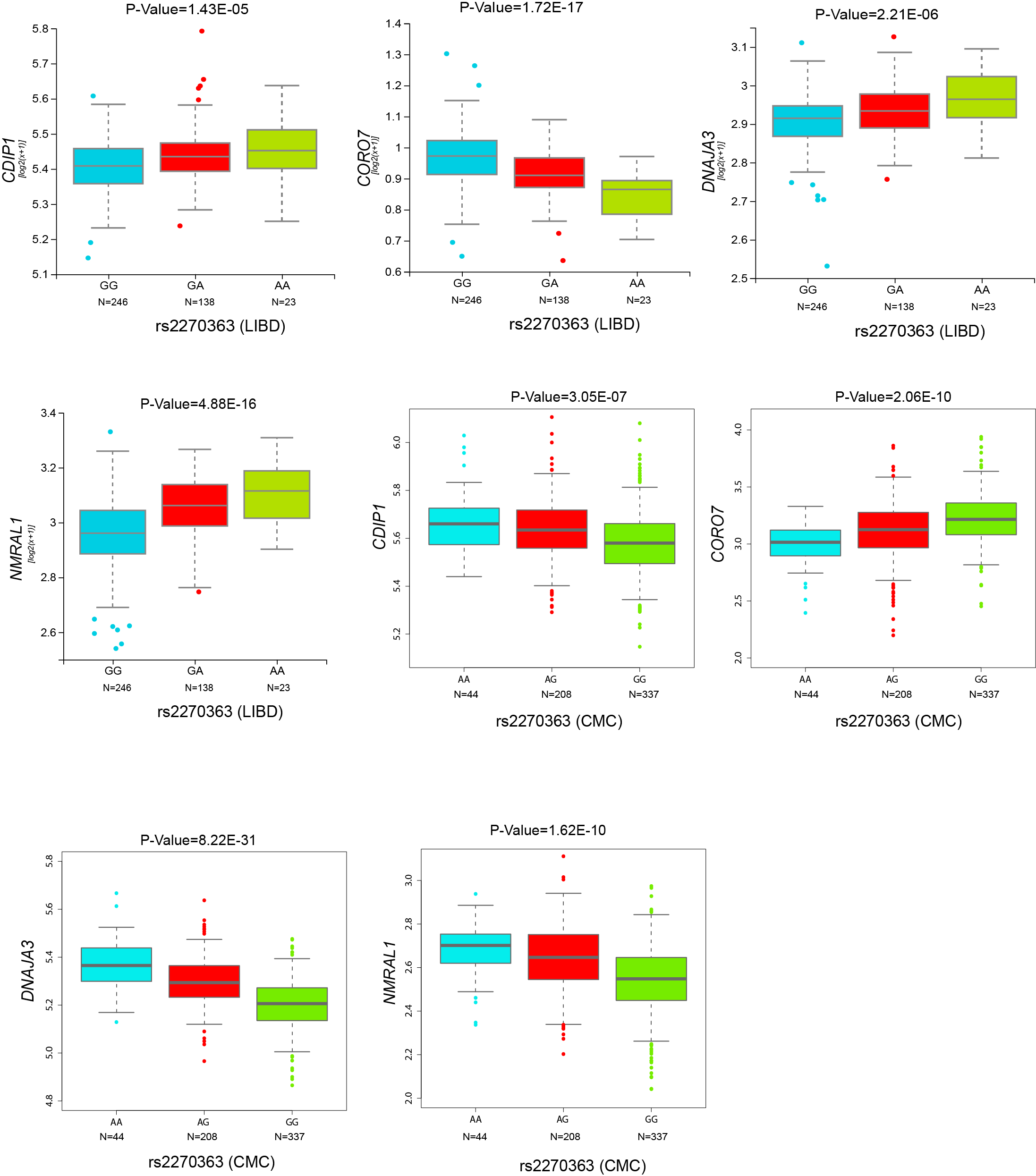

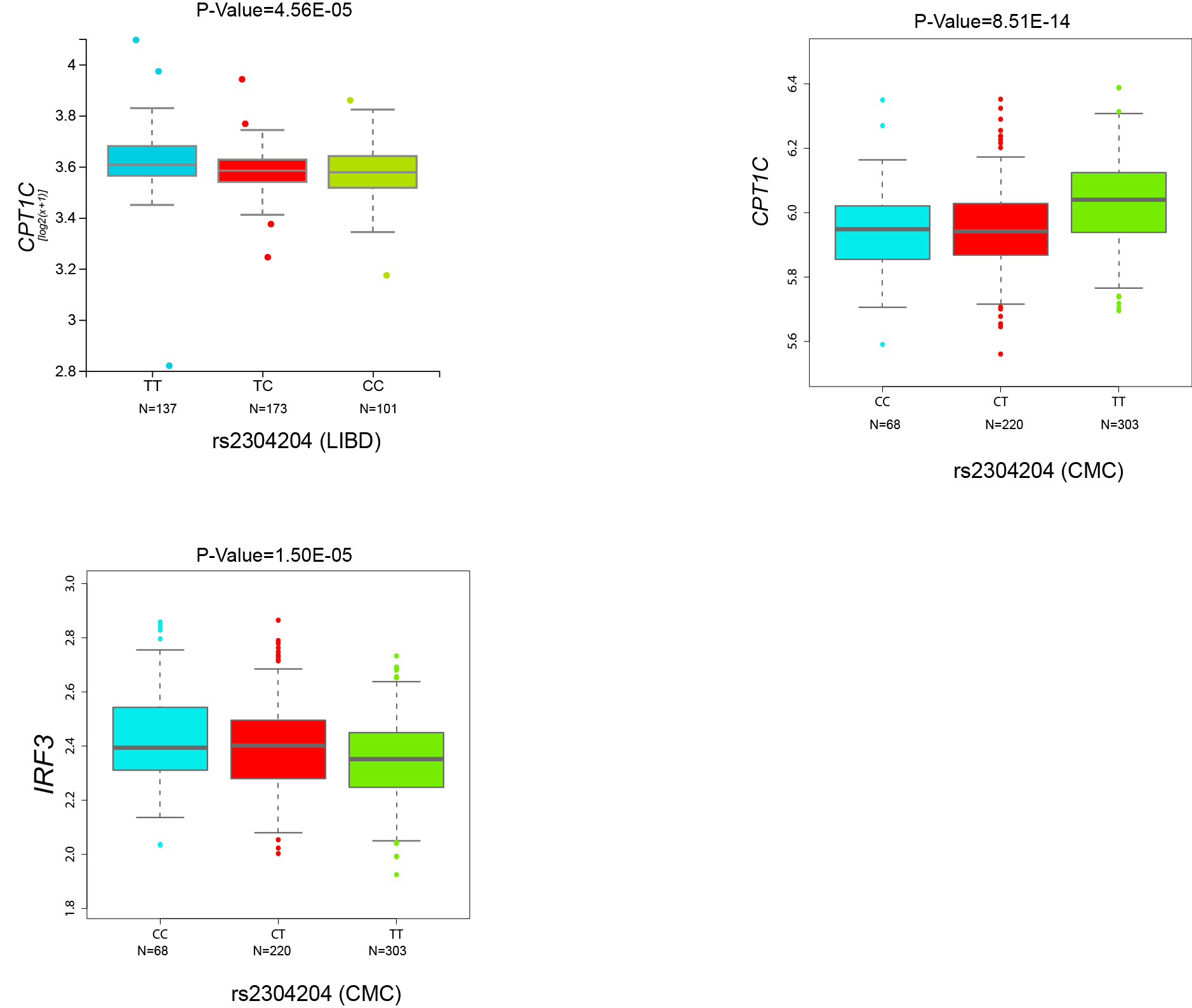

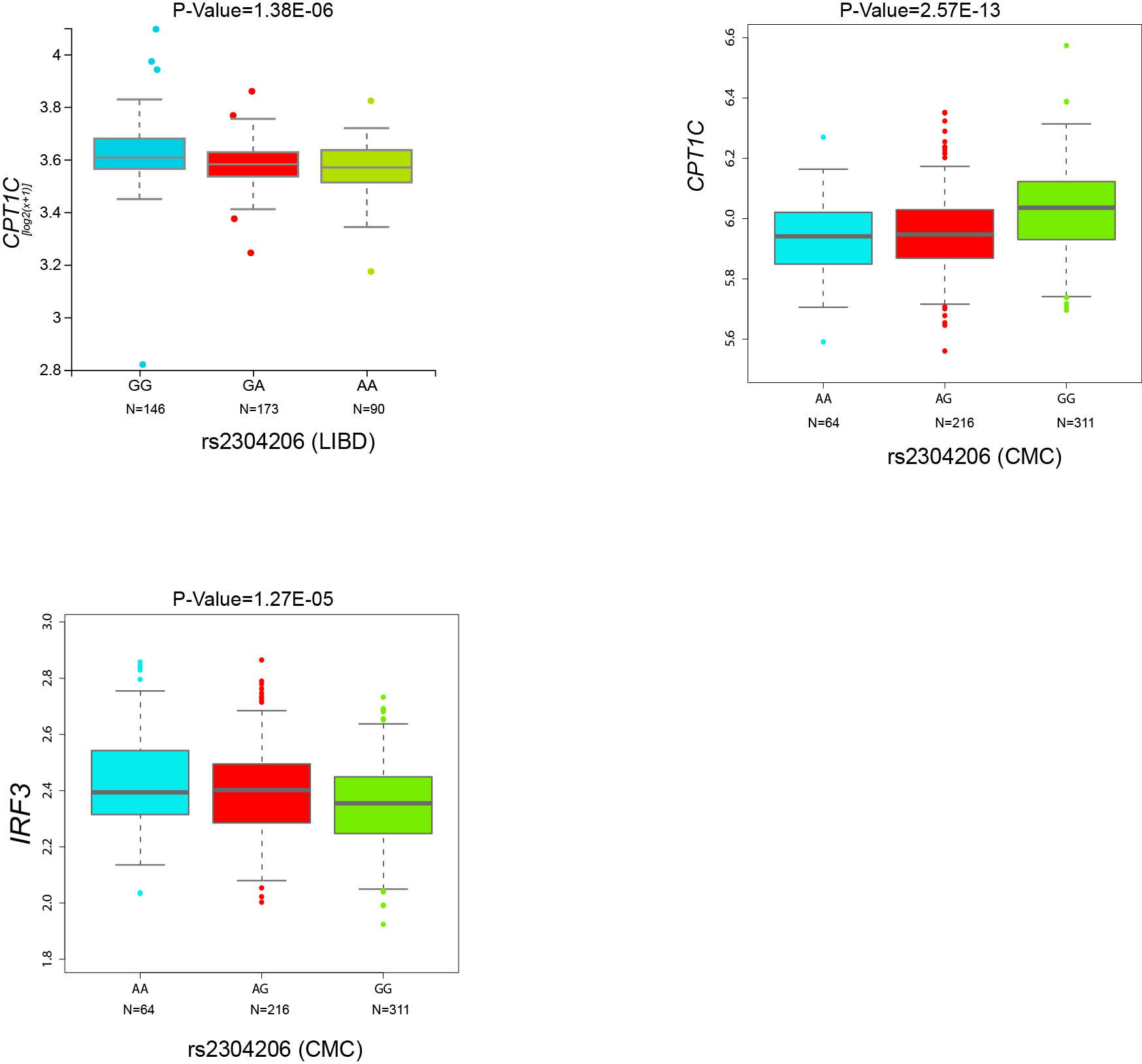

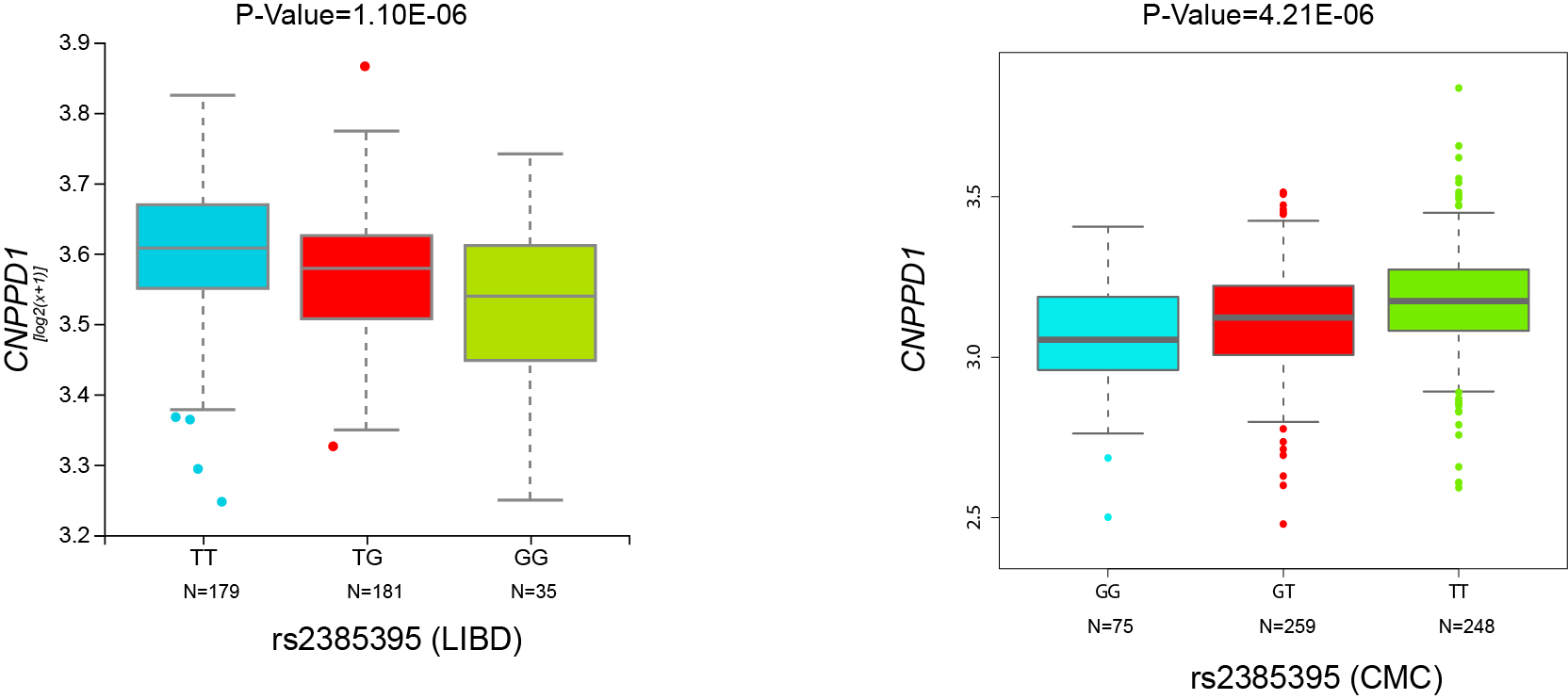

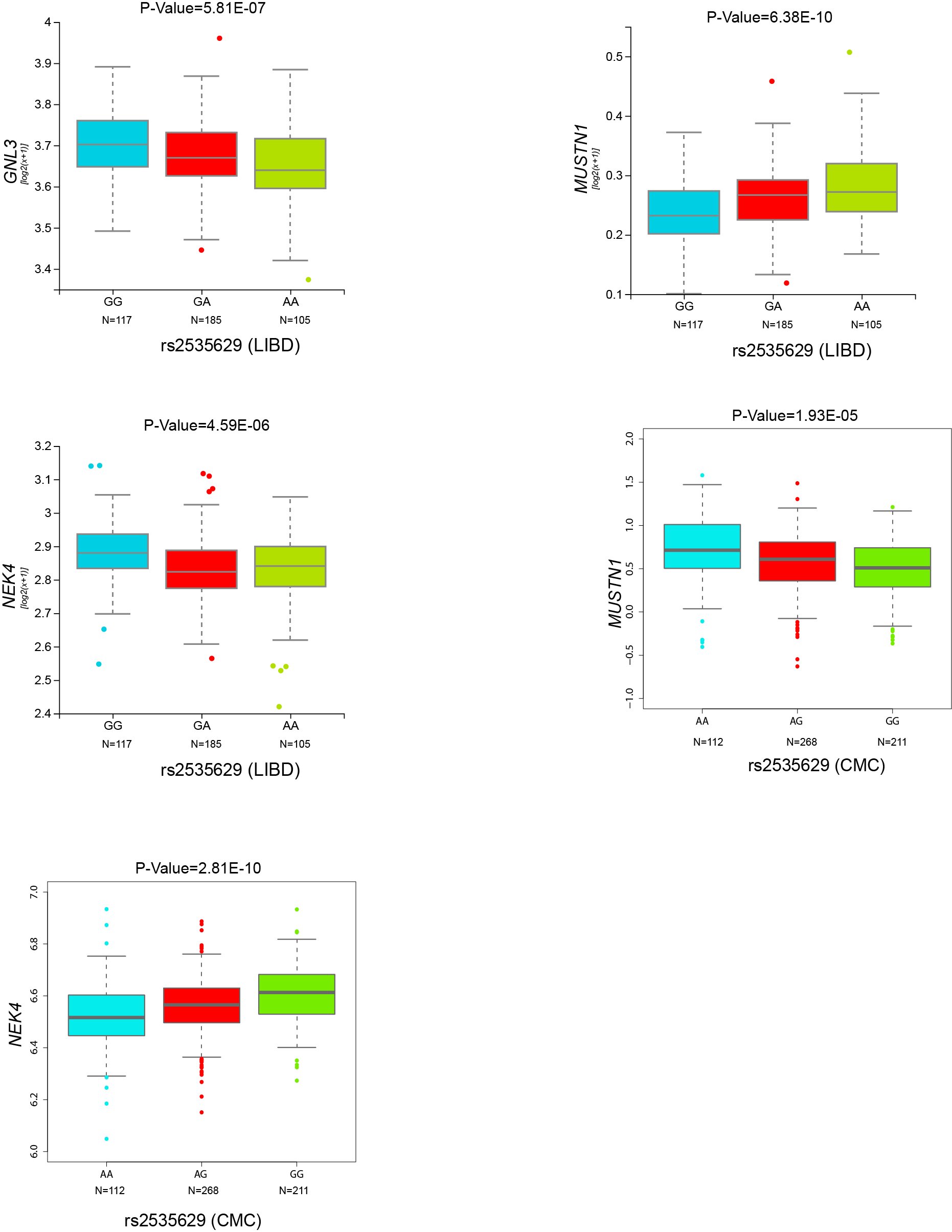

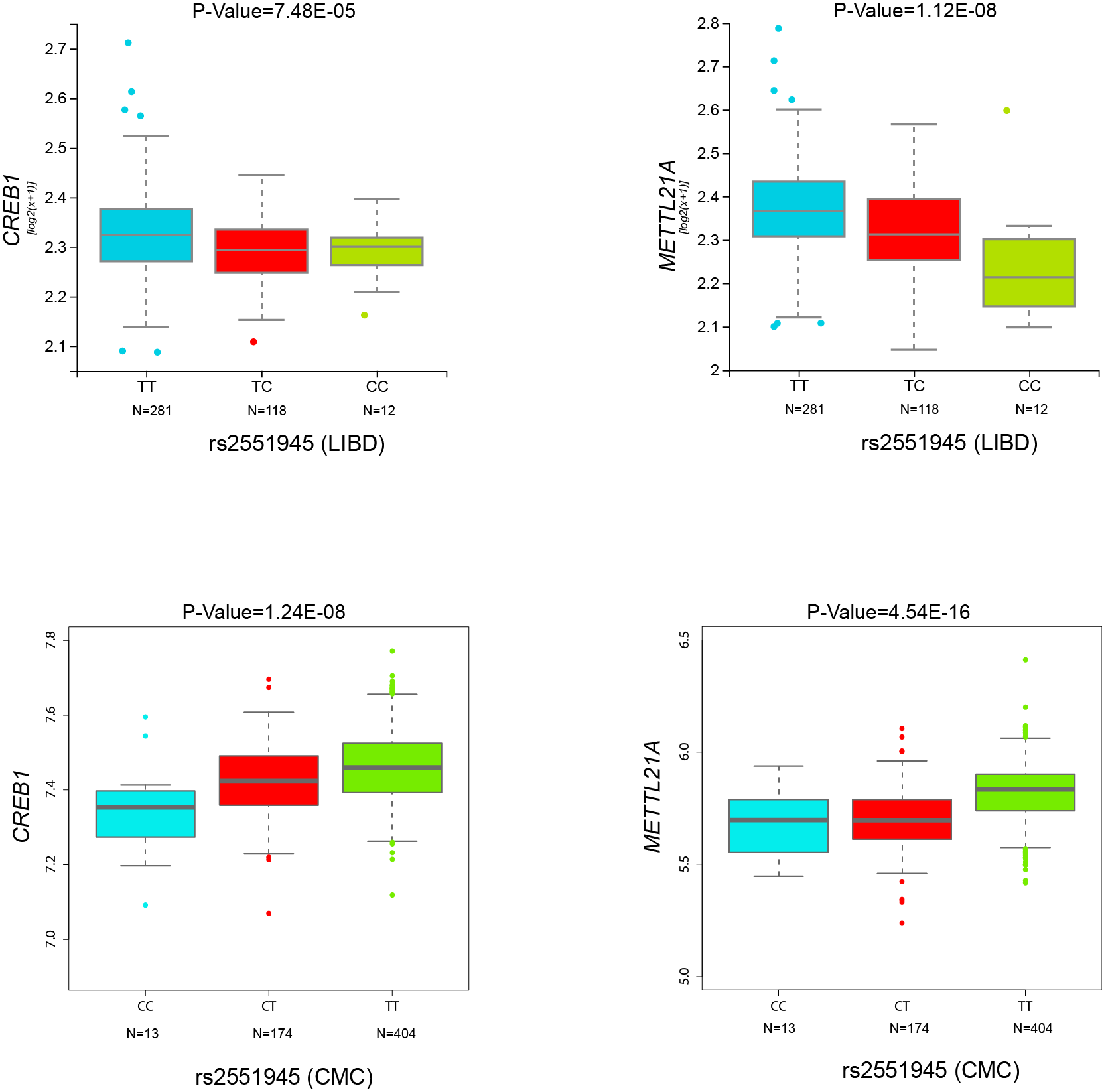

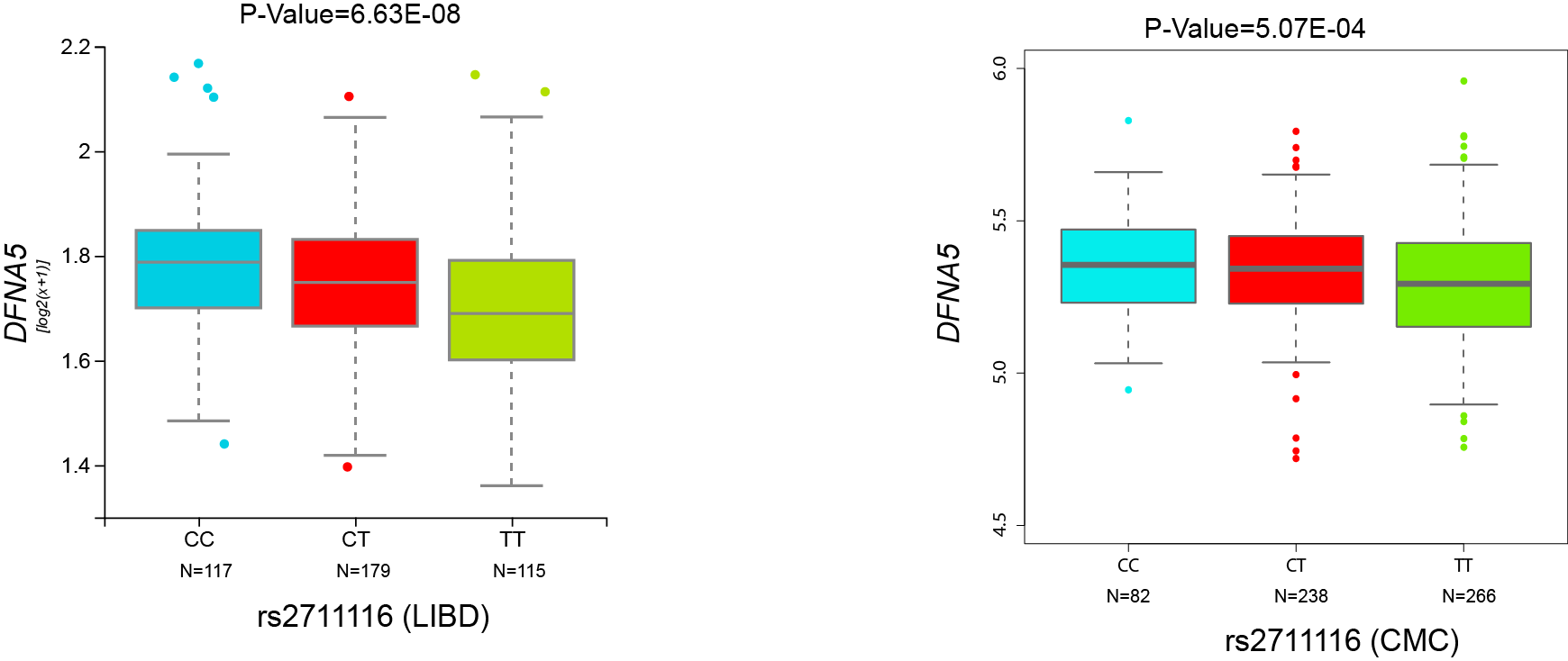

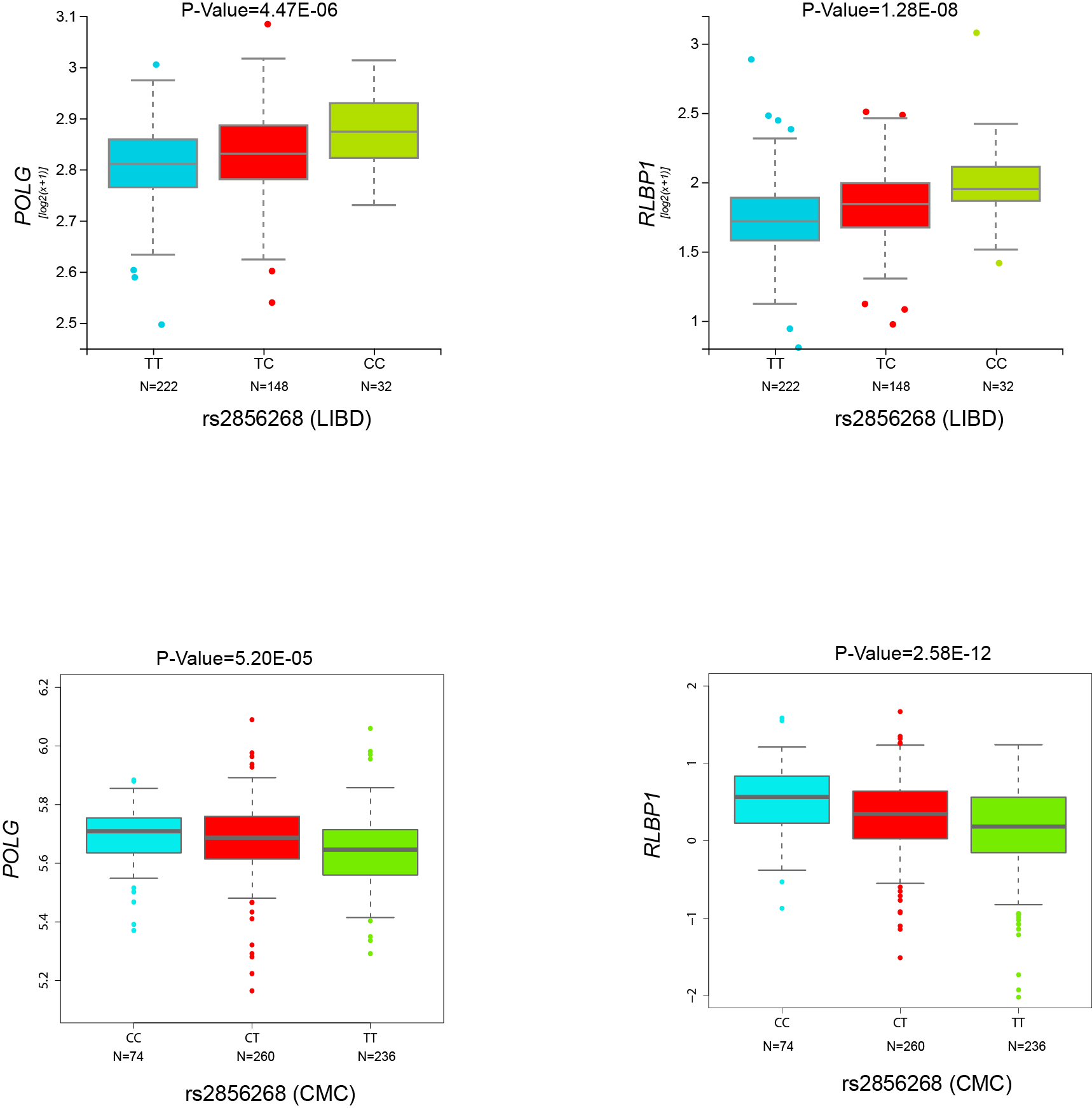

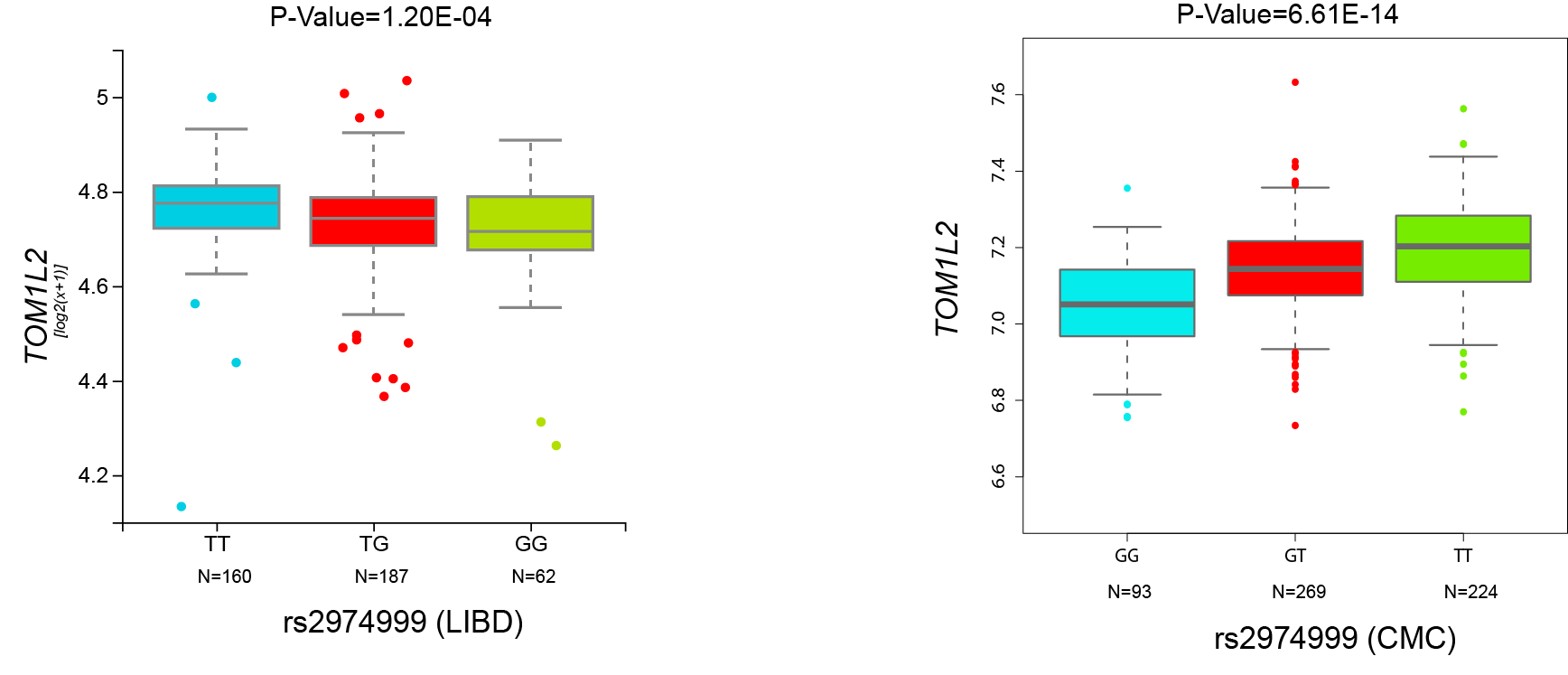

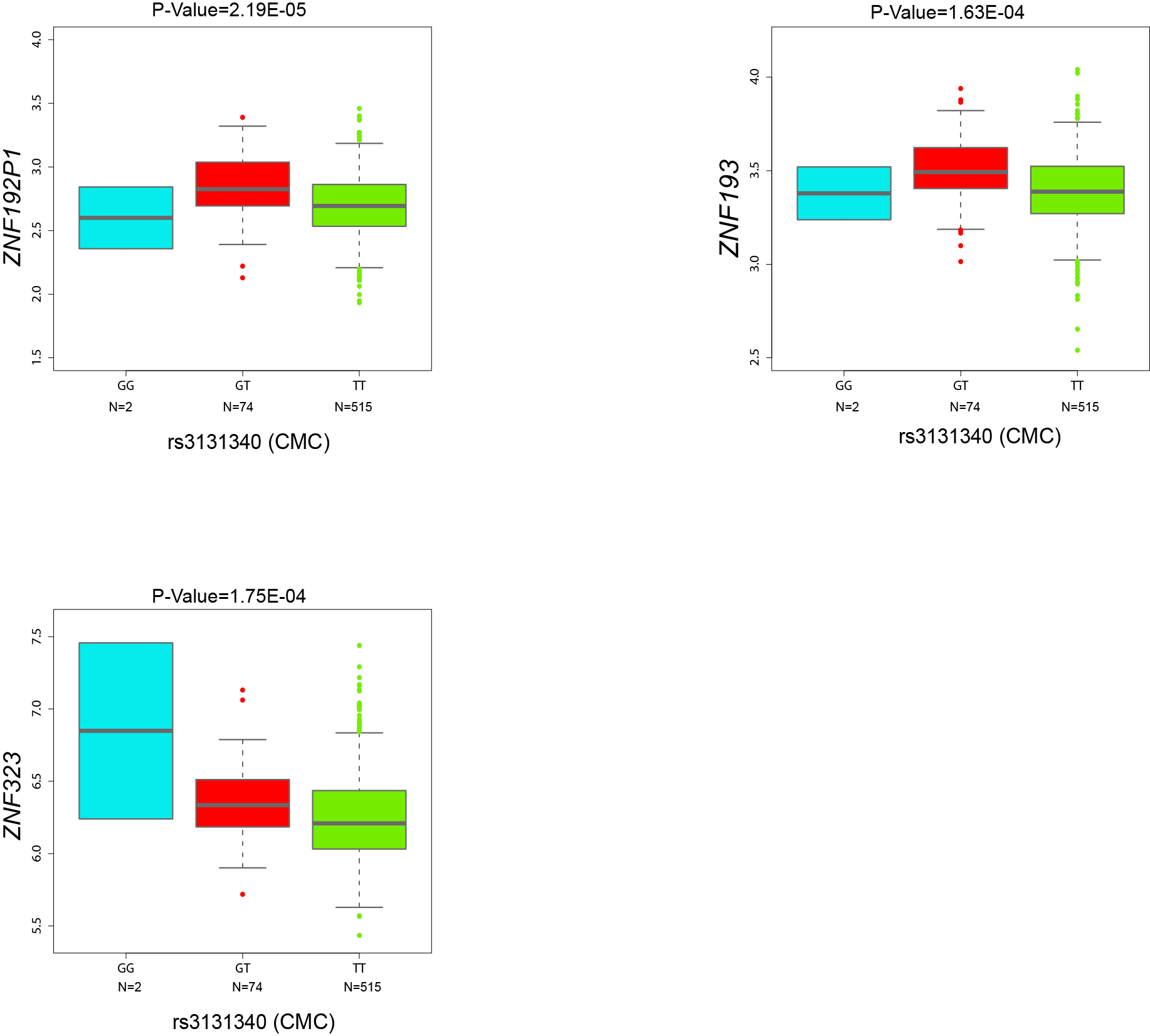

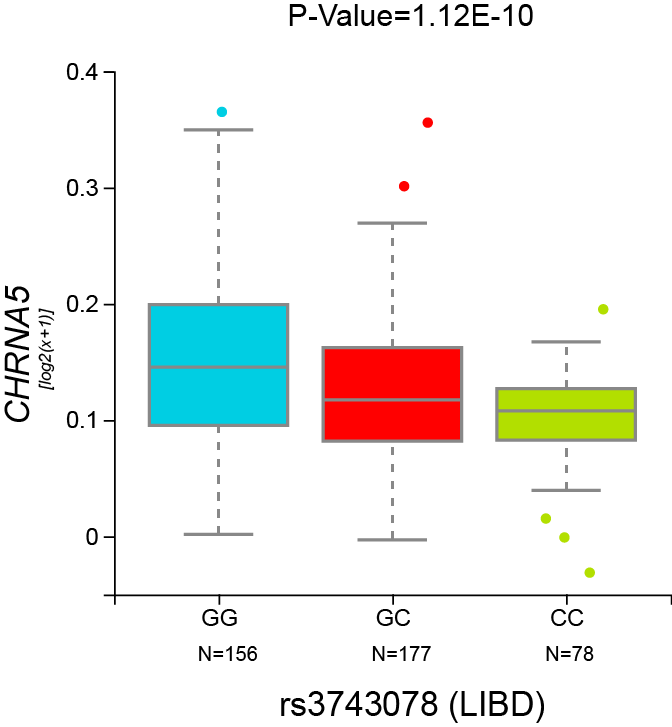

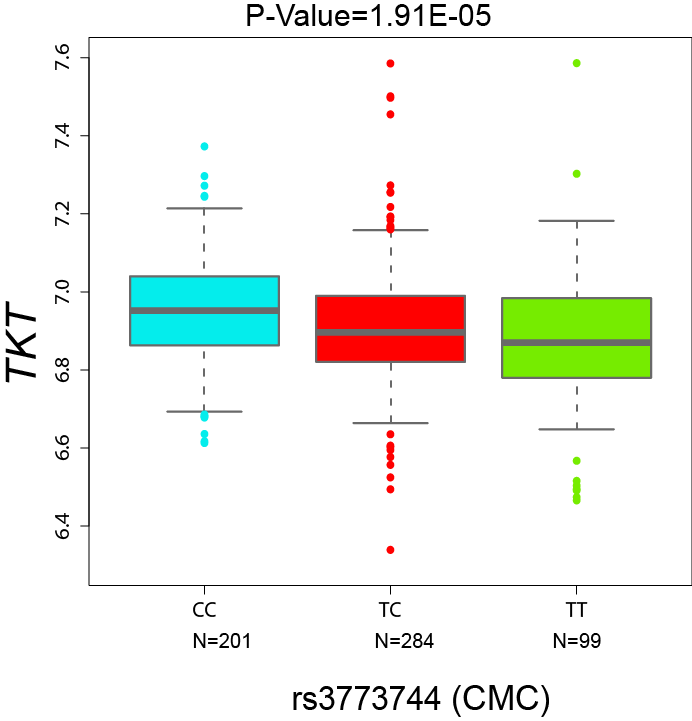

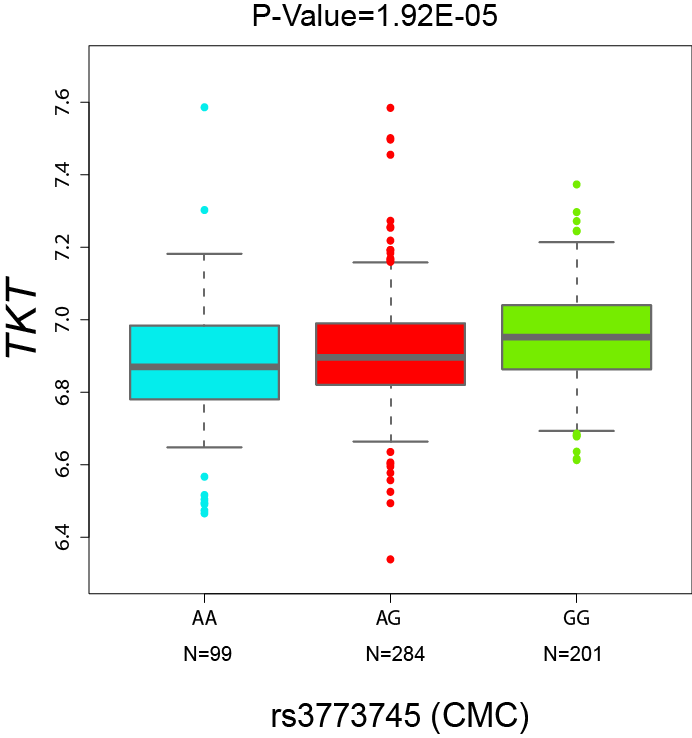

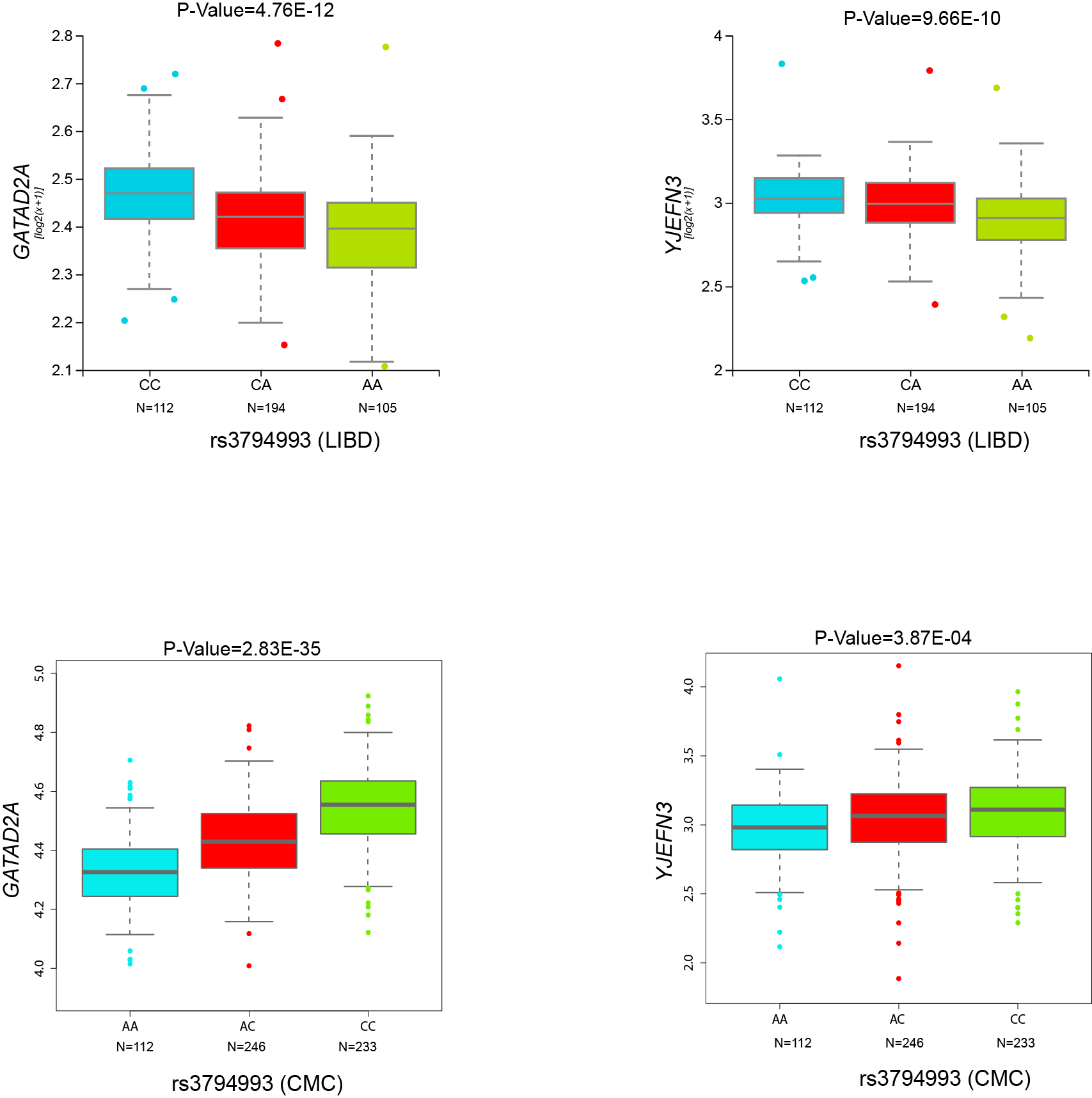

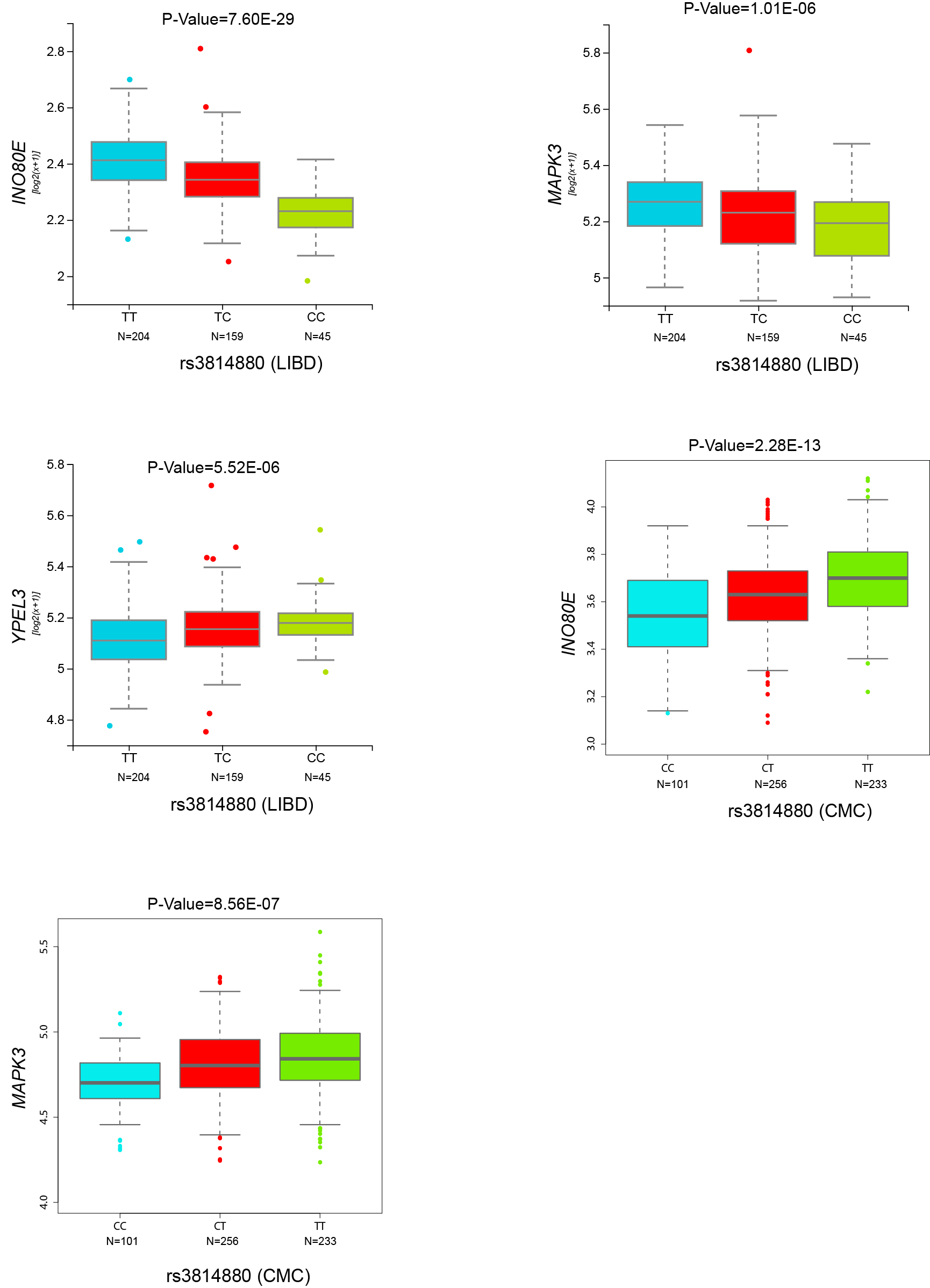

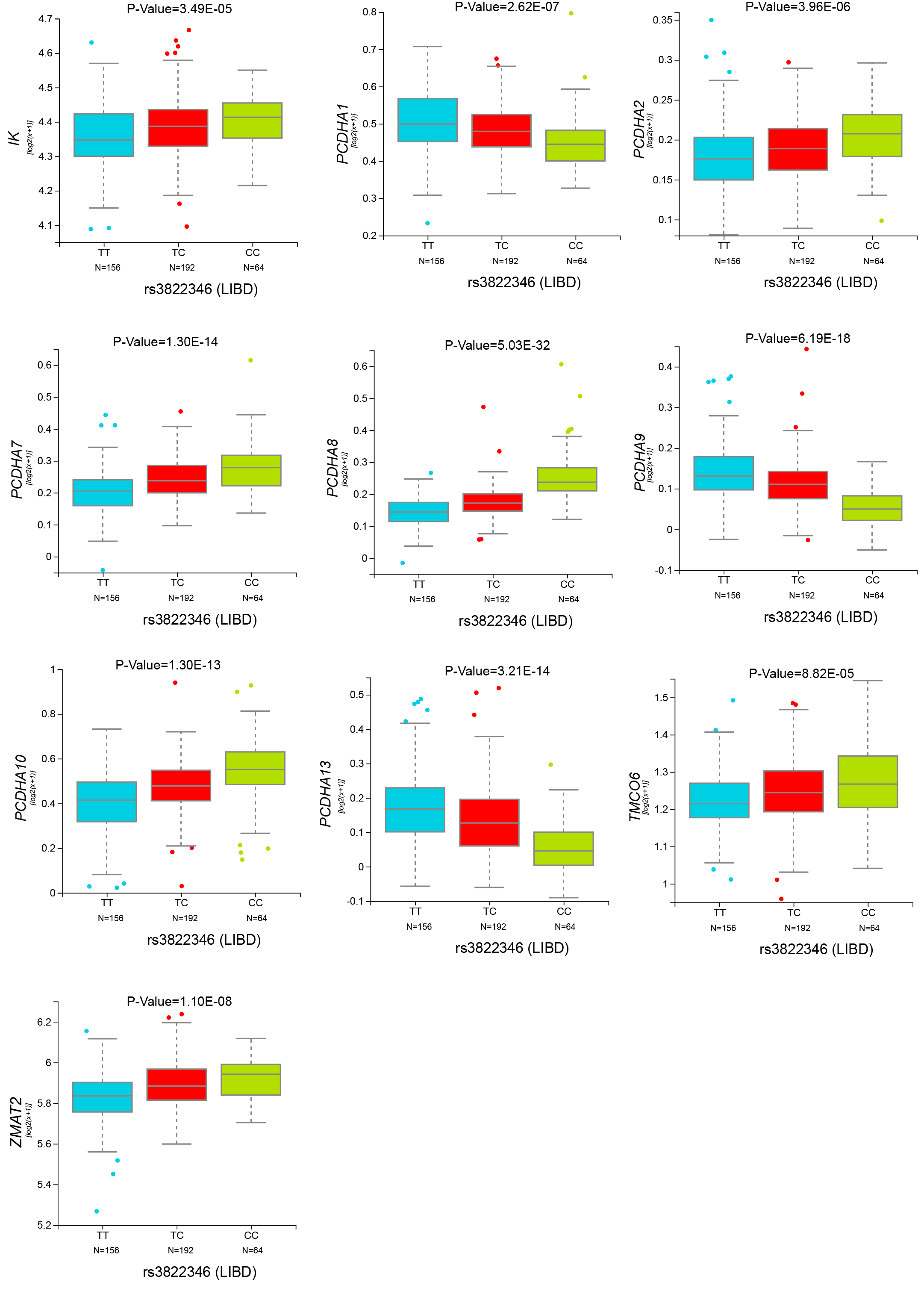

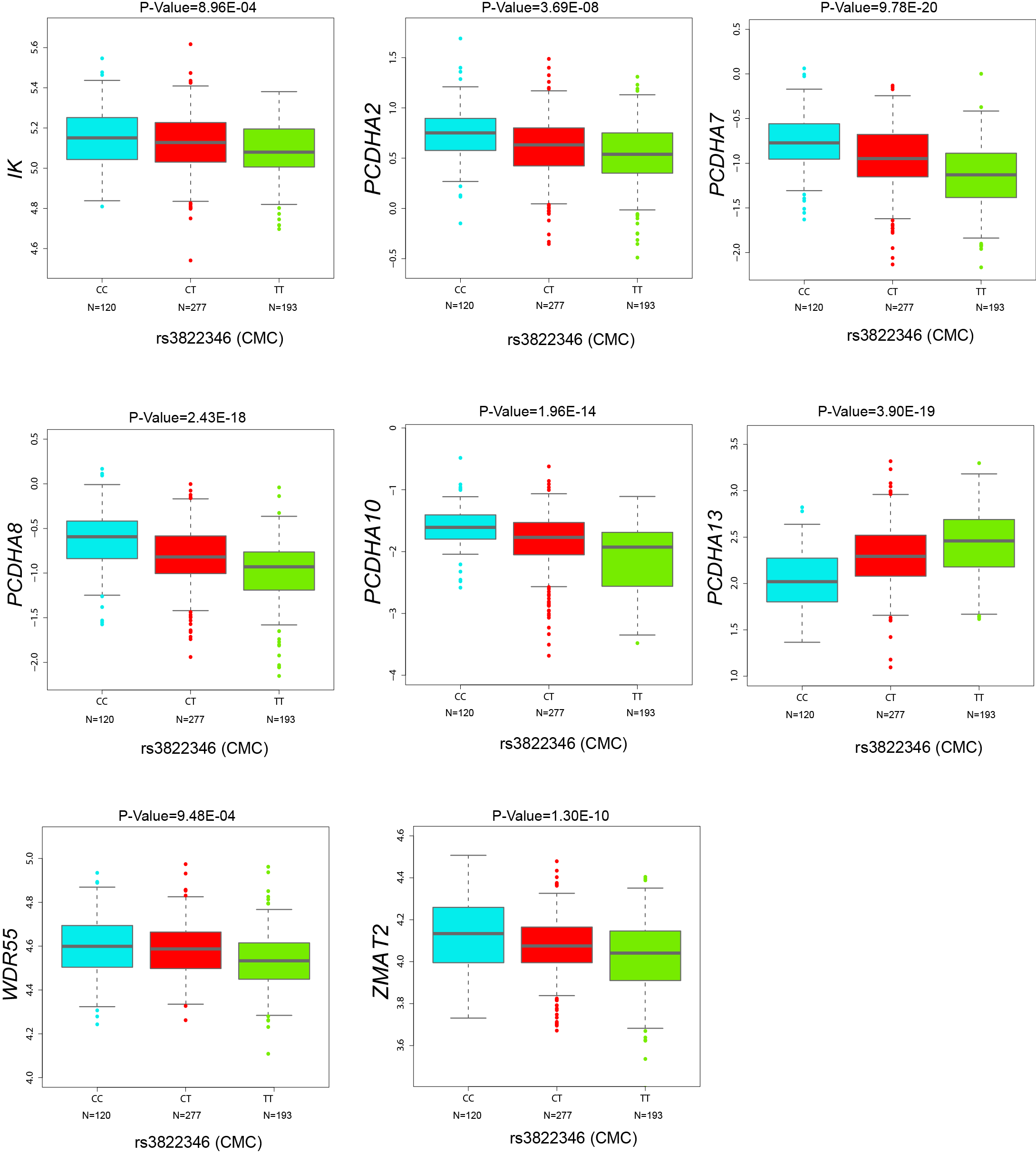

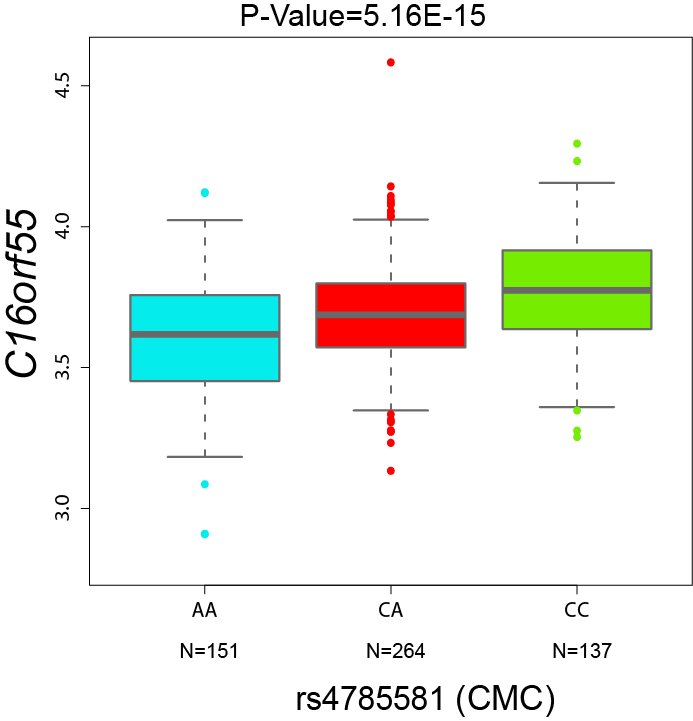

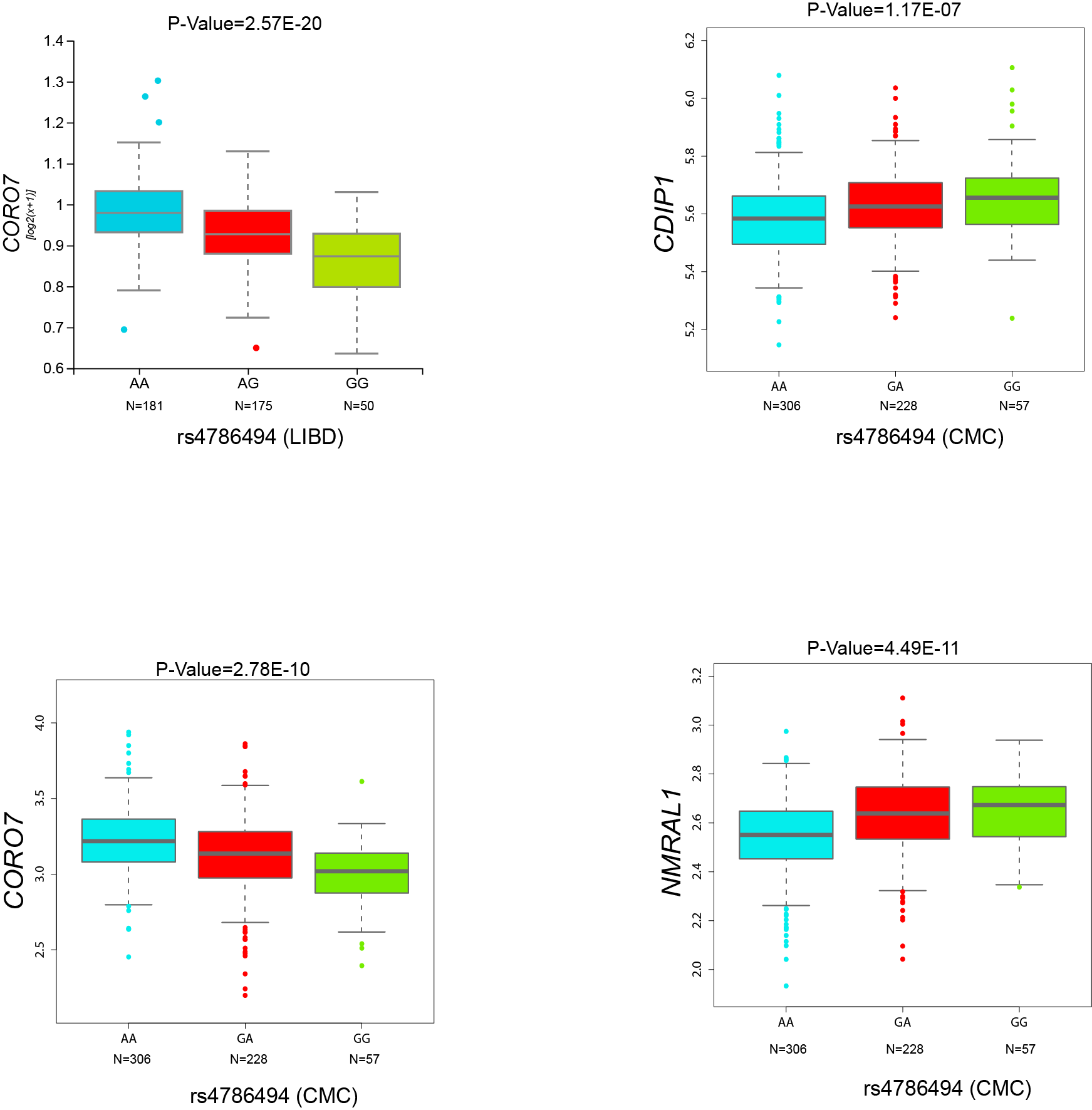

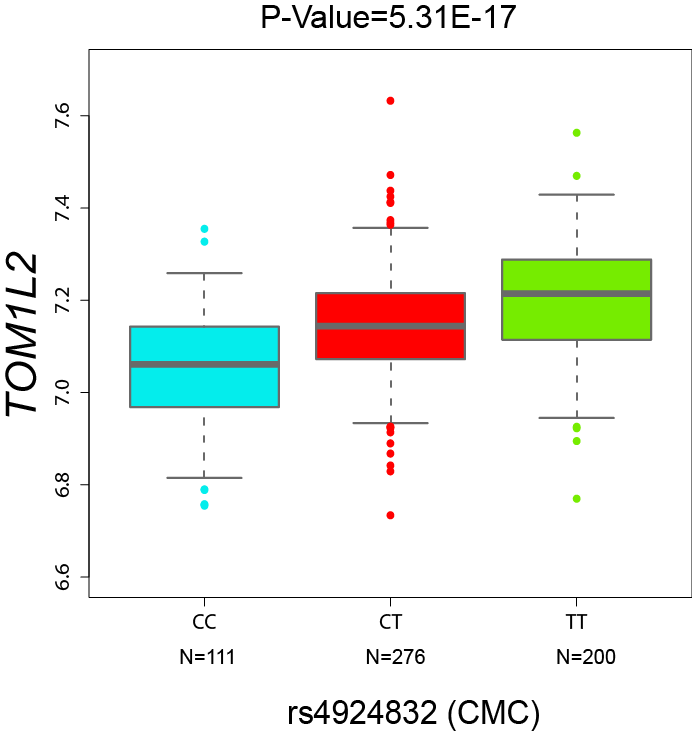

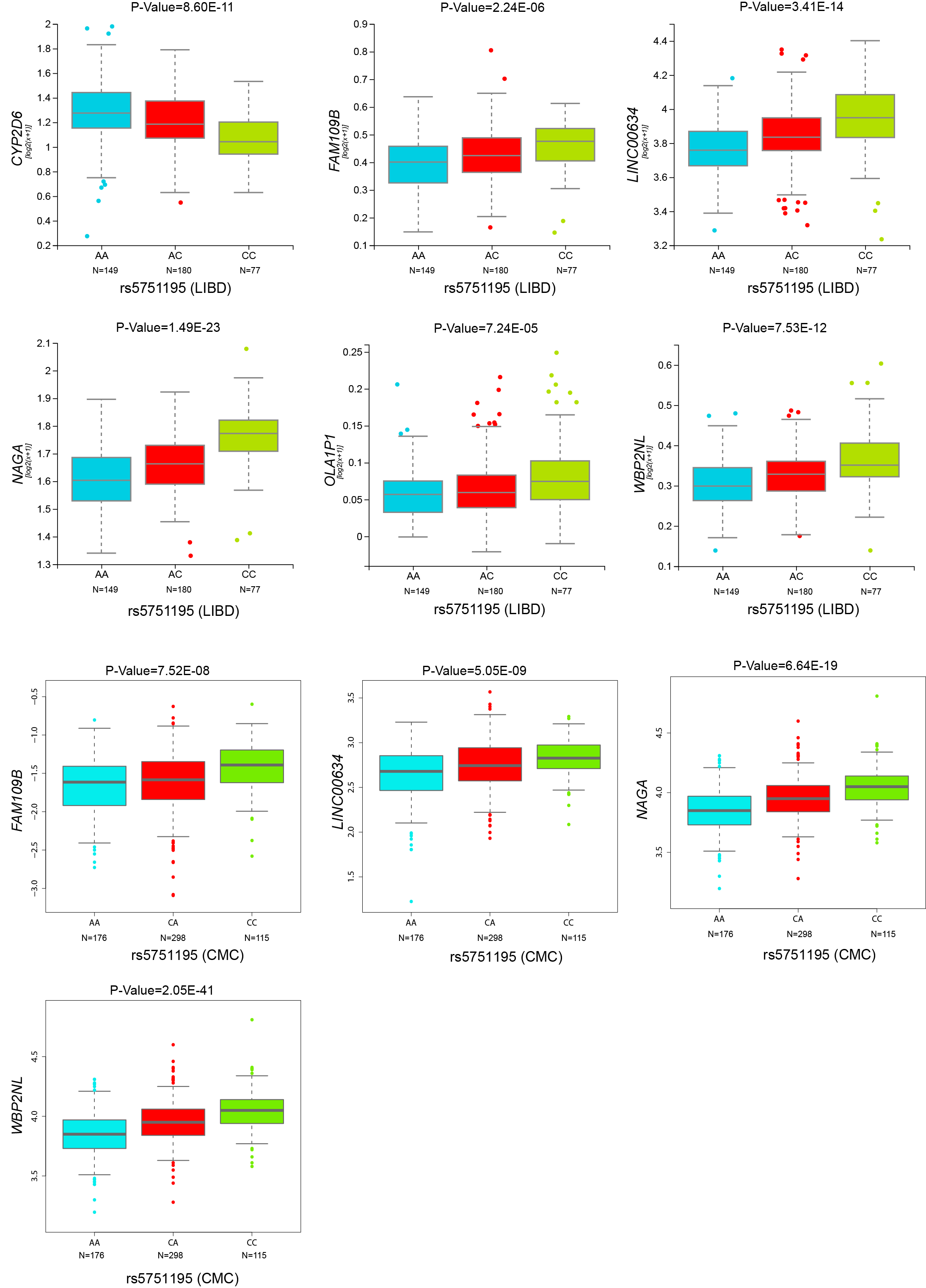

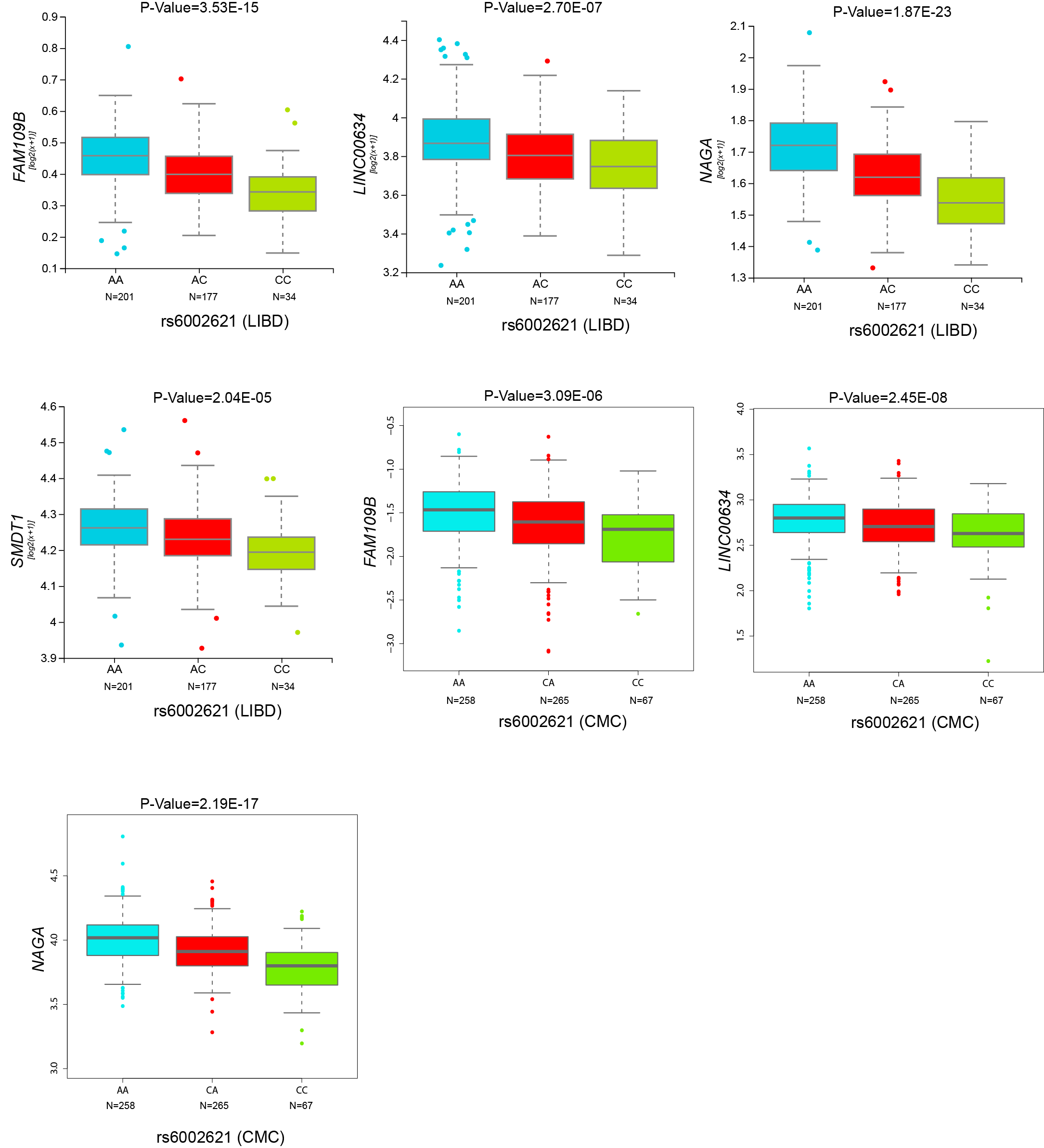

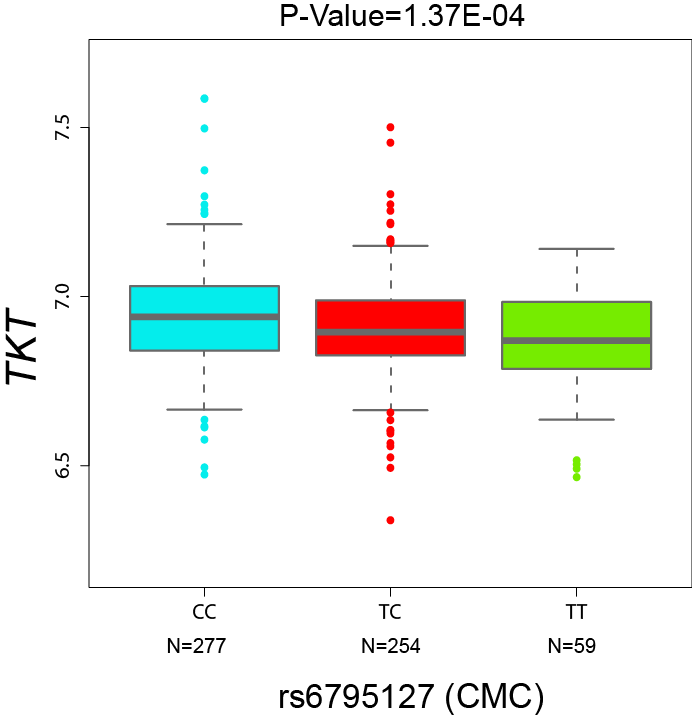

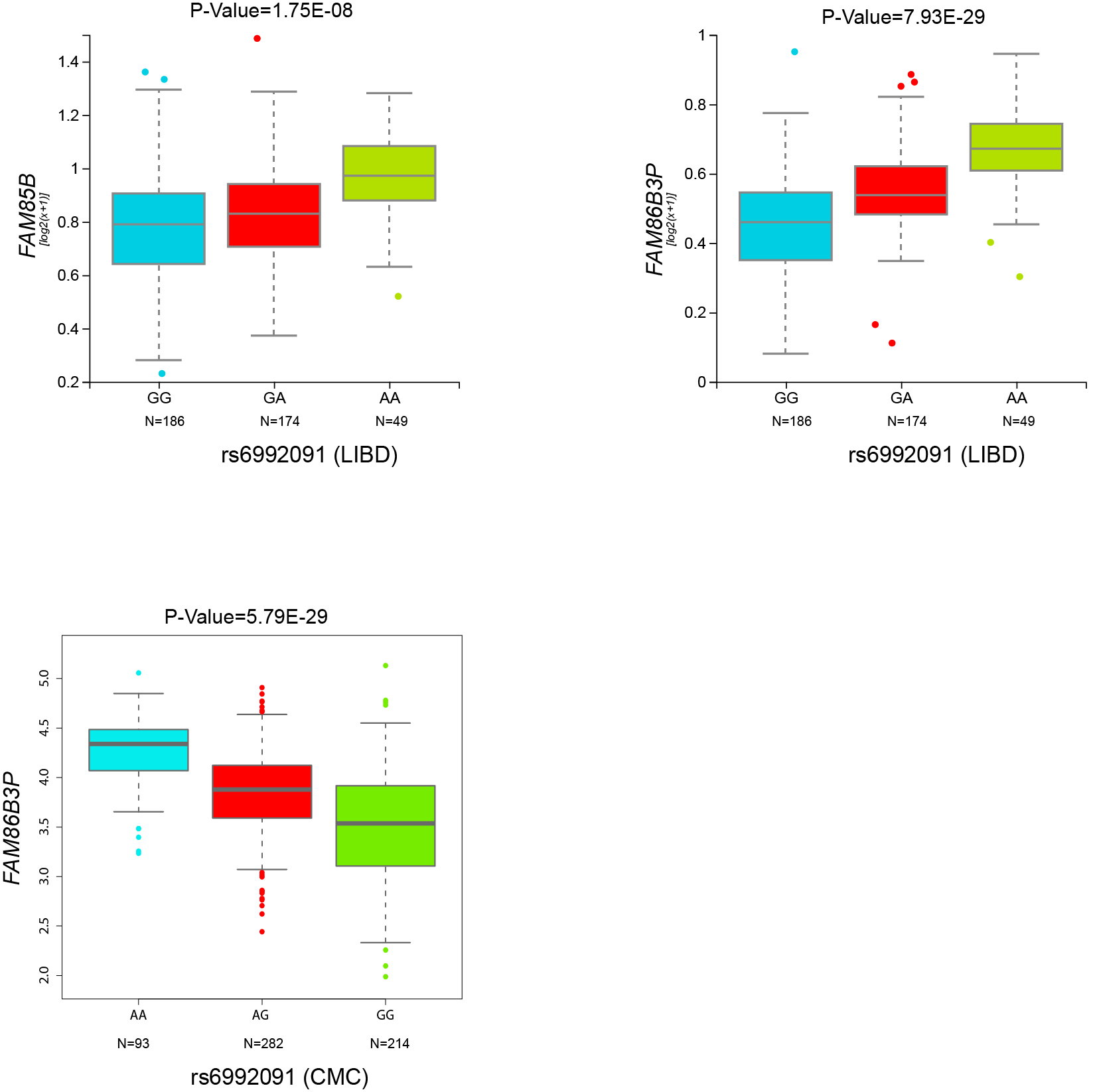

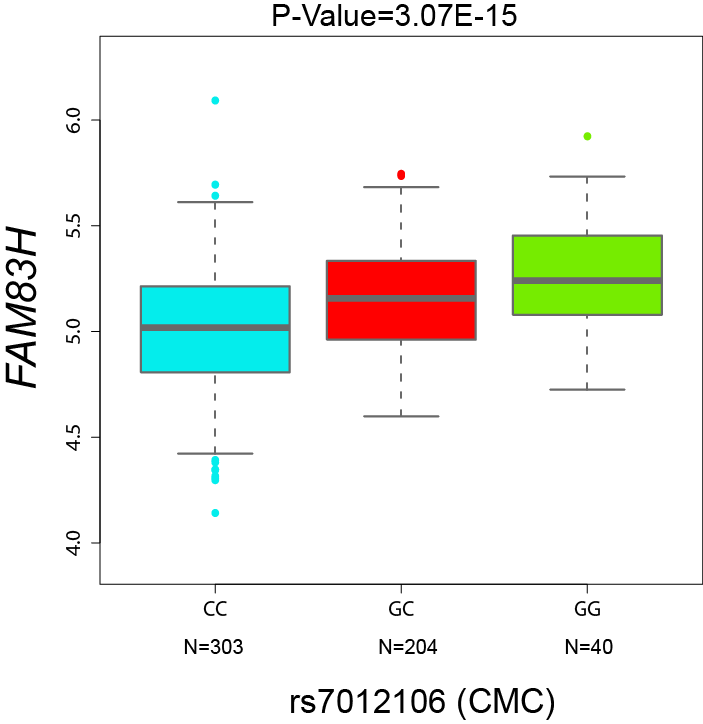

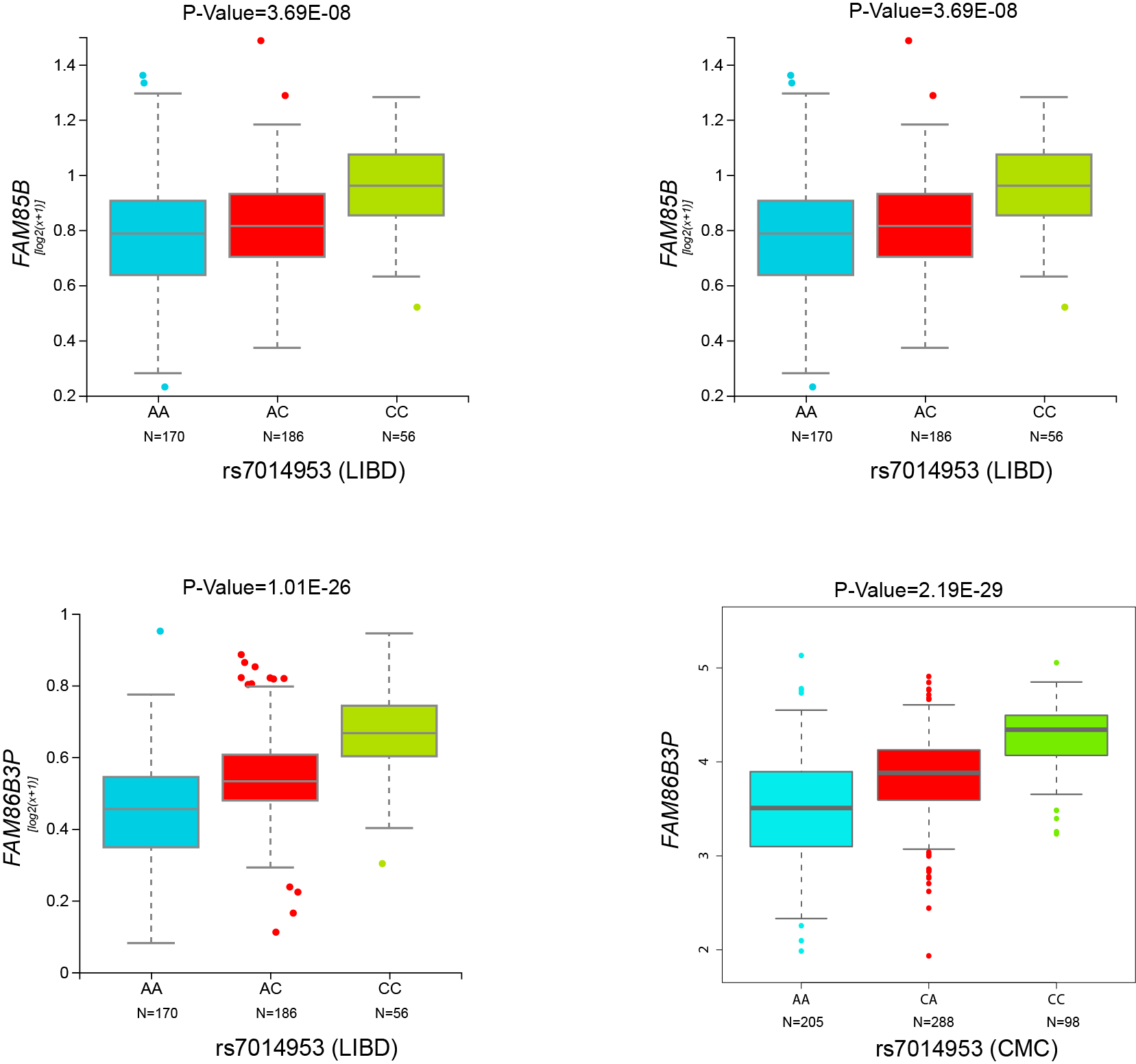

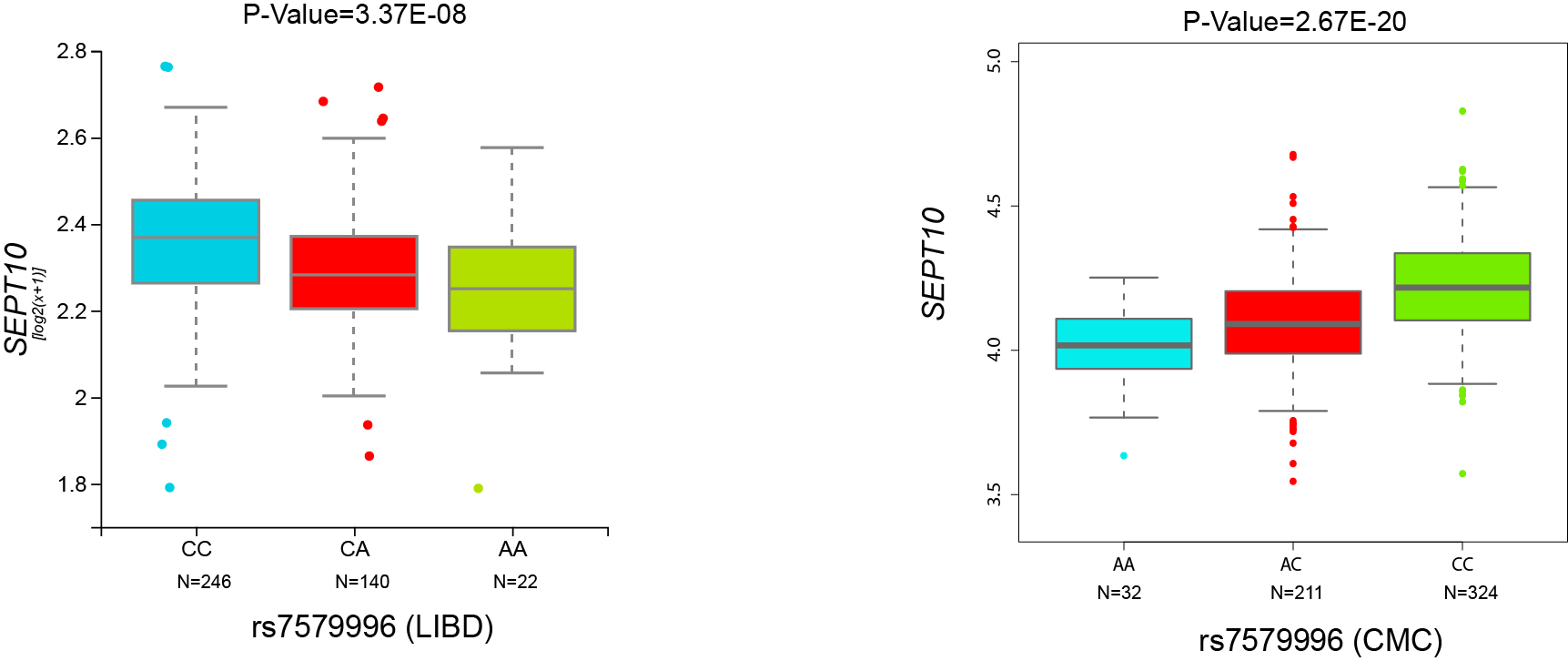

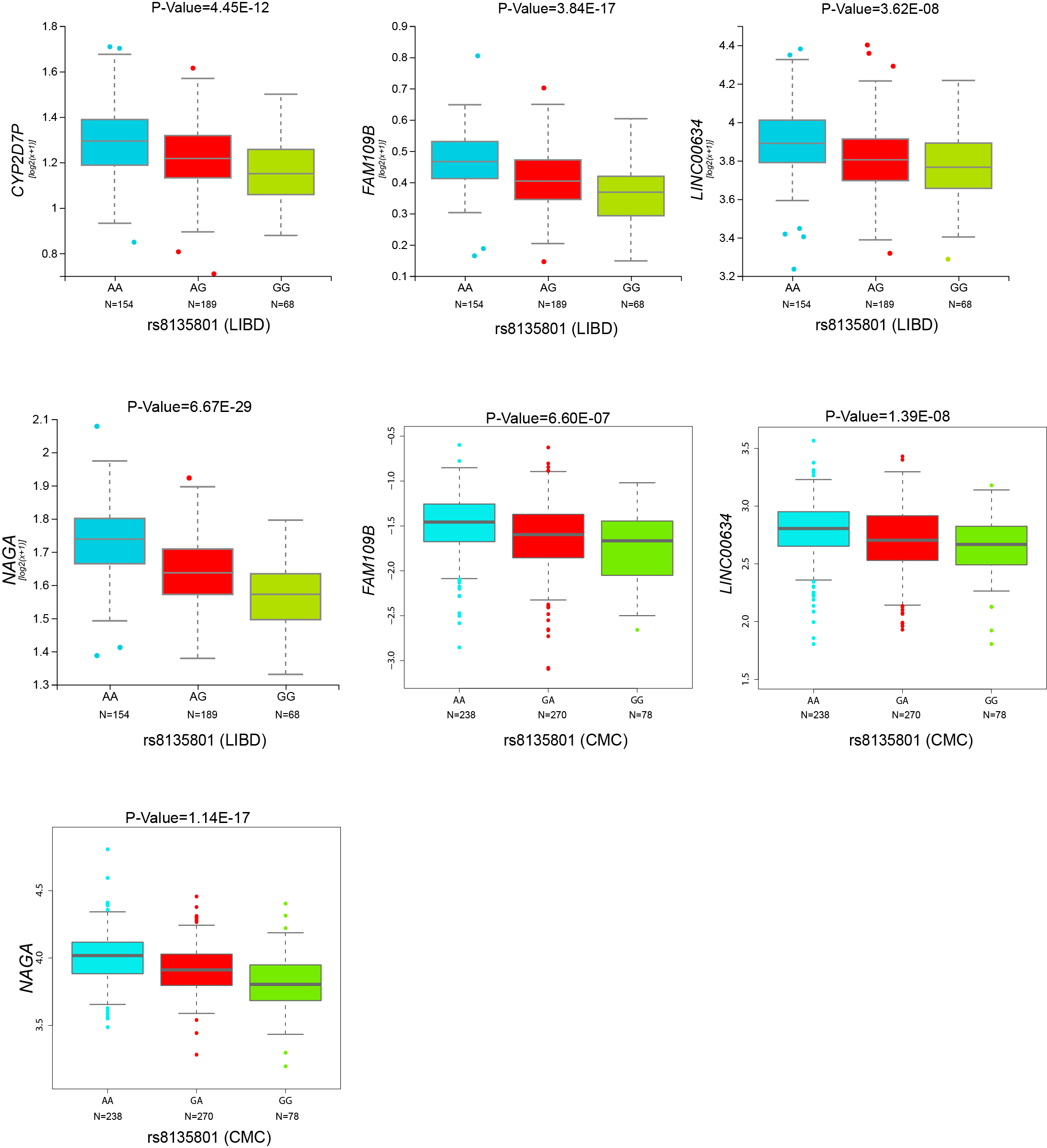

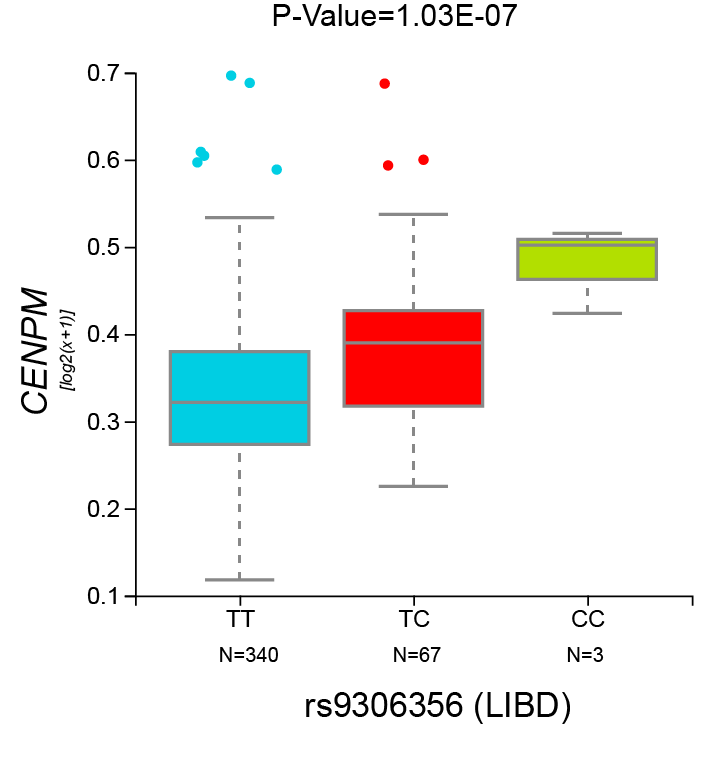

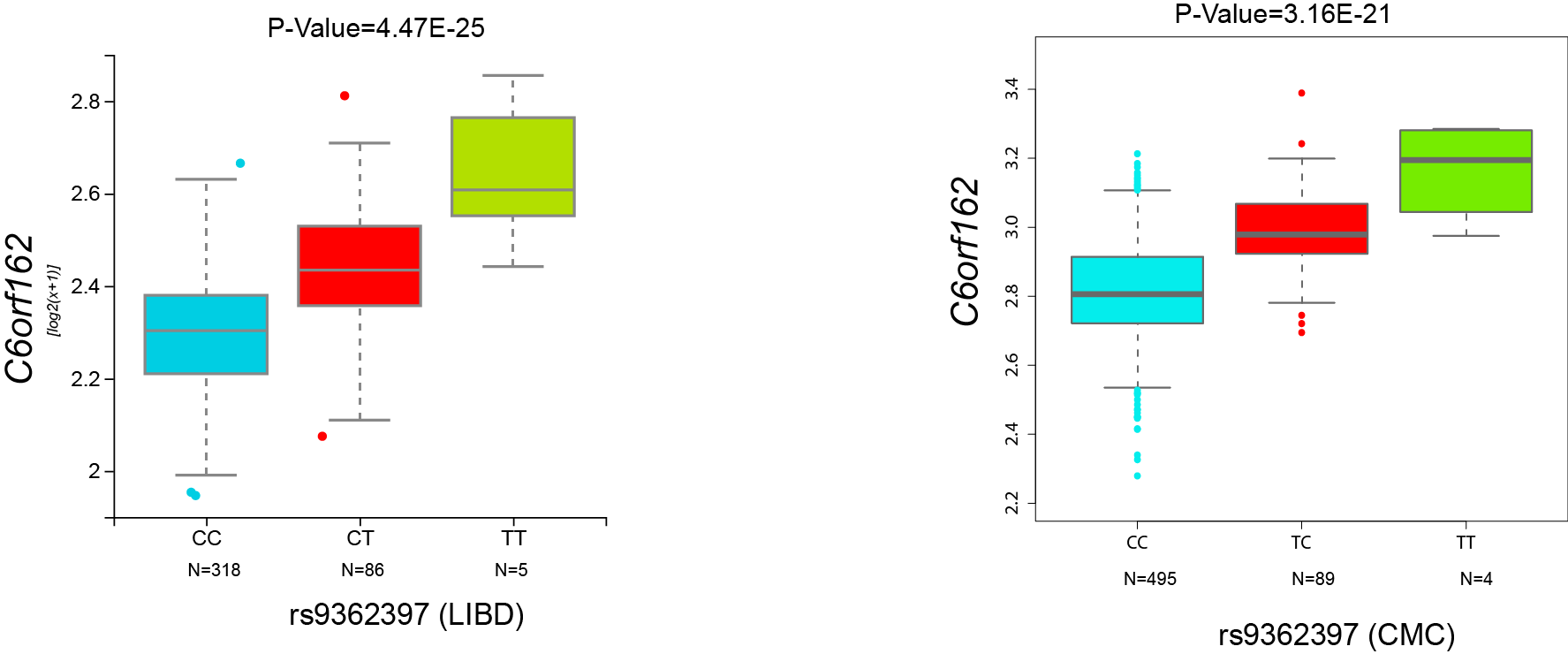

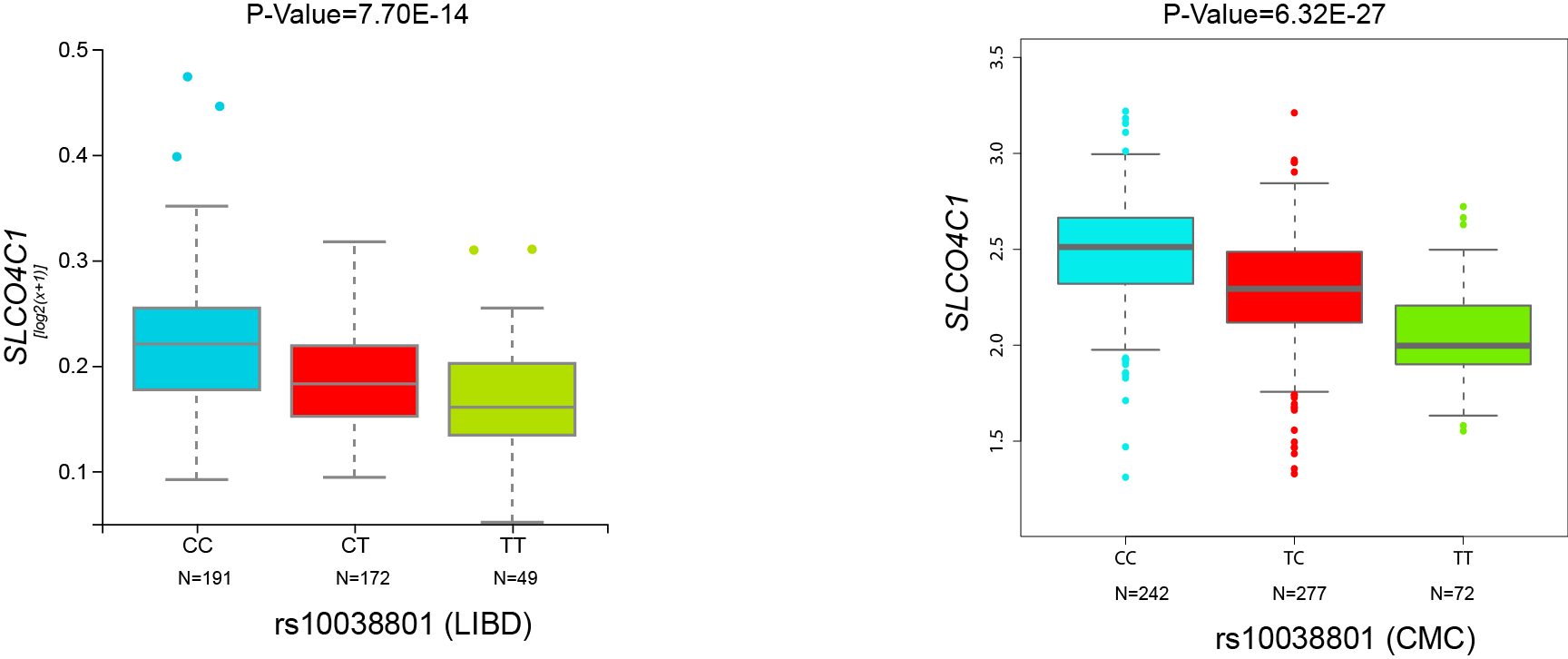

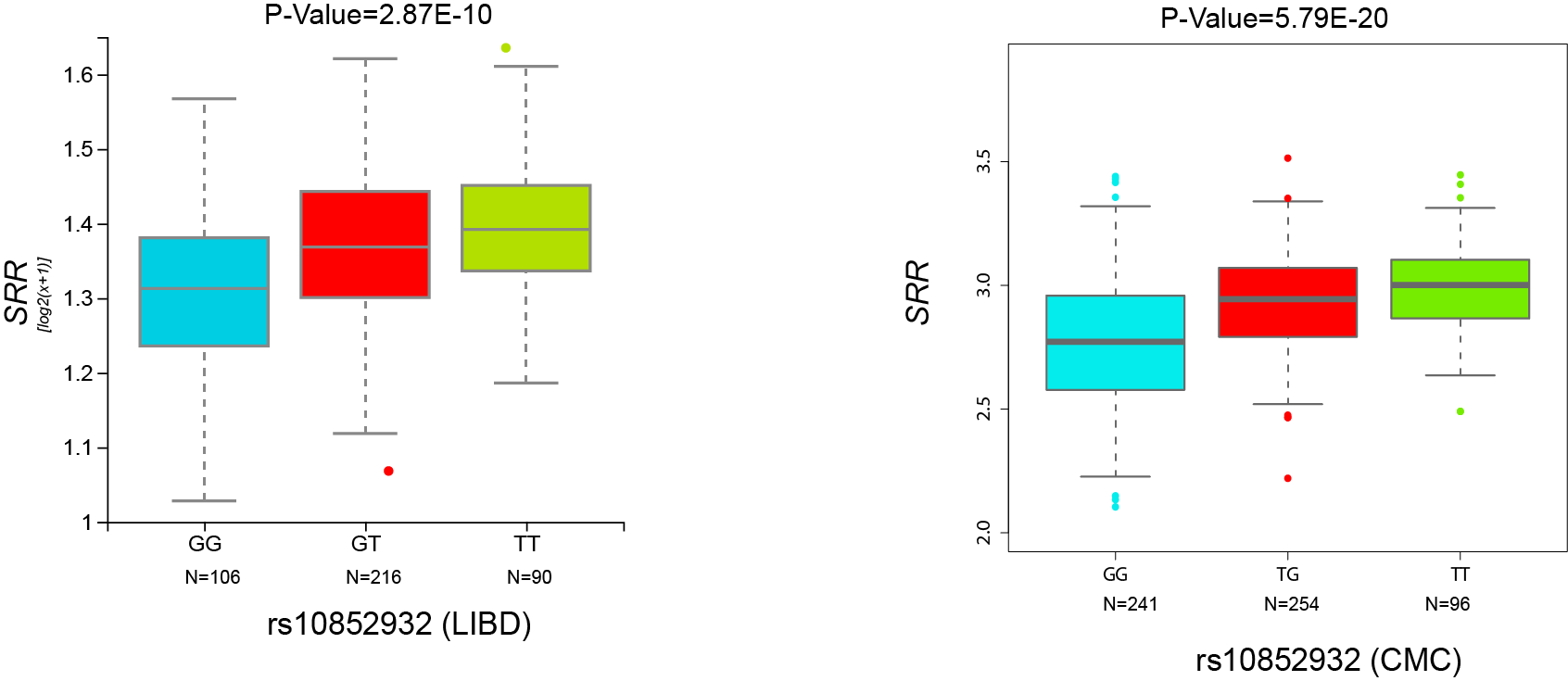

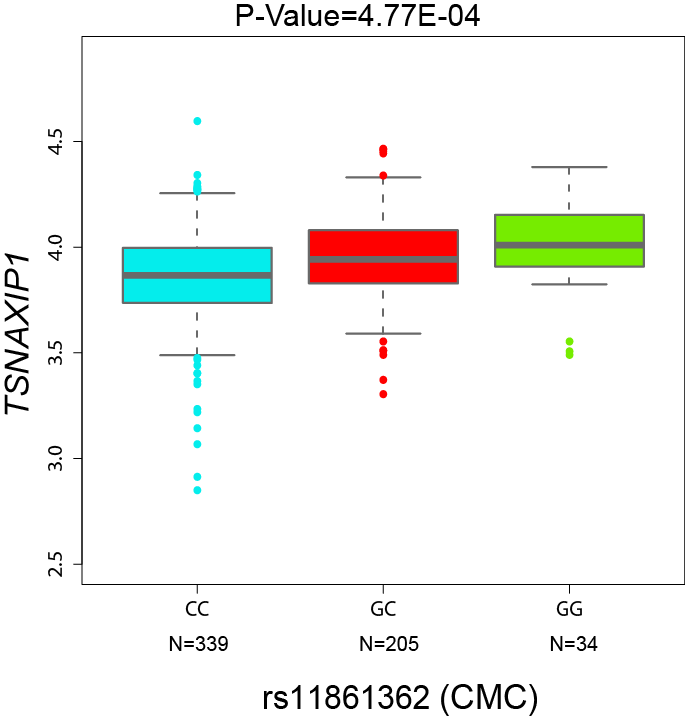

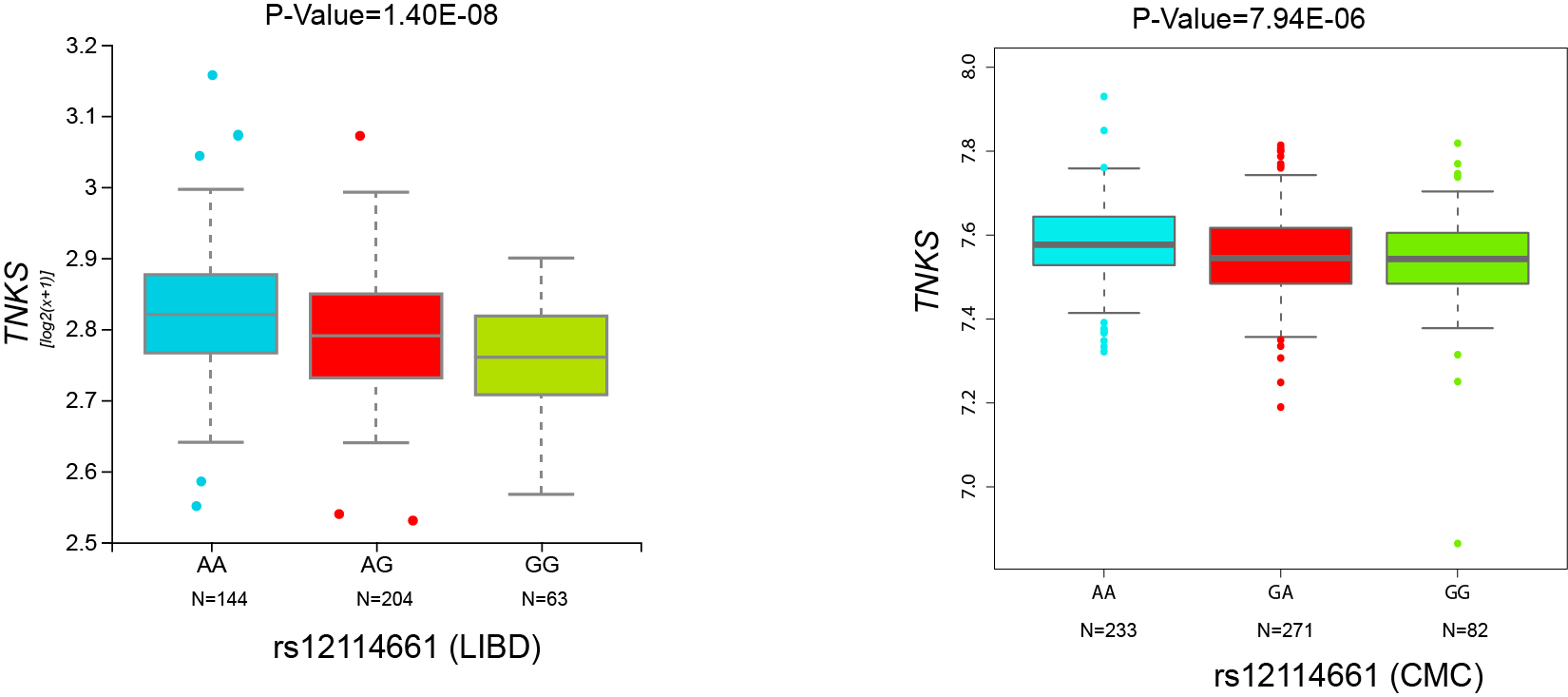

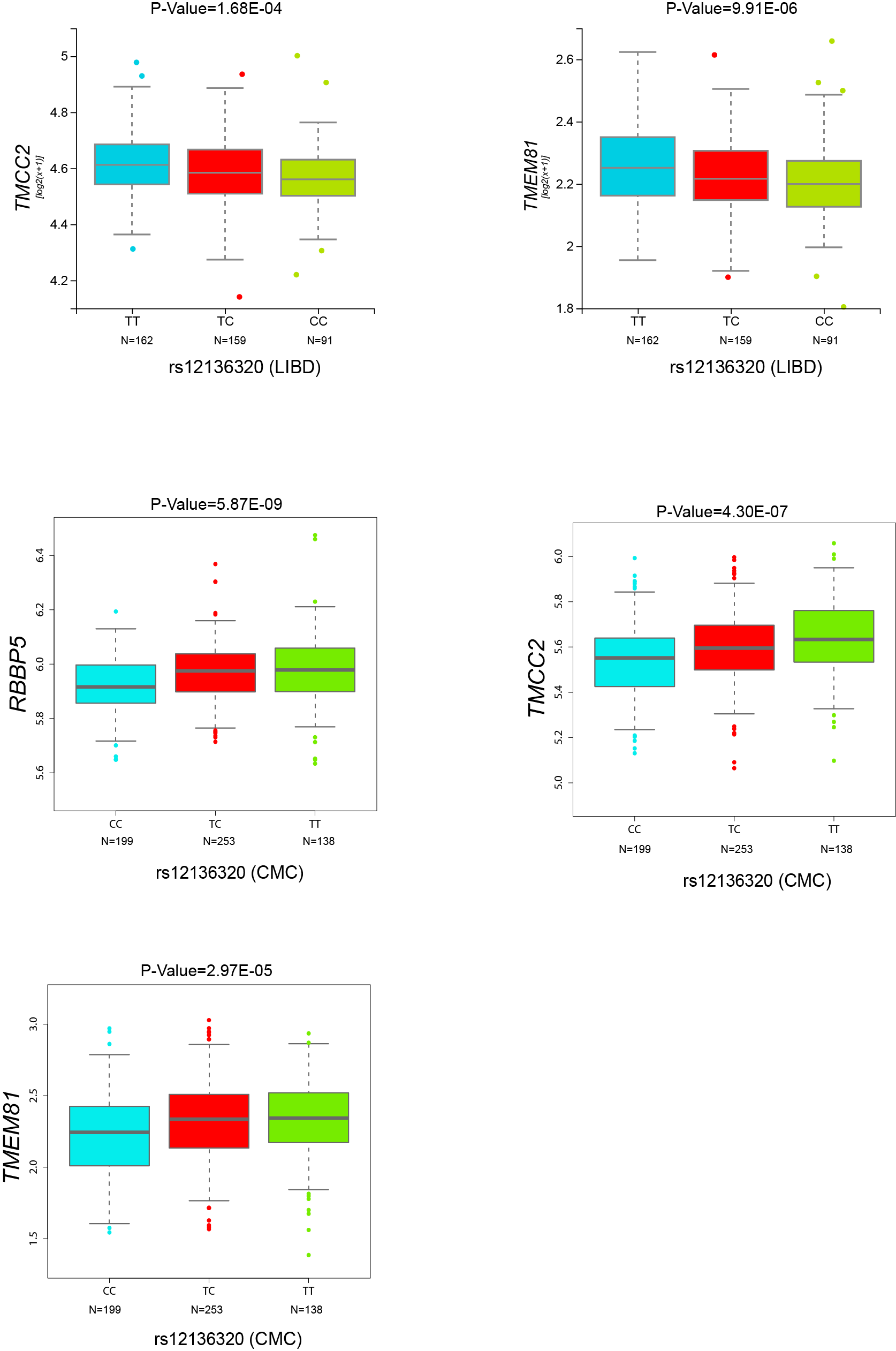

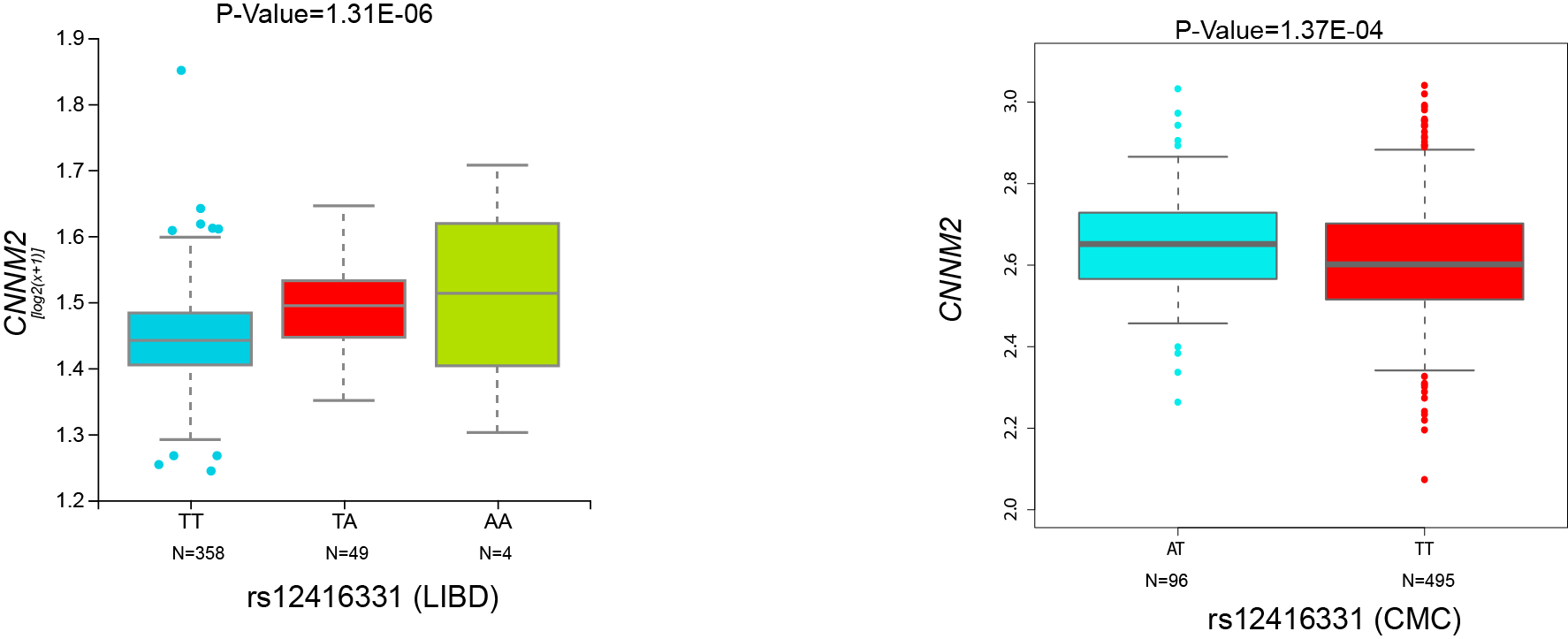

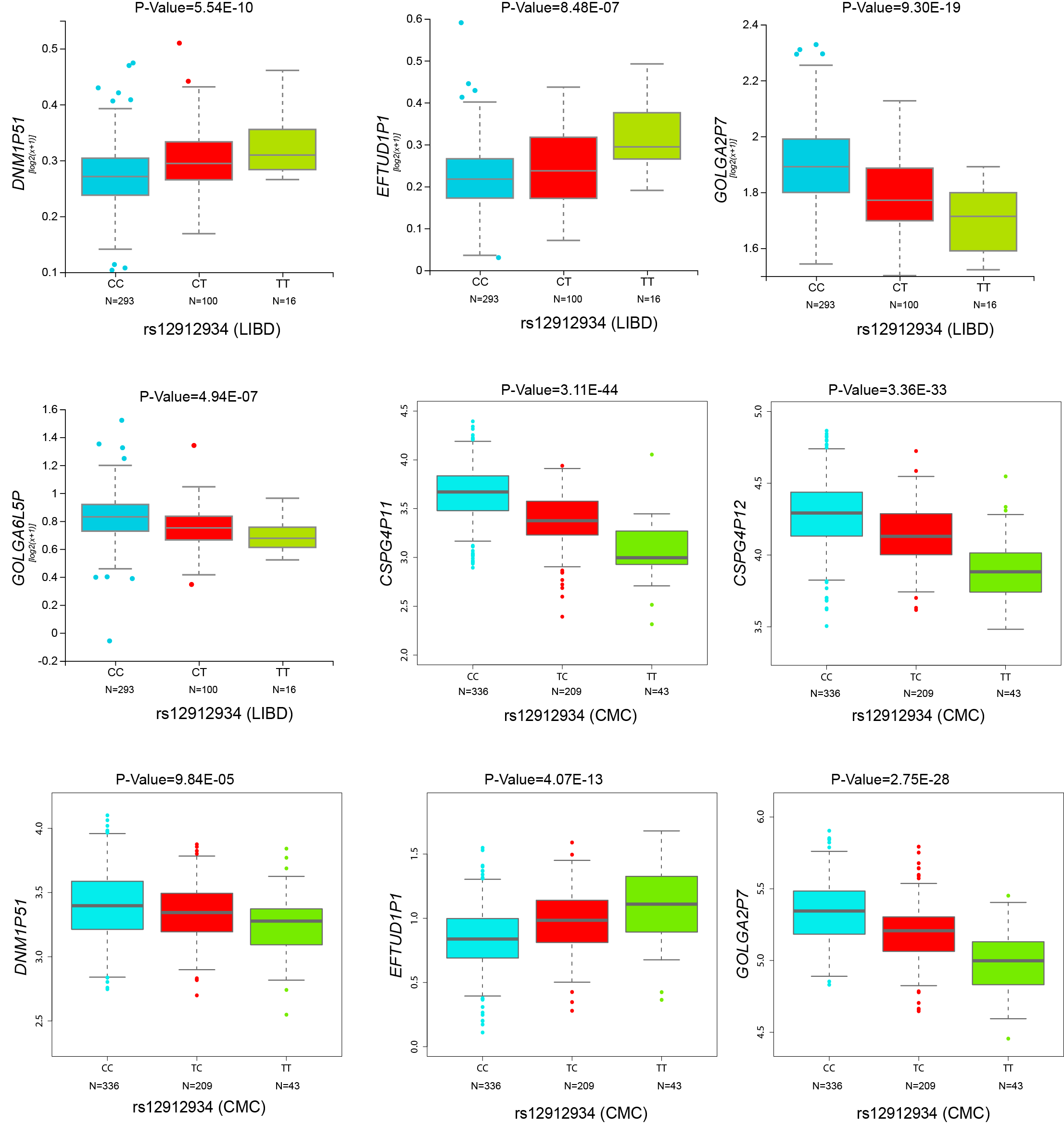

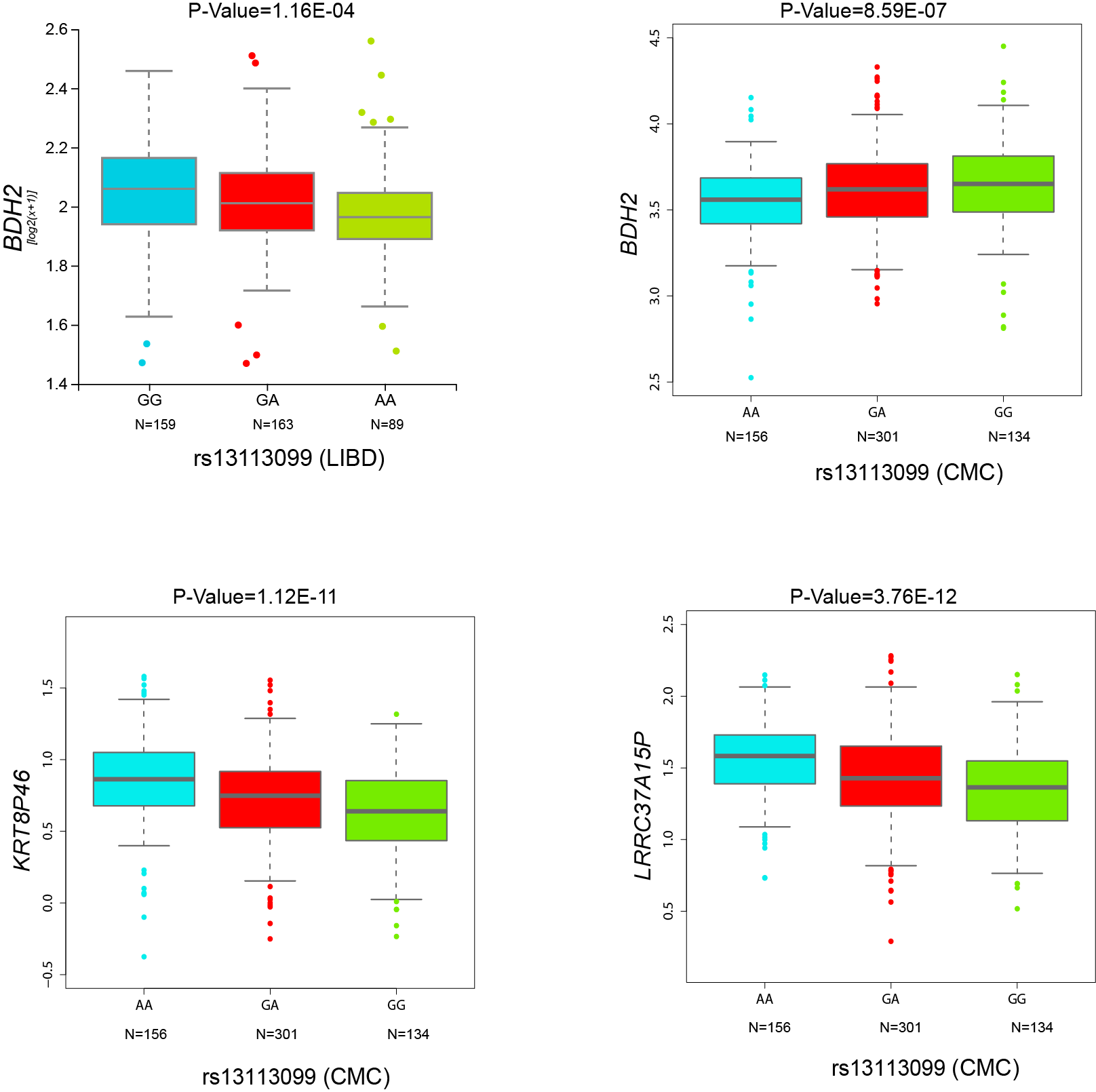

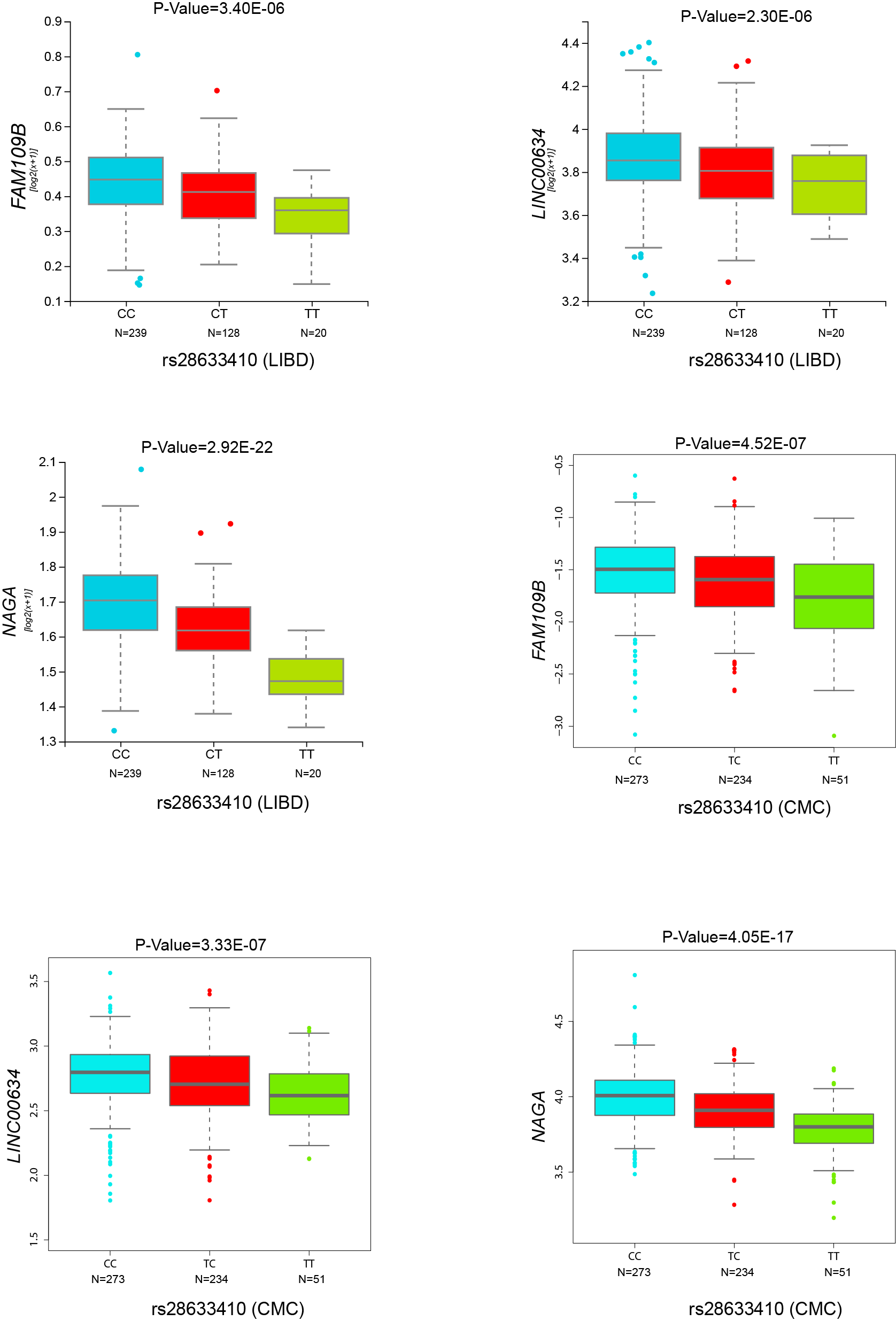

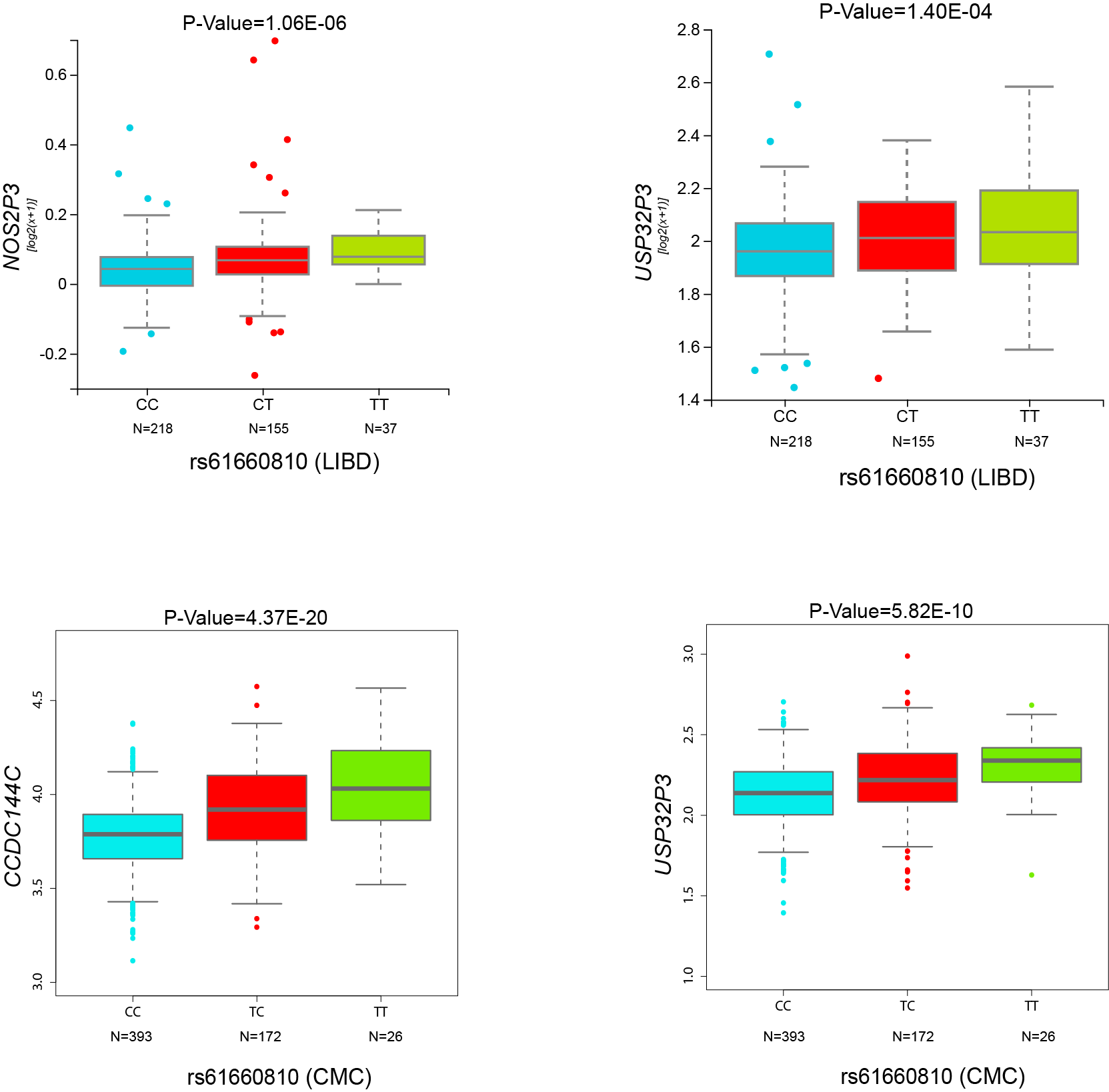

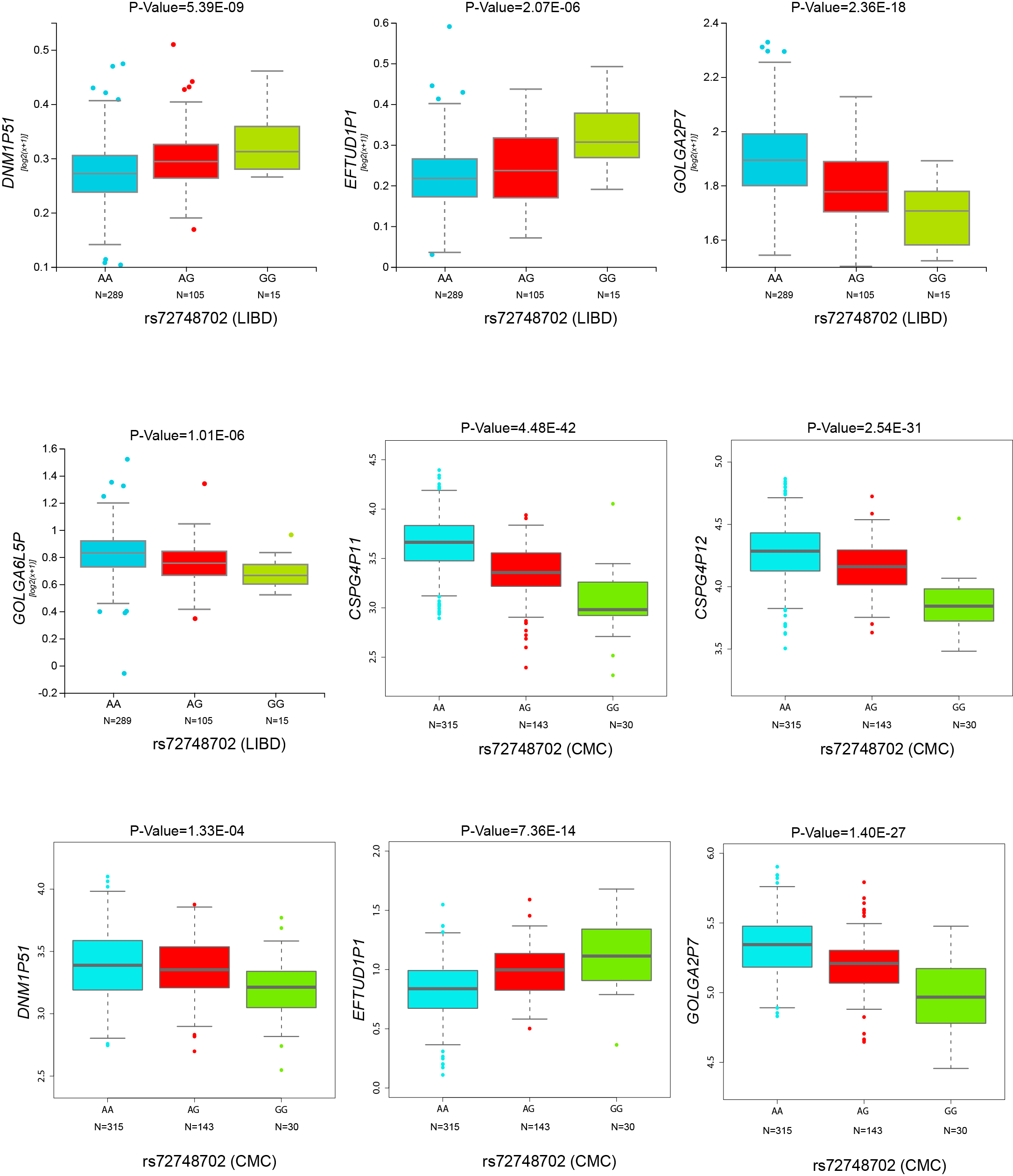

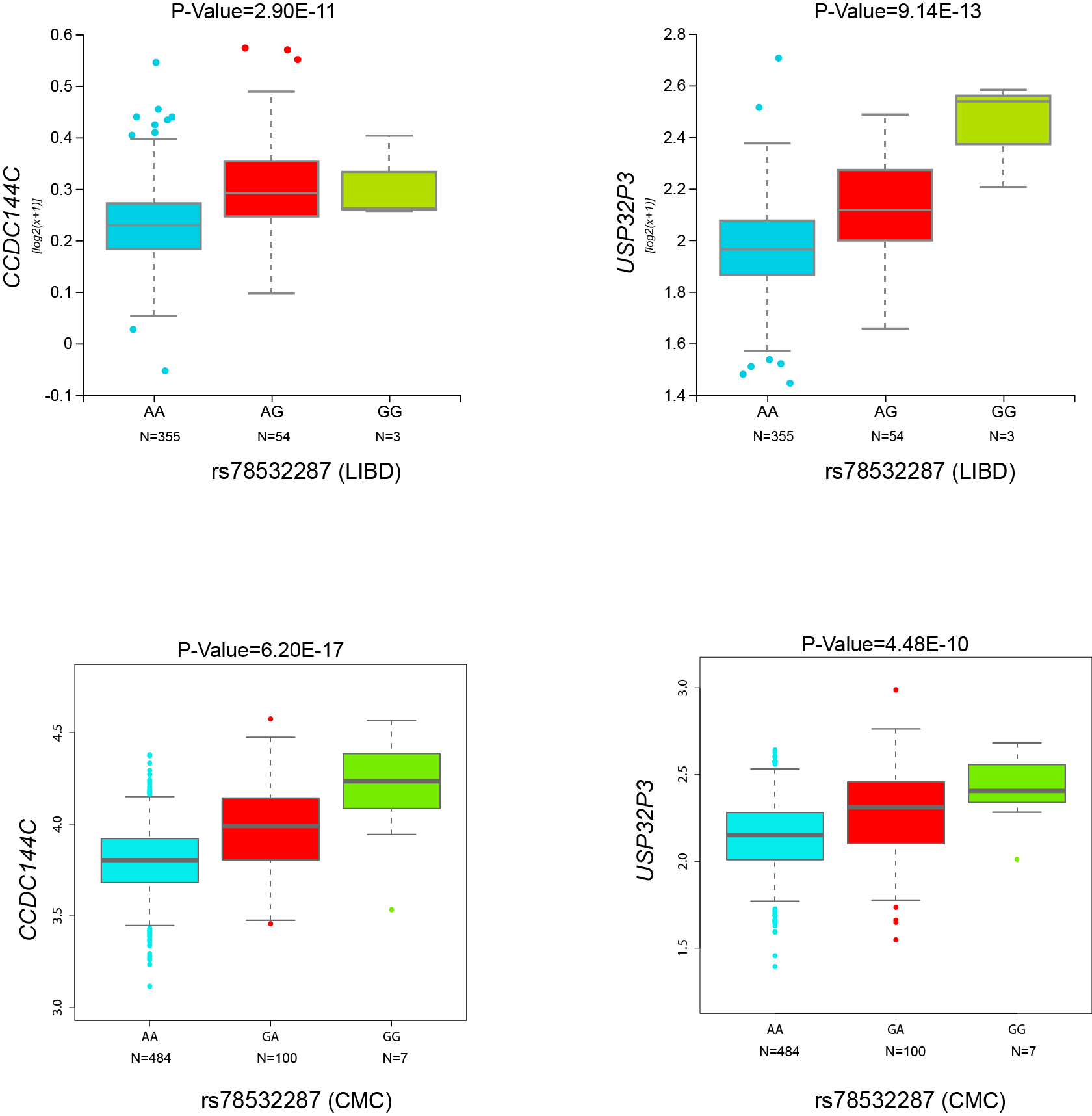

Supplement: Supplementary file 9 — Supplementary Data 6 [file 41467_2019_8666_MOESM9_ESM.docx]
